# Dexamethasone mitigates remdesivir-induced liver toxicity in human primary hepatocytes and COVID-19 patients

**DOI:** 10.1097/HC9.0000000000000034

**Published:** 2023-02-20

**Authors:** Kaiyan Liu, Sydney Stern, Emily L. Heil, Linhao Li, Rula Khairi, Scott Heyward, Hongbing Wang

**Affiliations:** 1 Department of Pharmaceutical Sciences, University of Maryland School of Pharmacy, Baltimore, Maryland, USA; 2 Department of Pharmacy Practice and Science, University of Maryland School of Pharmacy, Baltimore, Maryland, USA; 3 BioIVT, 1450 S Rolling Rd, Halethorpe, Maryland, USA

## Abstract

Coronavirus disease 2019 (COVID-19) is a global pandemic that has caused more than 600 million cases and over six million deaths worldwide. Despite the availability of vaccination, COVID-19 cases continue to grow making pharmacological interventions essential. Remdesivir (RDV) is an FDA-approved antiviral drug for treatment of both hospitalized and non-hospitalized COVID-19 patients, albeit with potential for hepatotoxicity. This study characterizes the hepatotoxicity of RDV and its interaction with dexamethasone (DEX), a corticosteroid often co-administered with RDV for inpatient treatment of COVID-19.

**Methods:**

Human primary hepatocytes and HepG2 cells were used as *in vitro* models for toxicity and drug-drug interaction studies. Real-world data from hospitalized COVID-19 patients were analyzed for drug-induced elevation of serum ALT and AST.

**Results:**

In cultured hepatocytes, RDV markedly reduced the hepatocyte viability and albumin synthesis, while it increased the cleavage of caspase-8 and caspase-3, phosphorylation of histone H2AX, and release of ALT and AST in a concentration-dependent manner. Importantly, co-treatment with DEX partially reversed RDV-induced cytotoxic responses in human hepatocytes. Moreover, data from COVID-19 patients treated with RDV with and without DEX co-treatment suggested that among 1037 patients matched by propensity score, receiving the drug combination was less likely to result in elevation of serum AST and ALT levels (≥ 3 × ULN) compared to the RDV alone treated patients (OR = 0.44, 95% CI = 0.22–0.92, p = 0.03).

**Conclusion:**

Our findings obtained from *in vitro* cell-based experiments and patient data analysis provide evidence suggesting combination of DEX and RDV holds the potential to reduce the likelihood of RDV-induced liver injury in hospitalized COVID-19 patients.

## Graphical abstract

**Figure:**
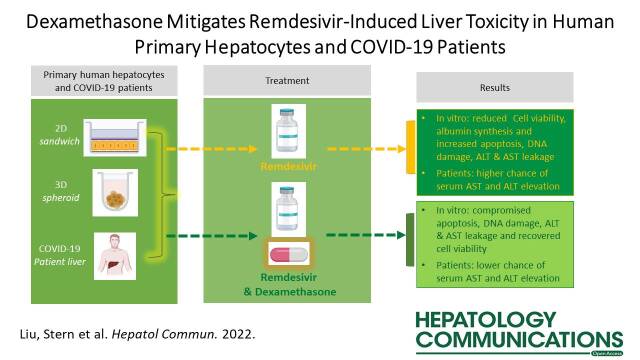

## INTRODUCTION

COVID-19, caused by severe acute respiratory syndrome coronavirus 2 (SARS-CoV-2), has rapidly become a global health crisis responsible for millions of deaths worldwide.^1^ Clinically, COVID-19 manifestations vary from mild symptoms such as fever, cough, and fatigue to severe conditions that could lead to prolonged ventilatory support, septic shock, and multi-organ failure.^2^,^3^ Although several COVID-19 vaccines have demonstrated effectiveness in lowering infection risk and mitigating symptom severity, current vaccination hesitancy and limited global vaccine accessibility, particularly in low-income countries, present major challenges in achieving global population protection. Moreover, the emerging SARS-CoV-2 variants and the explosive nature of COVID-19 transmission could outpace the capability of new vaccine development, necessitating the development and implementation of antiviral therapeutics to reduce transmission, prevent hospitalization, and decrease mortality in COVID-19 patients.

Remdesivir (RDV), a nucleotide analogue prodrug, is the first treatment for patients hospitalized with COVID-19 approved by the US Food and Drug Administration (FDA) in October 2020, with an expanded indication to outpatient use in January 2022. Initially developed as an anti-Ebola agent, RDV exhibits broad-spectrum activity against viruses from several families, including flaviviruses, Zika and Dengue viruses, and SARS-CoV-2.^4^–^7^ Mechanistically, RDV (GS-5734) undergoes initial hydrolysis mediated by carboxylesterase 1 (CES1) to an intermediate metabolite (GS-704277) followed by phosphorylation steps to form the pharmacologically active nucleoside triphosphate (GS-443902), which inhibits the viral RNA-dependent RNA polymerase activity and truncates extension of the viral genome.^5^,^8^ RDV has shown robust anticoronavirus activity in preclinical studies with the *in vitro* SARS-CoV-2 inhibition EC_50_ (50% maximal effective concentration) values at nanomolar to low micromolar ranges.^7^,^9^ Although clinical trials with RDV have had mixed outcomes, RDV has been associated with shortened recovery time when given early in the course of infection.^10^–^13^

Compared with the favorable effects of RDV in the management of COVID-19, relatively limited information is available regarding its safety profile and potential interactions with other co-administered drugs or preexisting medical conditions. Approximately 7% of COVID-19 patients treated with RDV experience the elevation of alanine aminotransferase (ALT) and aspartate aminotransferase (AST) levels in the blood, thus, requiring laboratory monitoring before and during RDV treatment; the risk of transaminase elevations with RDV has been listed in the FDA label under the Warnings and Precautions section.^14^,^15^ *In vitro* studies demonstrated that in comparison with extrahepatic cells, RDV exhibits markedly more sensitive cytotoxicity in the treated human liver cells.^16^ Several case reports suggest that RDV administration may have resulted in acute liver failure in COVID-19 patients with underlying medical comorbidities such as hypertension, diabetes, and hyperlipidemia.^14^,^15^,^17^

In addition to antiviral medications, dexamethasone (DEX), a synthetic corticosteroid and antiinflammatory agent, has been shown to decrease mortality in patients with COVID-19 requiring respiratory support.^18^,^19^ Adjuvant use of RDV and DEX further improved survival among hospitalized COVID-19 patients in population-based cohort studies.^20^ These findings led to an updated COVID-19 treatment guideline that recommends the combination of RDV and DEX for hospitalized COVID-19 patients requiring increasing quantities of supplemental oxygen.^20^,^21^ Notably, while this drug combination demonstrated a survival benefit in patients with COVID-19 mostly through alleviating respiratory symptoms, DEX is known to have profound effects on the liver,^22^ and the potential drug-drug interaction between DEX and RDV, particularly in the liver, is largely unknown.

In this study, we provide *in vitro* cell-based experimental results and real-world data that suggest that co-administration of DEX and RDV significantly reduced RDV-induced hepatotoxicity. In human primary hepatocytes (HPH) cultured in 2-dimensional (2D) sandwich and 3-dimensional (3D) spheroid formats, cytotoxic responses, including alteration of the cell viability, albumin synthesis, caspase-3, -8, and -9 cleavage, histone H2A X variant (H2AX) phosphorylation, and ALT/AST leakage, were analyzed upon the exposure of RDV and/or DEX. An observational cohort study of RDV/DEX-associated liver injury was conducted in hospitalized adult patients with COVID-19.

## EXPERIMENTAL PROCEDURES

### Chemicals and biological reagents

RDV was obtained from Bio-Techne Corporation. Dexamethasone and DMSO were purchased from Sigma-Aldrich. Matrigel and insulin/transferrin/selenium (ITS^+^) were obtained from BD Biosciences Discovery Labware. Other cell culture reagents were purchased from ThermoFisher Scientific or Sigma-Aldrich.

### Human primary hepatocytes cultures and treatment

HPH obtained from BioIVT (Baltimore, MD) with ≥ 90% viability were seeded at 0.75×10^6^ cells/well or 0.06 × 10^6^ cells/well in 12-well and 96-well biocoat plates in BioIVT INVITROGROTM CP Medium following the company’s instructions. Twelve hours after attachment, hepatocytes were cultured in complete William’s Medium E without DEX and overlaid with Matrigel (0.25 mg/mL) as described previously.^23^ Cultured HPH were pretreated with solvent (0.1% DMSO) or DEX (1 µM) for 4 h, followed by co-treatment of RDV (1, 5, 10, 15, 20, 40, 50 µM) with or without DEX (1 µM) for 24 h or 48 h before harvesting for subsequent analyses. These concentrations of RDV were chosen based on the reported Cmax value (3.7–4.5 µM),^24^,^25^ and cover a full-spectrum range both lower and higher than the Cmax in cell viability assays. For 3D spheroid culture, HPH and nonparenchymal cells (NPCs) obtained from BioIVT were seeded into 96-well ultra-low attachment plates (Corning, Glendale, AZ) at a 4: 1 ratio of hepatocyte: NPC (1500: 375) in BioIVT INVITROGROTM CP Medium and centrifuged at 250×g for 2 min. From day 5 (with the seeding date as day 1) after spheroids were aggregated and compact, 50% of the culture medium was changed to serum-free complete William’s Medium E every other day. Drug treatments, as indicated above, were initiated on day 10. Cell viability and live cell staining assays were conducted as outlined below. The demographic information of liver donors is included in Supplemental Table S1, http://links.lww.com/HC9/A95.

### Hepatoma cell culture and treatment

HepG2 cells purchased from the American Tissue Culture Collection were cultured in Dulbecco’s Modified Eagle’s Medium supplemented with 10% fetal bovine serum, 100 units/mL penicillin, and 100 µg/mL streptomycin at 37°C and 5% CO2 in a humidified incubator. Cultured cells were then treated with RDV (1, 5, 10, 15, 20, 40, 50, 80 µM) with or without DEX (1 µM) for 48 h, before cell viability examination. The authenticity of HepG2 cells was confirmed by short tandem repeat profiling analysis.

### Cell viability

Cell viability was assessed using the CellTiter-Glo Luminescent Cell Viability Assay kit or CellTiter-Glo 3D Cell Viability Assay kit from Promega Corporation following the manufacturer’s instructions. Briefly, after treatment, an equal volume of CellTiter-Glo Reagent was added to the cell culture medium in each well and mixed thoroughly on an orbital shaker at the maximum speed for 5 min. Subsequently, the mixture was incubated at room temperature for 20 min to stabilize the luminescent signal. The luminescent signal was measured using the GLOMAX 96 microplate luminometer. GraphPad Prism software 8.0.0 was used for CC_50_ calculation.

### ALT/AST activity

Cell culture medium was collected after drug treatments and subjected to the detection of ALT and AST by using the ALT and AST Activity Assay Kits from Sigma-Aldrich. Briefly, after preparing the cell medium and reaction mix following the manufacturer’s instructions in a 96-well flat-bottom plate, the mixture-containing plate was incubated at 37°C. The absorbance at 570 nm (ALT) or 450 nm (AST) was measured every 5 min and lasted for 1 h on the SpectraMax M5 (San Jose, CA). ALT and AST activities were calculated using the formula listed in the assay kits.

### Immunofluorescence analysis

Forty-eight hours after treatment, HPH was washed with phosphate-buffered saline, fixed with 4% formaldehyde for 15 min, permeabilization with 0.5% Triton X-100 for 10 min, blocked with 10% fetal bovine serum for 30 min, and incubated with anti-albumin (sc51515, 1:100, Santa Cruz, USA) or anti-γ-H2AX (JBW301, 1:100, Sigma-Aldrich) antibodies at 4°C overnight. Subsequently, cells were incubated with fluorescent dye-labeled secondary antibodies, Alexa Fluor 488 for albumin and Alexa Fluor 594 for γ-H2AX for 1 h, respectively. A mounting medium containing DAPI was added, and images were visualized using a Nikon Eclipse Ti-E inverted fluorescence microscope (Nikon, Edgewood, NY). The amount of albumin and γ-H2AX was quantified as mean integrated intensity using bright spot detection from 3 whole wells and normalized to the vehicle control (0.1% DMSO).

### Western blot assay

Cell homogenate proteins (40 µg) were resolved on SDS-polyacrylamide gels (12%) and then electrophoretically transferred onto polyvinylidene fluoride membranes. Subsequently, membranes were blocked with 5% milk and incubated with antibodies against caspase-3 (1:500, #9661S, Cell Signaling Technology, Danvers, MA), caspase-8 (1:1000, #9496, Cell Signaling Technology), caspase-9 (1:1000, #9505, Cell Signaling Technology), or beta-actin (1:3000; A3854, Sigma-Aldrich) at 4°C overnight. Blots were washed and incubated with horseradish peroxidase secondary antibodies and developed with West Femto chemiluminescent substrates (ThermoFisher, Rockford, IL).

### Live-Dead cell imaging

Spheroids cultured in 96-well ultra-low attachment plates were treated with vehicle control (0.1% DMSO) or RDV (10 µM) with and without DEX (1 µM) for 48 h. After drug treatment, spheroids were incubated with a fluorescence dye-medium mixture containing NucBlue Live ReadyProbes Reagent, NucRed Live 647 ReadyProbes Reagent, or Calcein-AM (Thermofisher) for 30 min at 37°C as indicated by the manufacturer’s instructions. Fluorescence images were captured and analyzed using the Celigo Imaging Cytometer-4 Channels system from Nexcelom Bioscience LLC (Lawrence, MA). The dead/live ratio was quantified as the integrated intensity of the dead stain compared with the live stain using brightspot detection for each spheroid.

### Real-time apoptosis analysis

On day 2 of the HPH sandwich culture, the culture medium containing IncuCyte Caspase-3/7 Green Dye (1:2500 dilution) from Essen BioScience was used at the beginning of drug treatment. Real-time apoptosis was monitored using a time-lapse imaging system, IncuCyte@ S3 Live-Cell Analysis System every 4 h for a total of 60 h. Activation of caspase-3/7 generated green fluorescence was normalized to real-time cell confluence. Relative fluorescence intensity was analyzed using the ratio of green/brightfield from 3 whole wells and normalized to the vehicle control (0.1% DMSO).

### Evaluation of AST/ALT elevations in COVID-19 patients

To evaluate if our *in vitro* findings are correlated with real-world patient data, electronic health records of patients with severe COVID-19 requiring hospitalization and receiving RDV with or without DEX from May 1, 2020 to May 30, 2021 were obtained from 4 hospitals in the University of Maryland Medical System. An exemption has been determined and approved by the University of Maryland, Baltimore Institutional Review Board. The primary exposure was treatment with RDV or a combination treatment of RDV and DEX within 72 h of each other. The primary outcome was dichotomized as maximum AST and ALT ≥ 3×ULN (≥ 147 UI/L) or <3×ULN (<147 UI/L) within 10 days of RDV treatment. The ≥ 3×ULN cut-off was selected based on the FDA’s Guidance for Industry: DILI.^26^ The collection of electronic health records data for research was approved by the institutional review board of the University of Maryland School of Medicine. Adult patients with laboratory-confirmed SARS-CoV-2 treated with RDV for at least 48 hours for COVID-19 were included. A detailed description of the study design and procedure is included in the Supplementary Section.

### Statistical analysis

For *in vitro* cell-based experiments, all data represent mean±SD from at least 3 independent experiments. Statistical comparisons were obtained using 1-way ANOVA followed by a *post hoc* Dunnett test for > 2 groups or Student t test for 2 groups. Besides, 1-way related measures ANOVA followed by Tukey posttests or 2-way related measures ANOVA followed by Bonferroni posttests were used when appropriate (GraphPad Prism 8.0.0). Statistical significance was set at *p*<0.05.

In the patient data analysis, propensity scores were computed based on the following covariates to help balance the RDV and RDV and DEX groups: race, preexisting liver disease, the all patient refined diagnosis related group severity of illness (APR DRG SOI) score (a measurement of severity of illness upon hospital admission based on organ function, damage, and physiological decomposition), and concomitant potentially hepatotoxic medications to account for the differences in patients receiving RDV alone versus RDV and DEX. Patients receiving RDV alone were matched 1:5, using the nearest neighbor match method with a 0.2 caliper, with patients receiving RDV and DEX. RDV-treated patients were matched with RDV and DEX co-treated patients. Using this matched cohort, the association between patients treated with RDV versus RDV and DEX was tested using conditional logistic regression adjusted for age and ethnicity for binary outcomes of the above or below AST/ALT 3×ULN. OR for dichotomous variables with a 95% CI were calculated. A 2-sided *p* value of less than 0.05 was regarded as statistically significant. Statistical analyses were performed using SAS v. 9.4 (SAS Institute Inc., Cary, NC).

## RESULTS

### DEX reduces RDV-induced cytotoxicity in human primary hepatocytes

**FIGURE 1:**
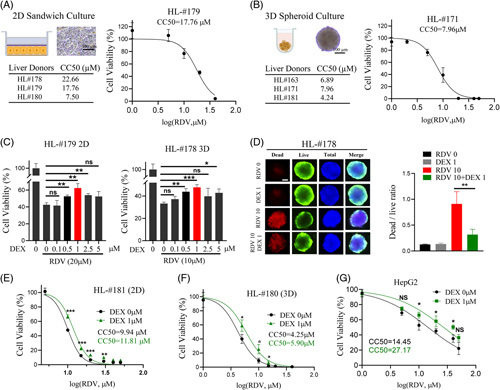
Dexamethasone reduces remdesivir-induced cell death in human hepatocytes and HepG2 cells. Human primary hepatocytes cultured in sandwich (2D) or spheroid (3D) format were treated with RDV at indicated concentrations for 48 h. Cell viability was measured as outlined in “Methods” in treated 2D (A) and 3D (B) hepatocytes. CC_50_ values were estimated in treated hepatocytes obtained from multiple liver donors (HL#171, HL#163, and HL#178-181). Cell viabilities were also tested in hepatocytes from donors HL#179 (2D culture) and HL#178 (3D culture) treated with RDV (20 µM 2D; 10 µM 3D) with and without DEX at indicated concentrations for 48 h (C). Live and Dead cell staining was conducted in 3D cultured hepatocytes from HL#178 (D) treated with RDV (10 µM) with or without DEX (1 µM) for 48 h. Live cells were stained in green, dead cells in red, and total cells in blue. Images were taken using Nikon fluorescence microscopy. Scale bars represent 100 µm. The ratio of dead/live cells was quantified as detailed in “Methods”. In separate experiments, human hepatocytes in 2D (E) and 3D (F), as well as HepG2 cell (G) cultures were treated with RDV at indicated concentrations in the presence and absence of DEX (1 µM) for 48 h. CC50 were calculated as mentioned above. Data are presented as mean±SD, n =3. Statistical significance was set at *p < 0.05; **p < 0.01; ***p < 0.001.

We first determined the cell viability changes induced by RDV using HPH cultured in a 2D sandwich format and a 3D human liver spheroid configuration constructed by co-culturing HPH and NPCs. In both culture models, RDV concentration-dependently reduced the cell viability with the 50% cytotoxic concentration (CC_50_) ranging from 7.5 to 22.66 µM in sandwich-cultured HPH from liver donors (HL#178, #179, #180) and 4.24 to 7.96 µM in 3D spheroids (HL#163, #171, #181), respectively (Figure 1A and B).

To examine whether DEX affects RDV-induced hepatotoxicity, human hepatocytes were co-treated with multiconcentrations of DEX and RDV at 20 µM (2D) or 10 µM (3D) as detailed in “Methods”. The RDV concentrations were selected based on the CC_50_ values. Figure 1C shows that DEX at 1 µM exhibited maximal hepatocyte protection from RDV-induced cytotoxicity in both culture formats. A higher concentration of DEX (5 µM), while not directly leading to a decrease in cell viability, did increase caspase-3 cleavage and minimized its protection against RDV-induced apoptosis (Supplemental Fig. S1, http://links.lww.com/HC9/A94). Thus, 1 µM of DEX was used in subsequent studies. This protective effect was further illustrated in hepatocyte spheroids (HL#178), where in comparison to RDV treatment alone, RDV plus DEX led to a decrease in the dead (red)/ live (green) cell ratio (Figure 1D). In separate experiments, co-treatment of DEX (1 µM) and RDV at indicated concentrations resulted in the shift of the RDV CC_50_ values from 9.94to 11.81 µM in 2D hepatocytes, 4.25 to 5.90 µM in 3D hepatocyte spheroids, and 14.45 to 27.17 µM in HepG2 cells, respectively (Figure 1E, F, and G). A similar trend of DEX diminishing RDV cytotoxicity was also observed in additional liver donors (Supplemental Fig. S2, http://links.lww.com/HC9/A93). Notably, optimal reduction of RDV-mediated cytotoxicity by DEX was achieved at RDV exposure levels between 5 and 20 µM, while diminished when RDV concentration exceeds 40 µM. Together, these observations indicate that RDV is toxic to human hepatocytes and hepatoma cells, and 3D spheroids appear to be more sensitive to RDV than the 2D culture format. Importantly, co-treatment with DEX protects RDV-induced cytotoxicity in all cultured liver cells.

### DEX attenuates RDV-induced apoptosis and DNA damage in human hepatocytes

**FIGURE 2:**
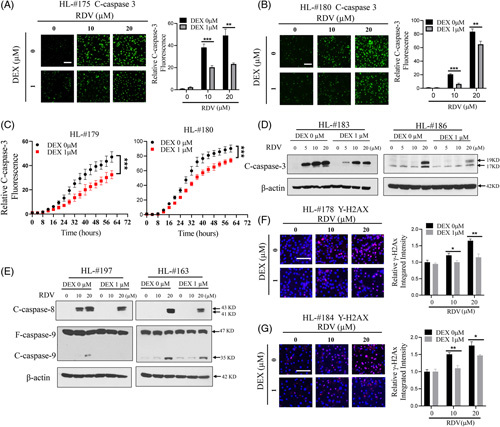
Dexamethasone attenuates apoptosis and DNA damage induced by RDV in human hepatocytes. Sandwich-cultured human hepatocytes were treated with vehicle control (0.1% DMSO), RDV (10 µM and 20 µM) with or without DEX (1 µM) for 48 h. Caspase-3/7 activity was visualized with green fluorescence generated from the cleavage of a caspase-3/7 substrate in an IncuCyte Live Cell Analysis System. Real-time monitoring of the caspase-3/7 activity was conducted using the time-lapse auto-imaging every 4 h for a total of 60 h. Caspase-3/7 staining images and relative intensity were compared after 48 h treatment in liver donors HL#175 (A) and HL#180 (B). Time-dependent caspase-3/7 activation was compared between RDV alone and RDV co-treated with DEX (1 µM) (C) in HL#179 and #180. Cleaved caspase-3 protein was analyzed using Western blotting in hepatocytes from HL#183 and #186. The antibody against cleaved caspase-3 detects the 19 KD and 17 KD cleavage products of activated caspase (D). Under the same treatment of HPH from liver donors HL#163 and HL#197, cleaved caspase-8 and caspase-9 proteins were analyzed using Western blotting (E). In another experiment, the phosphorylated H2AX, γ-H2AX, was probed following the treatment of RDV and DEX as indicated in hepatocytes from HL#178 (F) and HL#184 (G) for 48 h. Relative γ-H2AX fluorescence intensity was imaged using a fluorescence microscope, and integrated intensity was calculated as outlined in “Methods”. The scale bars represent 100 µm. Data are presented as mean±SD obtained from three separate experiments. Statistical significance was set at *p < 0.05; **p < 0.01; ***p < 0.001.

The initiation of apoptosis and inducing DNA damage are common features shared by many toxic compounds, inducing cellular injury and cell death. Here we examined whether RDV treatment induces hepatocyte caspase-3 cleavage and H2AX phosphorylation, 2 commonly used parameters for apoptosis and double-strand DNA damage.^27^,^28^ Our data showed that RDV at 10 µM and 20 µM markedly induced the fragmentation of caspase-3 in cultured HPH (HL#175, #180), while this induction was significantly ameliorated by 1 µM of DEX (Figure 2A, B). The temporal profile of caspase-3 cleavage was captured using an automated imaging system as described in “Methods”. At treatment times around and beyond 24 h, RDV-induced caspase-3 cleavage was remarkably reduced by DEX in hepatocytes from liver donors HL#179 and #180 (Figure 2C). Similar protective effects were observed in HepG2 cells when RDV plus DEX was compared with RDV alone (Supplemental Fig. S3, http://links.lww.com/HC9/A171). Western blot analysis further revealed that RDV-induced cleavage of caspase-3 protein was decreased by DEX co-treatment (Figure 2D). Interestingly, RDV markedly increased the cleavage of caspase-8 (an effector caspase for extrinsic apoptosis) but only moderately fragmented caspase-9 (a marker for intrinsic apoptosis)^29^,^30^ (Figure 2E), suggesting that RDV stimulates HPH apoptosis mostly likely through extrinsic rather than intrinsic signaling pathways.

Formation of γ-H2AX, the phosphorylated H2AX on serine-139, is closely associated with increased DNA-double-strand breaks.^31^ We next evaluated γ-H2AX expression in HPH receiving RDV with and without DEX. As shown in Figures 2F and G, staining of γ-H2AX was significantly increased after hepatocytes (HL#178 and #184) were exposed to RDV at 10 and 20 µM for 48 h, while this elevation was repressed by DEX co-treatment. Collectively, these findings demonstrate that RDV induces hepatocyte death by promoting both apoptosis and necrosis pathways, and these damages could be efficiently protected by DEX co-treatment.

### RDV-induced hepatocytic leakage of ALT/AST and repression of albumin synthesis were partially reversed by DEX

**FIGURE 3:**
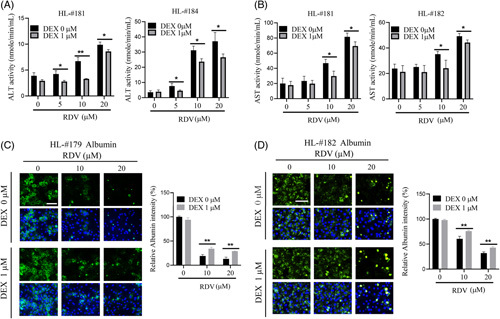
Dexamethasone alleviates remdesivir-induced hepatocyte malfunction. Sandwich-cultured human hepatocytes from liver donors (HL#181, #182, and #184) were treated with RDV at (0, 5, 10, and 20 µM) with or without DEX (1 µM) for 48 h. Cell culture medium was collected at the end of the treatments, and ALT (A) and AST (B) enzyme activities were examined as outlined in “Methods”. For albumin generation, hepatocytes from HL#179 (**C**) and #182 (D) were treated with RDV and DEX as indicated above for 48 h. Fluorescence immunostaining of albumin was visualized using the Nikon Elements microscope with a FITC channel. Relative fluorescence intensity was calculated. The scale bars represent 100 µm. Data are presented as mean±SD obtained from three separate experiments. Statistical significance was set at *p < 0.05; **p < 0.01; ***p < 0.001.

ALT and AST are hepatic enzymes that leak out into the general circulation when hepatocytes are injured. Albumin synthesis and secretion, on the other hand, represent a key function of hepatocytes. To test hepatocyte-specific toxic effects of RDV and the potential influence of DEX on these effects, HPH in 2D sandwich cultures was treated with RDV at indicated concentrations in the presence and absence of DEX. Our data demonstrated that ALT and AST were both elevated in the hepatocyte culture medium in a concentration-dependent manner following RDV treatment; importantly, DEX co-treatment significantly reduced RDV-stimulated ALT and AST leakage (Figure 3A and B). In another experiment, RDV treatment at 10 µM and 20 µM showed an 80–90% reduction of albumin synthesis compared with the vehicle control (0.1% DMSO) in HPH from liver donors HL#179 and #182, while the reduction was partially but significantly rescued by DEX co-treatment (Figure 3C and D). Overall, these findings demonstrate that hepatocyte function can be severely impaired by RDV administration *in vitro*, and DEX co-treatment attenuates RDV-induced functional damage in hepatocytes.

### COVID-19 patients receiving RDV and DEX were less likely to have serum AST/ALT elevation than patients treated with RDV alone

A total of 2002 adult patients (≥18 y of age) hospitalized with laboratory-confirmed SARS-CoV-2 infection treated with RDV or RDV/DEX from 4 clinical sites across the University of Maryland Medical System between May 1, 2020 and May 30, 2021 were included in this study. The majority (90.4%) of patients received RDV/DEX, while only 9.6% received RDV alone due to the change in the standard of care for the treatment of COVID-19 early in the study period. The baseline demographic information and clinical characteristics of COVID-19 patients are summarized in Table 1. In general, the distribution of patients between the 2 treatment groups differed in age (not significant), race, ethnicity, APR DRG SOI scores, and the number of concomitant medications with potential hepatotoxic side effects (Supplementary Tables S2-S4, http://links.lww.com/HC9/A95).

**TABLE 1:** Baseline demographic and clinical characteristics of hospitalized patients with COVID-19 between May 1, 2020 and May 30, 2021

	Pre-Match			1:5 Match		
Characteristics	RDV (n=192) (n, % column)	RDV+DEX (n=1810) (n, % column)	Total (n=2002) (n, %)	RDV (n=192) (n, % column)	RDV+DEX (n=845) (n, % column)	Total (n=1037) (n, %)
Sex						
Male	100 (52.1)	930 (51.4)	1030 (51.5)	100 (52.1)	425 (50.3)	525 (50.6)
Female	92 (47.9)	880 (48.6)	972 (48.5)	92 (47.9)	420 (49.7)	512 (49.4)
Age (y)	57.3±17.0	61.6±15.9	61.1±16.1	58.3±17.0	60.8±16.0	60.1±16.2
Race^a^						
White	60 (31.3)	854 (47.2)	914 (45.7)	60 (31.3)	300 (35.5)	360 (34.7)
Black or African American	82 (42.7)	740 (40.9)	822 (41.1)	82 (42.7)	398 (47.1)	480 (46.3)
Other	50 (25.0)	216 (11.9)	266 (13.2)	50 (25.0)	147 (17.4)	197 (19.0)
Ethnicity^a^						
Hispanic or Latino	45 (23.4)	132 (7.3)	177 (8.8)	45 (23.4)	76 (9.0)	121 (11.7)
Not Hispanic or Latino	146 (76.0)	1670 (92.3)	1816 (90.7)	146 (76.0)	762 (90.2)	908 (87.6)
Missing	1	8	9	1	7	8
APR DRG Score^a^						
<3	18 (9.4)	96 (5.3)	114 (5.7)	18 (9.4)	68 (8.1)	86 (8.3)
3	54 (28.1)	756 (41.8)	810 (40.5)	54 (28.1)	267 (31.6)	321 (31.0)
4	120 (62.5)	958 (52.9)	1078 (53.9)	120 (62.5)	510 (60.4)	630 (60.8)
Number of concomitants potentially hepatotoxic medications						
Metabolic Panel (U/L)	2.47±2.99	1.89±2.15	1.93±2.23	2.47±2.99	2.31±2.30	2.34±2.44
Baseline AST	47.9±26.8	47.4±24.9	47.5±25.1	47.9±26.8	47.7±25.2	47.7±25.5
Baseline ALT	34.9±25.1	33.7±22.7	33.8±23.0	34.9±25.1	33.9±22.8	34.1±23.2
Baseline total bilirubin (mg/dL)	0.63±0.45	0.65±0.45	0.65±0.45	0.63±0.45	0.64±0.40	0.64±0.41
Max AST	84.8±86.2	76.2±262.3	76.9±250.8	84.8±86.2	87.3±344.2	86.6±312.9
Max ALT	107.5±600.4	63.5±165.5	67.7±243.6	107.5±600.4	66.0±174.5	73.7±302.5
Max Total Bilirubin (mg/dL)	0.79±0.79	0.68±0.56	0.69±0.58	0.79±0.78	0.68±0.45	0.70±0.53

^a^ significantly associated with exposure, determined by χ^2^.

After a 1:5 propensity score matching analysis, a matched cohort of 1037 patients demonstrating no statistically significant differences in age, race, APR DRG SOI scores, or concomitant hepatotoxic medications was used for subsequent analysis. Differences in ethnicity were not included in the propensity score matching due to the relatively small sample size. As shown in Table 2, posttreatment serum AST and ALT elevation (≥ 3×ULN) was observed in 14 (7.3%) of the 192 patients treated with RDV alone compared with 27 (3.2%) of the 845 patients receiving RDV/DEX. Controlling for ethnicity and preexisting liver and renal disease (Table S2, http://links.lww.com/HC9/A95), patients receiving RDV in combination with DEX were half as likely to have AST and ALT ≥ 3×ULN compared with those treated with RDV alone (OR =0.44, 95% CI =0.22–0.92, *p* = 0.03).

**TABLE 2:** Association between exposure (Dexamethasone addition) and the Outcome (AST/ALT 3 X ULN) in COVID-19 patients receiving RDV treatment using 1:5 matched conditional logistic regression adjusted for age and ethnicity

AST and ALT	≥3 X ULN (n=41), n (%)	<3 X ULN (n=996), n (%)	Adjusted OR (95% CI)	Adjusted *p* a
Remdesivir alone (patient # and %)	14 (7.3)	178 (92.7)	REF	0.03
Remdesivir + Dexamethasone (patient # and %)	27 (3.2)	818 (96.8)	0.44 (0.22, 0.92)	

^a^ From conditional logistic regression model using propensity scores for race, preexisting liver disease, APR DRG SOI score, and medications with potential indication of hepatotoxicity adjusted for age and ethnicity.

## DISCUSSION

To the best of our knowledge, the present study is the first to demonstrate that co-administration of DEX significantly reduces RDV-induced cytotoxic responses in human hepatocytes, including alteration of cell viability, apoptosis, DNA damage, albumin production, and ALT/AST release. Further, the *in vitro* protective effects of DEX on RDV hepatotoxicity appear to be supported by a retrospective analysis of hospitalized COVID-19 patients.

To date, RDV has been used widely in hospitalized COVID-19 patients and is the first FDA-approved treatment for COVID-19, although several new drugs are authorized or in development.^32^ Clinical use of RDV however has been associated with transaminase elevations in both healthy volunteers and patients with COVID-19, which led to the recommendation of ALT/AST monitoring in patients before and during RDV treatment.^14^,^17^ In this report, marked cytotoxicity was observed in RDV-treated HPH in 2D sandwich culture and 3D spheroid format with CC_50_ values ranging from 4.95 to 22.66 µM, reflecting a consequence of both the culture conformation of hepatocytes and individual variation of liver donors. Notably, the lowest CC_50_ value of RDV at 4.95 µM is parallel to the reported serum C_max_ at 3.7 µM or 4.5 µM achieved at the FDA-labeled repeated dose of 100 mg or multidosing of 150 mg in humans, respectively.^24^,^25^ These findings agree with a recent report suggesting that human hepatocytes are highly sensitive to RDV in comparison with extrahepatic cells.^16^ Intriguingly, we found RDV is more cytotoxic in hepatic spheroids than 2D cultures, presumably due to the well-maintained metabolic function of hepatocytes in 3D spheroids, resulting in the higher intracellular conversion of RDV to the active and more toxic triphosphate metabolite (GS-443902).

The therapeutic application of DEX in COVID-19 patients relies predominantly on its antiinflammatory and immunomodulatory properties targeting the respiratory system. While the liver is generally not considered the therapeutic target for either RDV or DEX in this treatment scenario, DEX as a potent agonist of the glucocorticoid receptor plays crucial roles in liver functions.^22^,^33^ In exploring the interactions between RDV and DEX in the context of liver toxicity, we unexpectedly uncovered that DEX attenuates RDV-induced hepatotoxic responses in cultured HPH. Notably, the maximal liver protective effect was attained at 1 µM of DEX, a concentration that is approximate to the reported clinical C_max_ of DEX at 0.6 µM, after a single oral dose of 20 mg^34^ or at 0.5–0.8 µM for COVID-19 treatment after 6 mg DEX orally or intravenously daily for 7–10 days according to the World Health Organization guideline.^35^ It is well-known that excessive apoptosis and genomic DNA damage are characteristic adverse effects commonly associated with chemical-induced tissue injury. In this study, we found that in HPH RDV concentration-dependently increased caspase-3 cleavage and the phosphorylation of H2AX, 2 widely used biomarkers for the initiation of apoptosis and DNA damage,^27^,^28^ while these cellular damages were efficiently alleviated by DEX co-exposure. Further analysis of the upstream caspase signaling revealed that RDV markedly induced cleavage of caspase-8 but only moderately affected caspase-9, suggesting that RDV exposure most likely triggers the extrinsic not intrinsic apoptotic signaling pathway in HPH. In a liver specific context, RDV (10 and 20 µM) markedly increased hepatocyte release of ALT and AST, 2 aminotransferases predominantly expressed in hepatocytes and reliable markers of hepatocellular injury,^36^ while reducing the generation of albumin, a fundamental feature of healthy hepatocytes.^37^ Importantly, co-treatment with DEX partially but significantly reversed these biomarkers of liver damage induced by RDV. In contrast to our findings, a recent report suggested that DEX and RDV may have additive or synergistic toxic effects on HPH.^38^ However, the DEX concentrations (25–75 µM) used in that report were far beyond its pharmacological relevance.

It is worth noting that a low concentration of DEX (0.1 µM) is often contained in the “standard culture medium” of HPH to promote the cuboidal phenotypic architecture and facilitate liver-enriched gene expression of hepatocytes.^39^,^40^ This has raised the question of whether the protective effect of DEX on RDV was simply a result of maintaining the general well-being of hepatocytes. To address this query, additional cytotoxicity experiments were conducted in HepG2 cells, (Figure 1G and Fig. S3, http://links.lww.com/HC9/A171) a human hepatoma cell line that does not require DEX in culture. We found that DEX treatment efficiently reduced RDV-mediated cytotoxicity in HepG2 cells as well. Together, these findings suggest that the cyto-protective effects of DEX against RDV in liver cells cannot be simply explained as a consequence of improved *in vitro* culture condition of HPH. Nevertheless, the molecular mechanism(s) underlying these phenomena is largely unknown. CES1 is known as the key enzyme responsible for the hydrolysis of RDV, the initial step towards the formation of the active and toxic triphosphate metabolite.^41^ Indeed, Shen et al^42^ recently demonstrated that the overexpression of CES1 in stably transfected HEK293T cells significantly enhanced RDV-induced cytotoxicity, suggesting CES1-based hydrolysis is important for the therapeutic efficacy and safety of RDV. The effect of DEX on the hepatic expression of CES1, on the other hand, appears to be controversial. In rat primary hepatocytes, DEX markedly decreased the expression of rat CES1 at nanomolar concentrations, while its effect on human CES1 expression was uncertain, as shown in HPH cultures.^43^ Given the pleiotropic effects of DEX on the liver, fully deciphering the mechanism(s) by which DEX protects RDV-induced hepatotoxicity is beyond the scope of the current investigation, and future more comprehensive studies are warranted.

In our retrospective study, we observed that patients hospitalized with COVID-19 who received RDV/DEX were less likely to have an elevation of serum AST and ALT (≥ 3×ULN) when compared with patients treated with RDV alone. These findings are consistent with our *in vitro* cell-based experimental results supporting that DEX may attenuate RDV-associated transaminase elevations. While serum levels of ALT/AST are among the most extensively used biomarkers in predicting hepatic lesions, interpretation of the data could be affected by numerous co-existing factors and limitations.^36^,^44^ Our study was limited by an imbalance in the number of patients who received RDV monotherapy. Treatment guidelines for the management of COVID-19 changed to recommend the addition of DEX as the standard of care in patients requiring supplemental oxygen early in the study period. In addition, transient liver injury has been described as a complication of COVID-19 itself in patients not treated with RDV or DEX.^45^ Moreover, *in vitro* cell-based experiments are associated with their own inherent disadvantages in comparison with animal models. However, the use of HPH does address species-specific concerns that cannot be answered by animal studies. Finally, due to the retrospective nature of the study and limitations in the available data, we were limited in the potential confounders, which we could control for in our analysis. Thus, despite the positive correlation of our *in vitro* results with the real-world data from a limited hospital network, future larger prospective studies are required to determine the effect of DEX and RDV transaminitis.

In summary, the current study describes a potential protective effect of DEX against RDV-induced liver toxicity. Through well-controlled *in vitro* experiments, we demonstrated that co-treatment with DEX mitigates RDV-induced apoptosis, DNA damage, and the hepatic release of ALT and AST. Real-world data revealed that COVID-19 patients receiving RDV plus DEX appear to be less likely to have elevated ALT and AST compared with those treated with RDV alone. Given their frequent concomitant administration in hospitalized patients with severe COVID-19, DEX may attenuate the risk of transaminase elevations with RDV.

## Supplementary Material

## References

[1] Mishra SK Tripathi T. One year update on the COVID-19 pandemic: Where are we now? Acta Trop. 2021;214:105778. 33253656 10.1016/j.actatropica.2020.105778 PMC7695590

[2] Verity R Okell LC Dorigatti I Winskill P Whittaker C Imai N. Estimates of the severity of coronavirus disease 2019: a model-based analysis. Lancet Infect Dis. 2020;20:669–677. 32240634 10.1016/S1473-3099(20)30243-7 PMC7158570

[3] Zhou F Yu T Du R Fan G Liu Y Liu Z. Clinical course and risk factors for mortality of adult inpatients with COVID-19 in Wuhan, China: a retrospective cohort study. Lancet. 2020;395:1054–1062. 32171076 10.1016/S0140-6736(20)30566-3 PMC7270627

[4] Porter DP Weidner JM Gomba L Bannister R Blair C Jordan R. Remdesivir (GS-5734) is efficacious in cynomolgus macaques infected with marburg virus. J Infect Dis. 2020;222:1894–1901. 32479636 10.1093/infdis/jiaa290

[5] Warren TK Jordan R Lo MK Ray AS Mackman RL Soloveva V. Therapeutic efficacy of the small molecule GS-5734 against Ebola virus in rhesus monkeys. Nature. 2016;531:381–385. 26934220 10.1038/nature17180 PMC5551389

[6] de Wit E Feldmann F Cronin J Jordan R Okumura A Thomas T. Prophylactic and therapeutic remdesivir (GS-5734) treatment in the rhesus macaque model of MERS-CoV infection. Proc Natl Acad Sci U S A. 2020;117:6771–6776. 32054787 10.1073/pnas.1922083117 PMC7104368

[7] Wang M Cao R Zhang L Yang X Liu J Xu M. Remdesivir and chloroquine effectively inhibit the recently emerged novel coronavirus (2019-nCoV) in vitro. Cell Res. 2020;30:269–271. 32020029 10.1038/s41422-020-0282-0 PMC7054408

[8] Gordon CJ Tchesnokov EP Woolner E Perry JK Feng JY Porter DP. Remdesivir is a direct-acting antiviral that inhibits RNA-dependent RNA polymerase from severe acute respiratory syndrome coronavirus 2 with high potency. J Biol Chem. 2020;295:6785–6797. 32284326 10.1074/jbc.RA120.013679 PMC7242698

[9] Runfeng L Yunlong H Jicheng H Weiqi P Qinhai M Yongxia S. Lianhuaqingwen exerts anti-viral and anti-inflammatory activity against novel coronavirus (SARS-CoV-2). Pharmacol Res. 2020;156:104761. 32205232 10.1016/j.phrs.2020.104761 PMC7102548

[10] Beigel JH Tomashek KM Dodd LE Mehta AK Zingman BS Kalil AC. Remdesivir for the Treatment of Covid-19 - Final Report. N Engl J Med. 2020;383:1813–1826. 32445440 10.1056/NEJMoa2007764 PMC7262788

[11] Mozaffari E Chandak A Zhang Z Liang S Thrun M Gottlieb RL. Remdesivir treatment in hospitalized patients with COVID-19: a comparative analysis of in-hospital all-cause mortality in a large multi-center observational cohort. Clin Infect Dis. 2021;75:e450–e458. 10.1093/cid/ciab875 PMC9402660 34596223

[12] Gottlieb RL Vaca CE Paredes R Mera J Webb BJ Perez G. Early Remdesivir to Prevent Progression to Severe COVID-19 in Outpatients. N Engl J Med. 2021;386(4):305–315. 34937145 10.1056/NEJMoa2116846 PMC8757570

[13] Consortium WHOST. Remdesivir and three other drugs for hospitalised patients with COVID-19: final results of the WHO Solidarity randomised trial and updated meta-analyses. Lancet. 2022;399:1941–1953. 35512728 10.1016/S0140-6736(22)00519-0 PMC9060606

[14] Zampino R Mele F Florio LL Bertolino L Andini R Galdo M. Liver injury in remdesivir-treated COVID-19 patients. Hepatol Int. 2020;14:881–883. 32725454 10.1007/s12072-020-10077-3 PMC7386240

[15] Aleem A Mahadevaiah G Shariff N Kothadia JP. Hepatic manifestations of COVID-19 and effect of remdesivir on liver function in patients with COVID-19 illness. Proc (Bayl Univ Med Cent). 2021;34:473–477. 34219928 10.1080/08998280.2021.1885289 PMC8224190

[16] Xu Y Barauskas O Kim C Babusis D Murakami E Kornyeyev D. Off-target in vitro profiling demonstrates that remdesivir is a highly selective antiviral agent. Antimicrob Agents Chemother. 2021;65:e02237–20. 33229429 10.1128/AAC.02237-20 PMC7849018

[17] Carothers C Birrer K Vo M. Acetylcysteine for the treatment of suspected remdesivir-associated acute liver failure in COVID-19: A Case Series. Pharmacotherapy. 2020;40:1166–1171. 33006138 10.1002/phar.2464 PMC7537093

[18] Group RC Horby P Lim WS Emberson JR Mafham M Bell JL. Dexamethasone in hospitalized patients with COVID-19. N Engl J Med. 2021;384:693–704. 32678530 10.1056/NEJMoa2021436 PMC7383595

[19] Tomazini BM Maia IS Cavalcanti AB Berwanger O Rosa RG Veiga VC. Effect of dexamethasone on days alive and ventilator-free in patients with moderate or severe acute respiratory distress syndrome and COVID-19: The CoDEX randomized clinical trial. JAMA. 2020;324:1307–1316. 32876695 10.1001/jama.2020.17021 PMC7489411

[20] Benfield T Bodilsen J Brieghel C Harboe ZB Helleberg M Holm C. Improved survival among hospitalized patients with COVID-19 treated with remdesivir and dexamethasone. A nationwide population-based cohort study. Clin Infect Dis. 2021;73:2031–2036. 34111274 10.1093/cid/ciab536 PMC8344480

[21] NIH. Coronavirus Disease 2019 (COVID-19) Treatment Guidelines. In; 2022. 34003615

[22] Chou JY Wan YJ Sakiyama T. Regulation of rat liver maturation in vitro by glucocorticoids. Mol Cell Biol. 1988;8:203–209. 2447484 10.1128/mcb.8.1.203-209.1988 PMC363102

[23] Li L Welch MA Li Z Mackowiak B Heyward S Swaan PW. Mechanistic insights of phenobarbital-mediated activation of human but not mouse pregnane X receptor. Mol Pharmacol. 2019;96:345–354. 31436536 10.1124/mol.119.116616 PMC6701513

[24] Humeniuk R Mathias A Cao H Osinusi A Shen G Chng E. Safety, tolerability, and pharmacokinetics of remdesivir, an antiviral for treatment of COVID-19, in healthy subjects. Clin Transl Sci. 2020;13:896–906. 32589775 10.1111/cts.12840 PMC7361781

[25] FDA. VEKLURY (remdesivir) package insert. In; 2022.

[26] FDA. CDER. Guidance for Industry Drug-Induced Liver Injury: Premarketing Clinical Evaluation. FDA document, CDER, Silver Spring, MD. FDA document, CDER, Silver Spring, MD. 2009.

[27] Valdiglesias V Giunta S Fenech M Neri M Bonassi S. gammaH2AX as a marker of DNA double strand breaks and genomic instability in human population studies. Mutat Res. 2013;753:24–40. 23416207 10.1016/j.mrrev.2013.02.001

[28] Abu-Qare AW Abou-Donia MB. Biomarkers of apoptosis: release of cytochrome c, activation of caspase-3, induction of 8-hydroxy-2'-deoxyguanosine, increased 3-nitrotyrosine, and alteration of p53 gene. J Toxicol Environ Health B Crit Rev. 2001;4:313–332. 11503418 10.1080/109374001301419737

[29] Tummers B Green DR. Caspase-8: regulating life and death. Immunol Rev. 2017;277:76–89. 28462525 10.1111/imr.12541 PMC5417704

[30] Johnson CR Jarvis WD. Caspase-9 regulation: an update. Apoptosis. 2004;9:423–427. 15192324 10.1023/B:APPT.0000031457.90890.13

[31] Mah LJ El-Osta A Karagiannis TC. gammaH2AX: a sensitive molecular marker of DNA damage and repair. Leukemia. 2010;24:679–686. 20130602 10.1038/leu.2010.6

[32] Wen W Chen C Tang J Wang C Zhou M Cheng Y. Efficacy and safety of three new oral antiviral treatment (molnupiravir, fluvoxamine and Paxlovid) for COVID-19a meta-analysis. Ann Med. 2022;54:516–523. 35118917 10.1080/07853890.2022.2034936 PMC8820829

[33] Sidhu JS Liu F Omiecinski CJ. Phenobarbital responsiveness as a uniquely sensitive indicator of hepatocyte differentiation status: requirement of dexamethasone and extracellular matrix in establishing the functional integrity of cultured primary rat hepatocytes. Exp Cell Res. 2004;292:252–264. 14697333 10.1016/j.yexcr.2003.09.001

[34] FDA. HEMADY (dexamethasone tablets) package insert. In; 2019.

[35] Abouir K Gosselin P Guerrier S Daali Y Desmeules J Grosgurin O. Dexamethasone exposure in normal-weight and obese hospitalized COVID-19 patients: An observational exploratory trial. Clin Transl Sci. 2022;15:1796–1804. 35706350 10.1111/cts.13297 PMC9283739

[36] McGill MR. The past and present of serum aminotransferases and the future of liver injury biomarkers. EXCLI J. 2016;15:817–828. 28337112 10.17179/excli2016-800 PMC5318690

[37] Spinella R Sawhney R Jalan R. Albumin in chronic liver disease: structure, functions and therapeutic implications. Hepatol Int. 2016;10:124–132. 26420218 10.1007/s12072-015-9665-6

[38] Khalatbari A Aghazadeh Z Ji C. Adverse effects of Anti-Covid-19 drug candidates and alcohol on cellular stress responses of hepatocytes. Hepatol Commun. 2021;6:1262–1277. 10.1002/hep4.1887 PMC9134820 34910385

[39] Oh HY Namkoong S Lee SJ Por E Kim CK Billiar TR. Dexamethasone protects primary cultured hepatocytes from death receptor-mediated apoptosis by upregulation of cFLIP. Cell Death Differ. 2006;13:512–523. 16167066 10.1038/sj.cdd.4401771

[40] Bailly-Maitre B de Sousa G Boulukos K Gugenheim J Rahmani R. Dexamethasone inhibits spontaneous apoptosis in primary cultures of human and rat hepatocytes via Bcl-2 and Bcl-xL induction. Cell Death Differ. 2001;8:279–288. 11319611 10.1038/sj.cdd.4400815

[41] Li R Liclican A Xu Y Pitts J Niu C Zhang J. Key metabolic enzymes involved in remdesivir activation in human lung cells. Antimicrob Agents Chemother. 2021;65:e0060221. 34125594 10.1128/AAC.00602-21 PMC8370248

[42] Shen Y Eades W Yan B. The COVID-19 medicine remdesivir is therapeutically activated by carboxylesterase-1, and excessive hydrolysis increases cytotoxicity. Hepatol Commun. 2021;5:1622–1623. 34510834 10.1002/hep4.1736 PMC8250894

[43] Zhu W Song L Zhang H Matoney L LeCluyse E Yan B. Dexamethasone differentially regulates expression of carboxylesterase genes in humans and rats. Drug Metab Dispos. 2000;28:186–191. 10640517

[44] Senior JR. Alanine aminotransferase: a clinical and regulatory tool for detecting liver injury-past, present, and future. Clin Pharmacol Ther. 2012;92:332–339. 22871997 10.1038/clpt.2012.108

[45] Kunutsor SK Laukkanen JA. Hepatic manifestations and complications of COVID-19: A systematic review and meta-analysis. J Infect. 2020;81:e72–e74. 10.1016/j.jinf.2020.06.043 PMC7306105 32579984

