# Supplementary material for: Dexamethasone mitigates remdesivir-induced liver toxicity in human primary hepatocytes and COVID-19 patients
Source: Hepatol Commun. 2023 Feb 20;7(3):e0034. doi: 10.1097/HC9.0000000000000034 (PMC9949788; doi:10.1097/HC9.0000000000000034)
Supplement: Supplementary file 1 [file hc9-7-e0034-s001.docx]

**Hepatotoxicity of immune checkpoint inhibitors: A Bayesian network meta-analysis**

**Supplemental materials**

[01 eFigure 1 Literature screening and selection flow chart. 3](#_Toc109111747)

[02 eFigure 2. Bias risk map from the Cochrane systematic evaluation method to evaluate the quality of the included randomized control trials. 4](#_Toc109111748)

[03 eFigure 3 Funnel plot of Hepatotoxicity 5](#_Toc109111749)

[eFigure 3.1 Funnel plot of treatment-related hepatotoxicity 5](#_Toc109111750)

[eFigure 3.2 Funnel plot of immune-mediated hepatotoxicity 6](#_Toc109111751)

[eFigure 3.3 Funnel plot of Fatal Liver adverse events 8](#_Toc109111752)

[04 eFigure 4. Forest plot of the hepatotoxicity-induced by ICIs. 9](#_Toc109111753)

[eFigure 4.1 Forest plot of the Treatment-related hepatotoxicity by ICIs. 9](#_Toc109111754)

[eFigure 4.2 Forest plot of the Immune-mediated hepatotoxicity by ICIs. 17](#_Toc109111755)

[05 eFigure 5. Network plot 21](#_Toc109111756)

[06 eFigure 6. Rankings of SUCRA for the risk of hepatotoxicity 23](#_Toc109111757)

[eFigure 6.1 The distribution of Treatment-related Hepatotoxicity SUCRA values 23](#_Toc109111758)

[eFigure 6.2 The distribution of Immune-mediated Hepatotoxicity SUCRA values. 24](#_Toc109111759)

[07 eFigure 7. Regimen ranking of fatal liver AEs 25](#_Toc109111760)

[08 eFigure 8. Subgroup analysis by cancer type 26](#_Toc109111761)

[eFigure 8.1 Network plots of Treatment-related ALT by cancer type 26](#_Toc109111762)

[09 eFigure 9. Subgroup analysis of the risk of hepatotoxicity by dose 26](#_Toc109111763)

[eFigure 9.1 Subgroup analysis of the risk of hepatotoxicity by Nivolumab dose 26](#_Toc109111764)

[eFigure 9.2 Subgroup analysis of the risk of hepatotoxicity by Atezolizumab dose 27](#_Toc109111765)

[eFigure 9.3 Subgroup analysis of the risk of hepatotoxicity by Atezolizumab dose 27](#_Toc109111766)

[eFigure 9.4 Subgroup analysis of the risk of hepatotoxicity by Ipilimumab dose 28](#_Toc109111767)

[eFigure 9.5 Subgroup analysis of the risk of hepatotoxicity by Pembrolizumab dose 28](#_Toc109111768)

[10 eTable 1. Literature search strategy 29](#_Toc109111769)

[11 eTable 2. Main characteristics of the trials included in this meta-analysis 35](#_Toc109111770)

[12 eTable 3. Network estimates of treatment comparisons for Treatment-related all-grade and grade 3-5 hepatotoxicity 53](#_Toc109111771)

[eTable 3.1 Network estimates of treatment comparisons for Treatment-related all-grade and grade 3-5 ALT 53](#_Toc109111772)

[eTable 3.2 Network estimates of treatment comparisons for Treatment-related all-grade and grade 3-5 AST 53](#_Toc109111773)

[eTable 3.3 Network estimates of treatment comparisons for Treatment-related all-grade and grade 3-5 ALP 54](#_Toc109111774)

[eTable 3.4 Network estimates of treatment comparisons for Treatment-related all-grade and grade 3-5 GGT 55](#_Toc109111775)

[eTable 3.5 Network estimates of treatment comparisons for Treatment-related all-grade and grade 3-5 Bilirubin 56](#_Toc109111776)

[13 eTable 4. Network estimates of treatment comparisons for Immune-mediated all-grade and grade 3-5 hepatotoxicity 57](#_Toc109111777)

[eTable 4.1 Network estimates of treatment comparisons for Immune-mediated all-grade and grade 3-5 ALT 57](#_Toc109111778)

[eTable 4.2 Network estimates of treatment comparisons for Immune-mediated all-grade and grade 3-5 AST 58](#_Toc109111779)

[eTable 4.3 Network estimates of treatment comparisons for Immune-mediated all-grade and grade 3-5 GGT 59](#_Toc109111780)

[eTable 4.4 Network estimates of treatment comparisons for Immune-mediated all-grade and grade 3-5 ALP 60](#_Toc109111781)

[eTable 4.5 Network estimates of treatment comparisons for Immune-mediated all-grade and grade 3-5 Bilirubin 61](#_Toc109111782)

[14 eTable 5 Network estimates of treatment comparisons for Fatal liver adverse events. 63](#_Toc109111783)

[15 eTable 6. The ranking of treatment regimen on Treatment-related hepatotoxicity by cancer type 64](#_Toc109111784)

[eTable 6.1 Network estimates of treatment comparisons for Treatment-related all-grade ALT and grade 3-5 ALT on respiratory system 64](#_Toc109111785)

[eTable 6.2 Network estimates of treatment comparisons for Treatment-related all-grade ALT and grade 3-5 ALT on head and neck system cancer. 64](#_Toc109111786)

[eTable 6.3 Network estimates of treatment comparisons for Treatment-related all-grade ALT and grade 3-5 ALT on skin cancer. 65](#_Toc109111787)

[eTable 6.4 Network estimates of treatment comparisons for Treatment-related all-grade ALT and grade 3-5 ALT on urogenital system cancer. 66](#_Toc109111788)

[eTable 6.5 Network estimates of treatment comparisons for Treatment-related all-grade ALT and grade 3-5 ALT on digest system cancer. 67](#_Toc109111789)

[16 eTable 7. The results of random consistency evaluation 68](#_Toc109111790)

[17 eTable 8. The list of the targeted therapy drug in main cancer. 70](#_Toc109111791)

# 01 eFigure 1 Literature screening and selection flow chart.


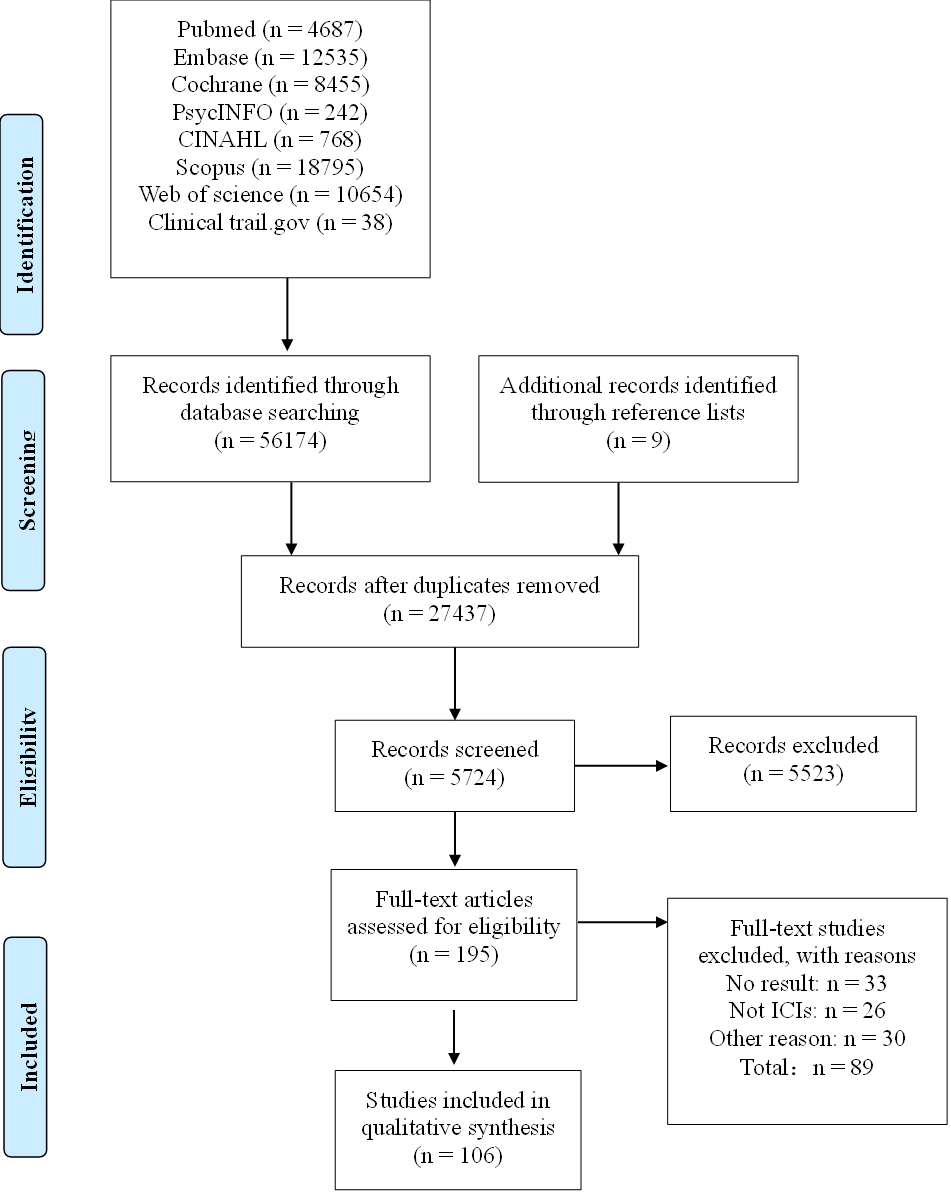


# 02 eFigure 2. Bias risk map from the Cochrane systematic evaluation method to evaluate the quality of the included randomized control trials.


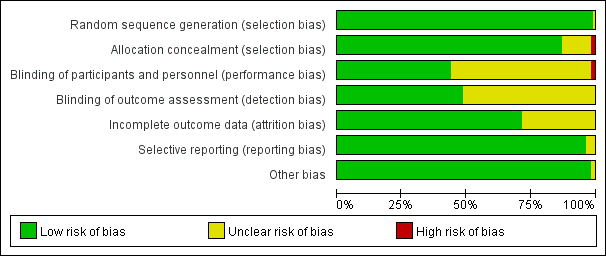

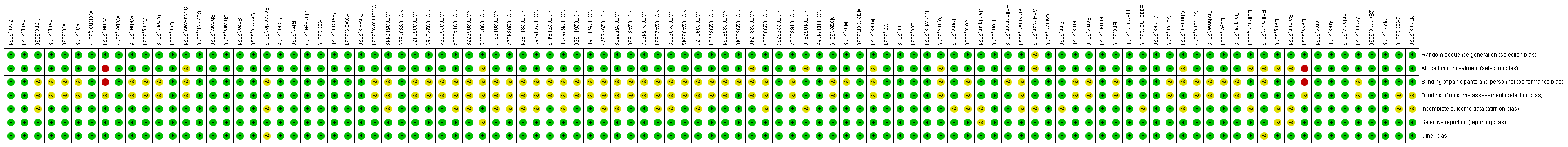


# 03 eFigure 3 Funnel plot of Hepatotoxicity

eFigure 3.1 Funnel plot of treatment-related hepatotoxicity

(A：All-grade ALT；B：Grade 3-5 ALT；C：All-grade AST；D：Grade 3-5AST；E：All-grade ALP；F：Grade 3-5ALP；G：All-grade GGT；H：Grade 3-5GGT；I：All-grade Bilirubin；J：Grade 3-5 Bilirubin)


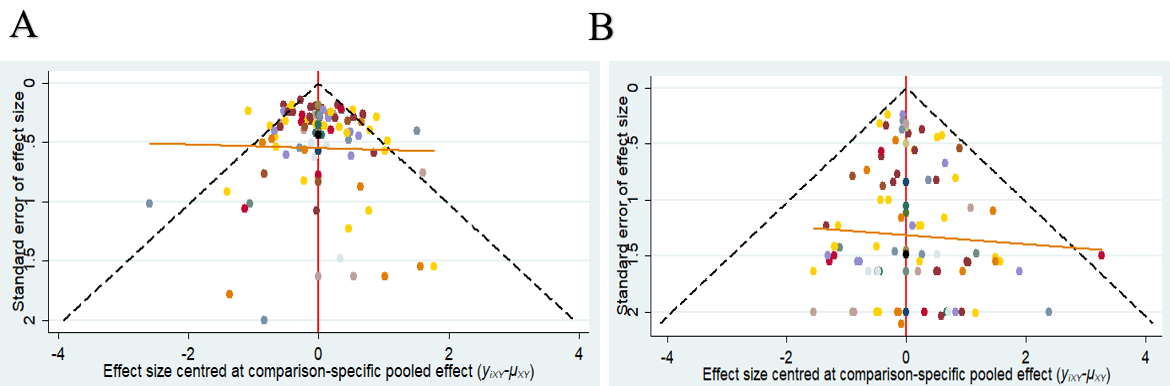


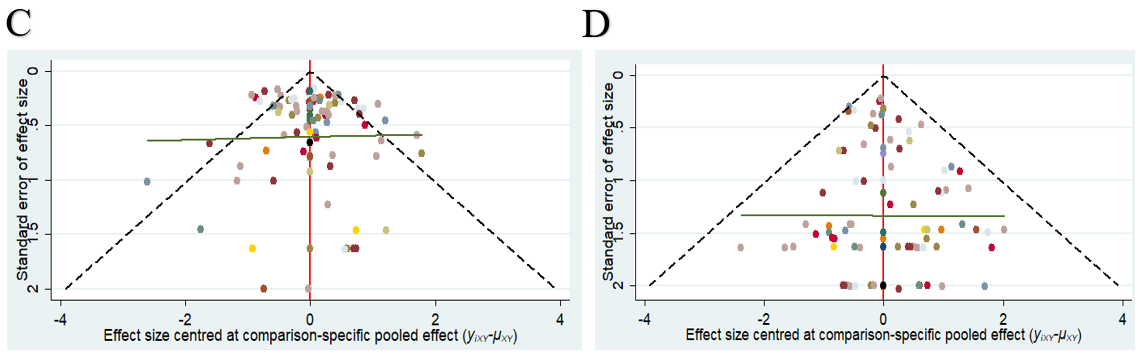


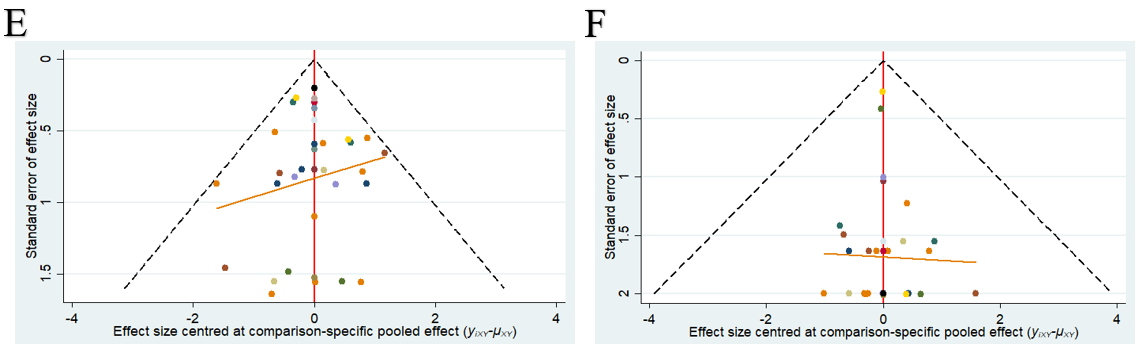


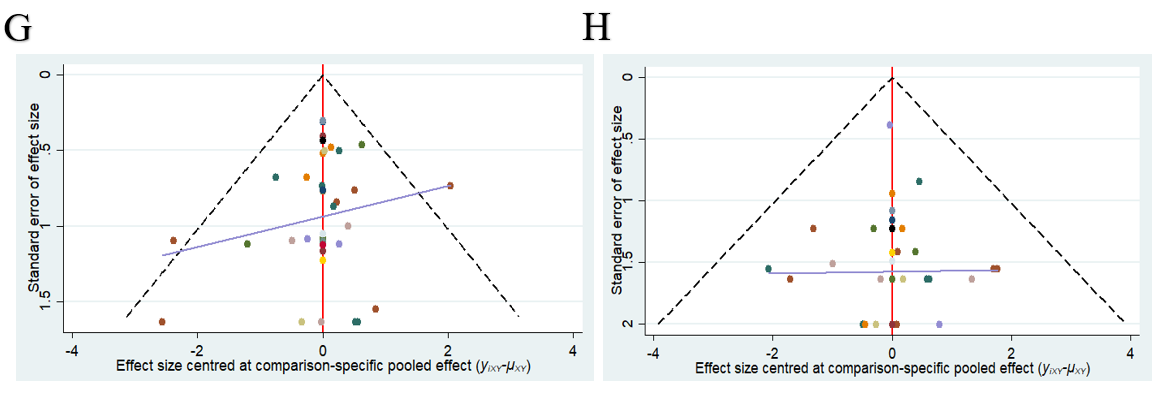


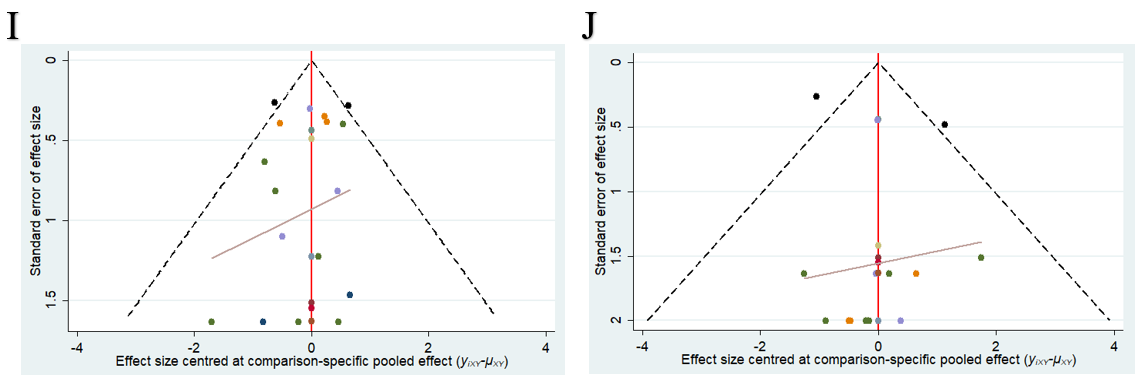


eFigure 3.2 Funnel plot of immune-mediated hepatotoxicity

(A：All-grade ALT；B：Grade 3-5 ALT；C：All-grade AST；D：Grade 3-5AST；E：All-grade ALP；F：Grade 3-5ALP；G：All-grade GGT；H：Grade 3-5GGT；I：All-grade Bilirubin；J：Grade 3-5 Bilirubin）


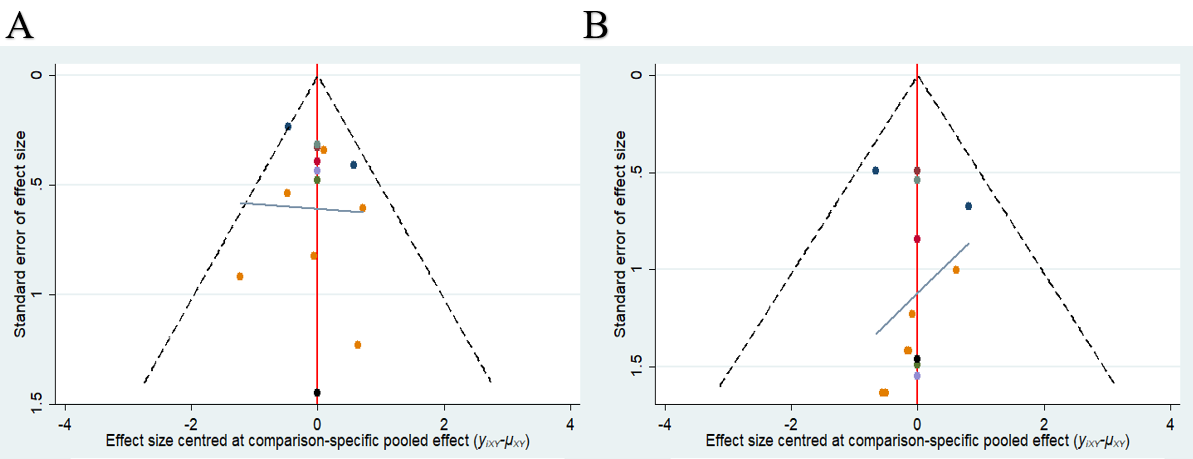

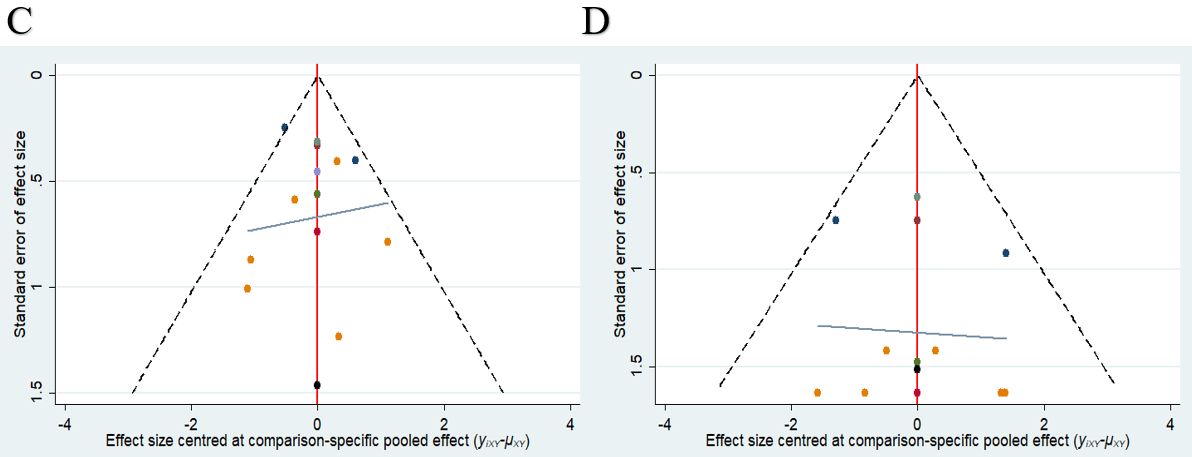


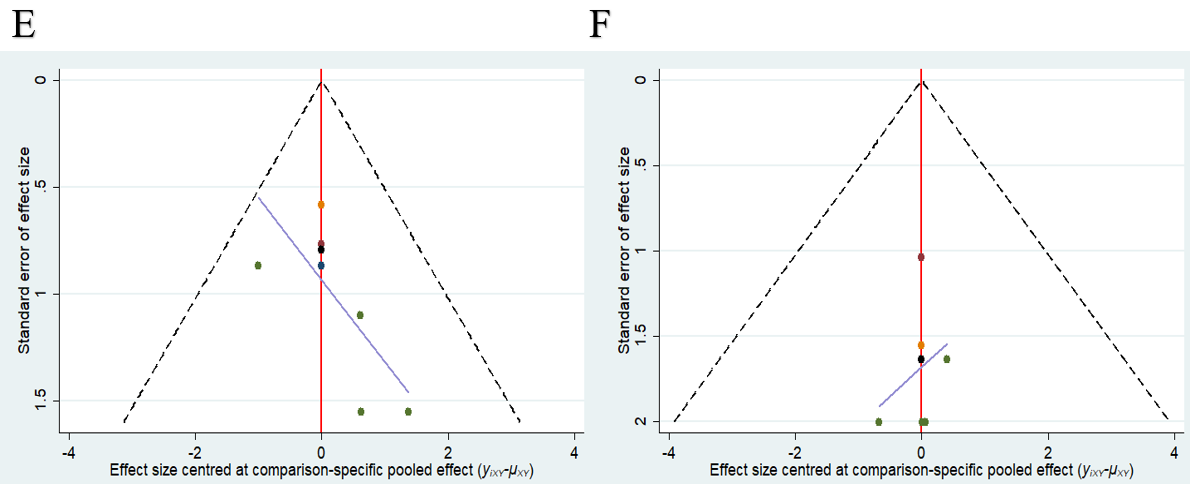

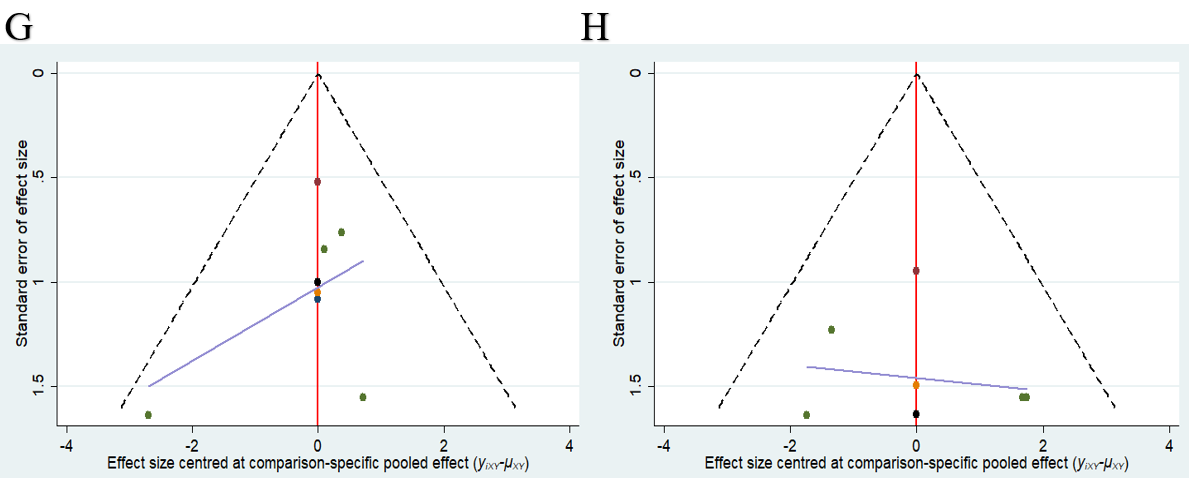


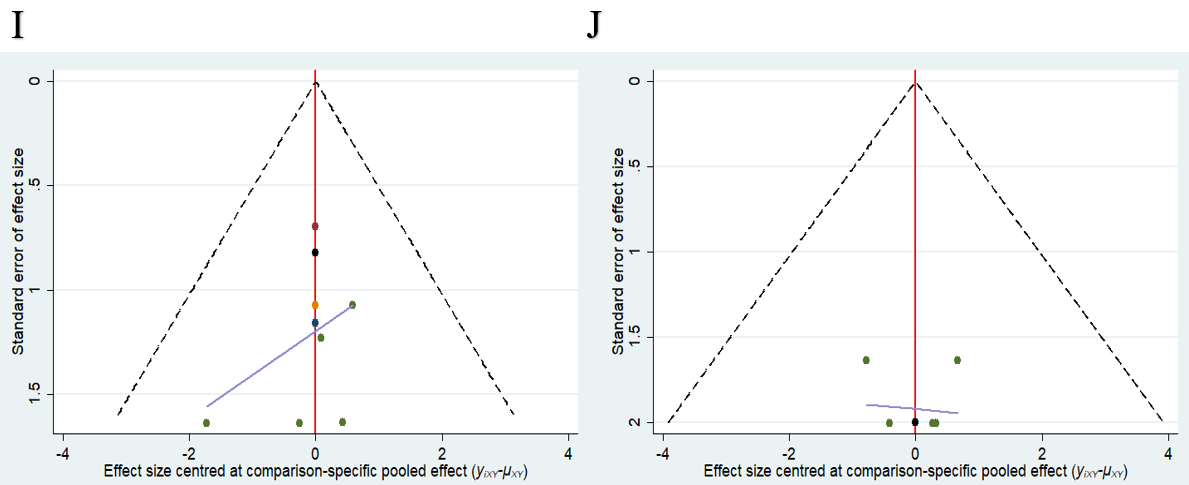


## eFigure 3.3 Funnel plot of Fatal Liver adverse events


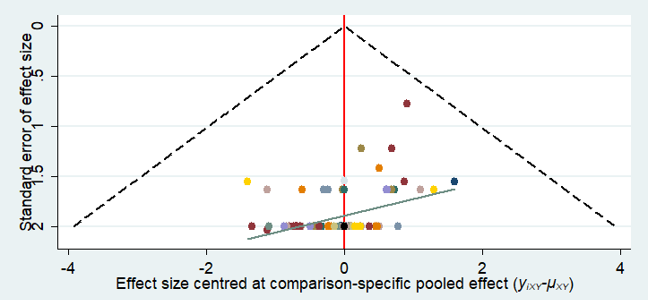


# 04 eFigure 4. **Forest plot of the hepatotoxicity-induced by ICIs.**

## eFigure 4.1 Forest plot of the Treatment-related hepatotoxicity by ICIs.

(A：All-grade ALT；B：Grade 3-5 ALT；C：All-grade AST；D：Grade 3-5AST；E：All-grade ALP；F：Grade 3-5ALP；G：All-grade GGT；H：Grade 3-5GGT；I：All-grade Bilirubin；J：Grade 3-5 Bilirubin）

**A
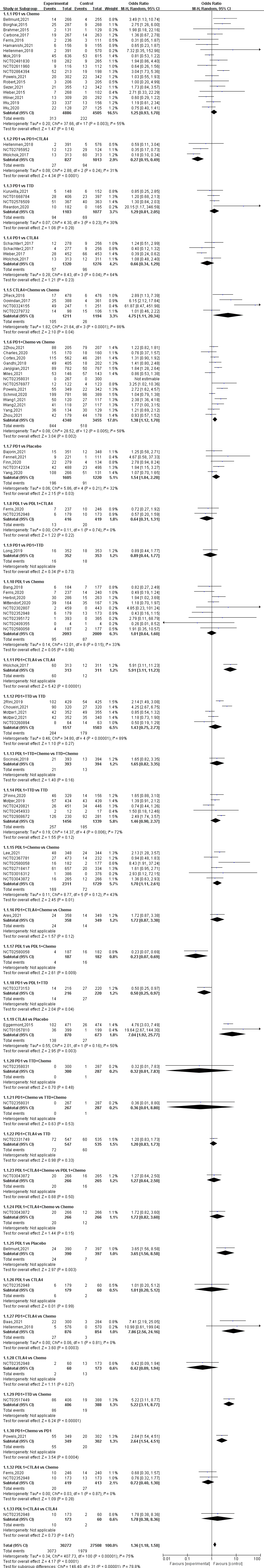
 B
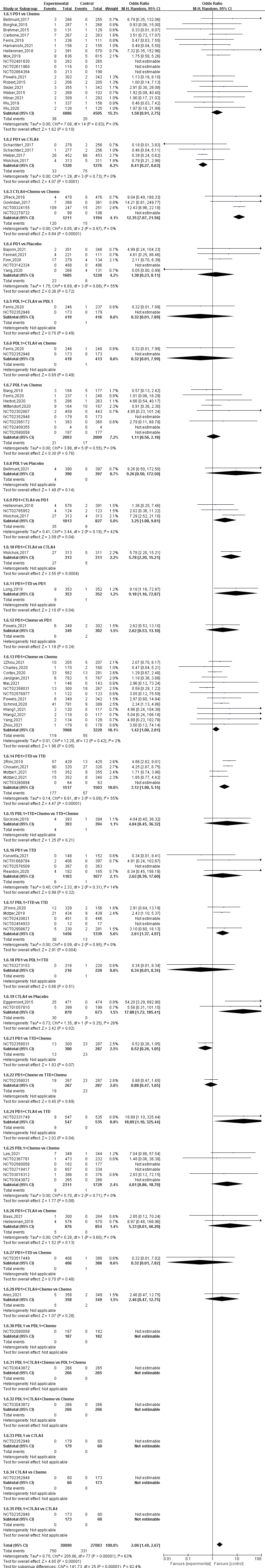
 C
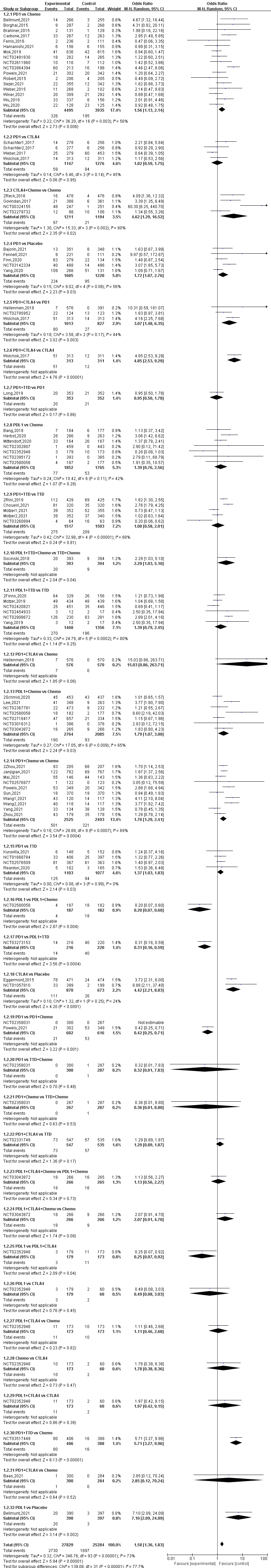
**

**D
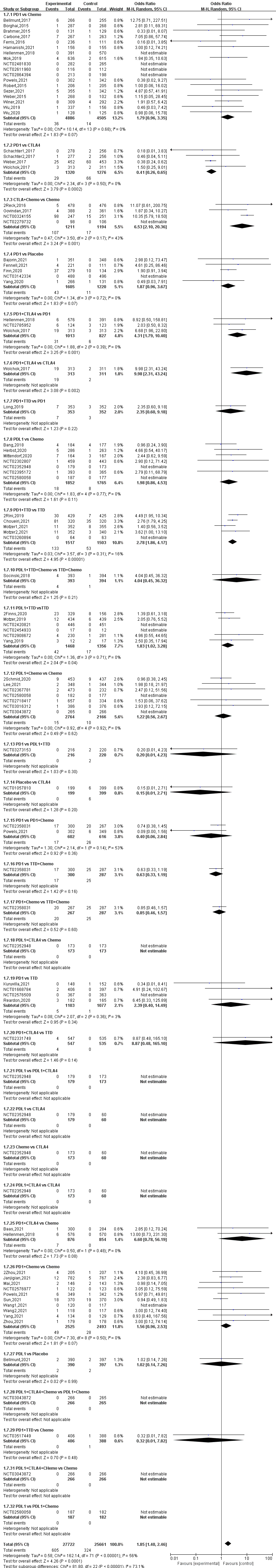
E
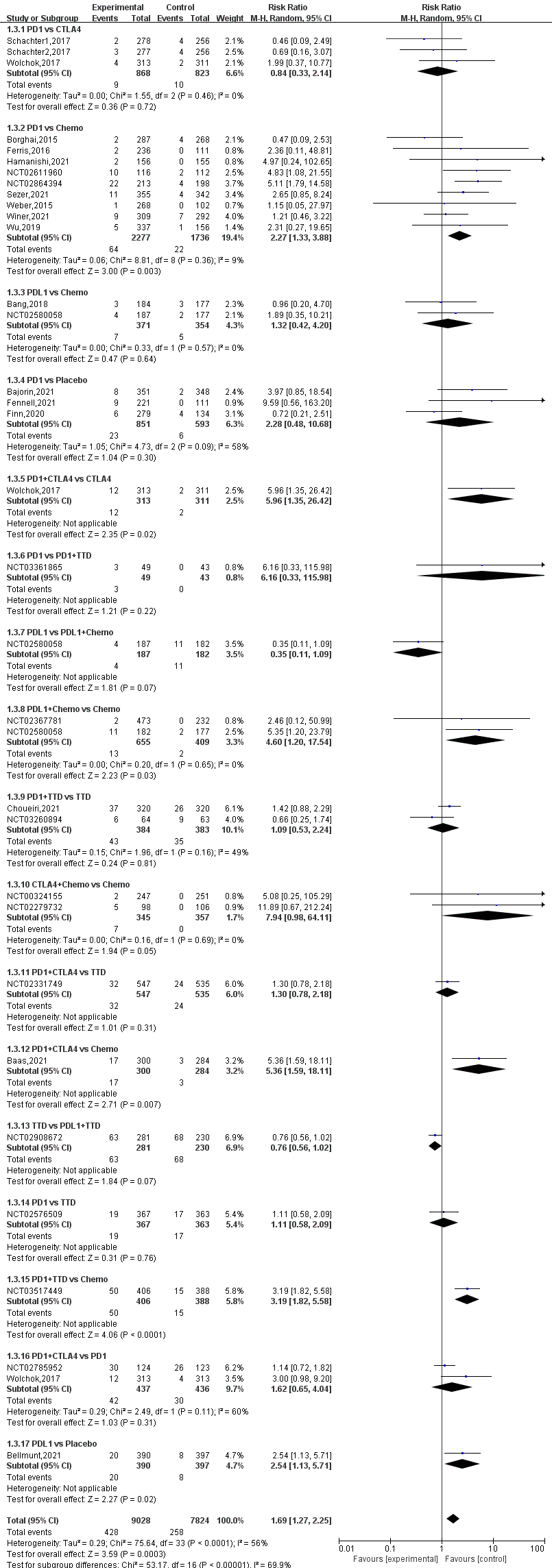
**

**F
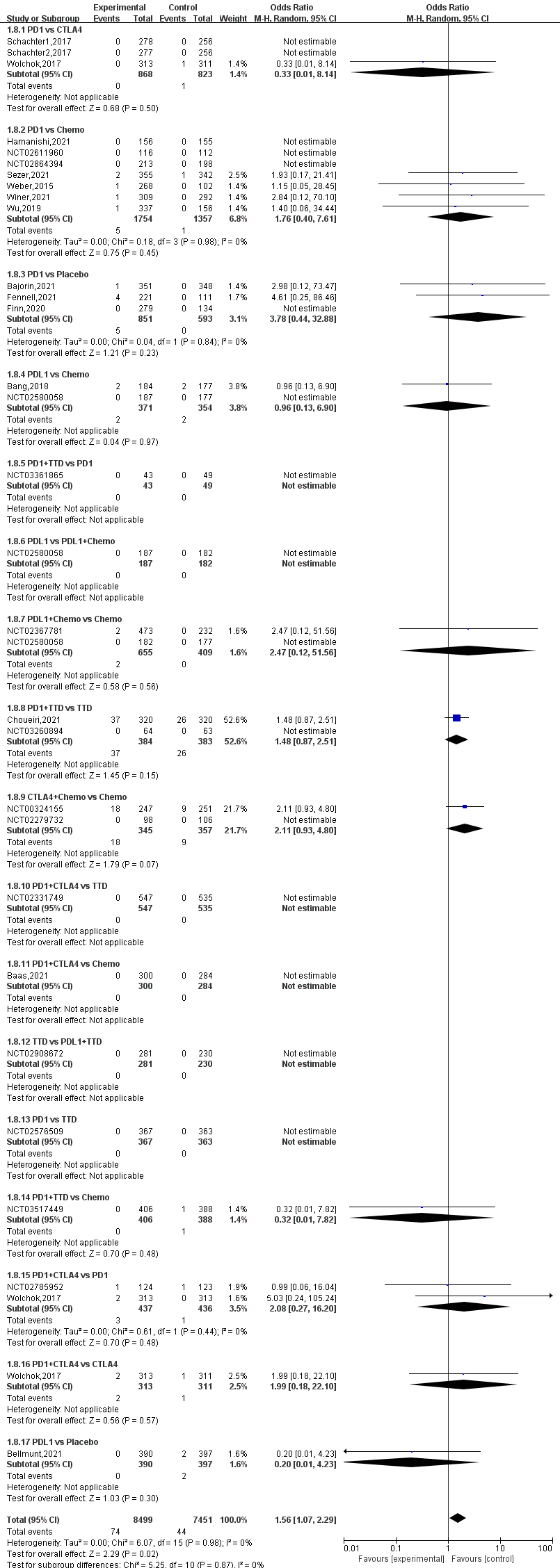
**

**G
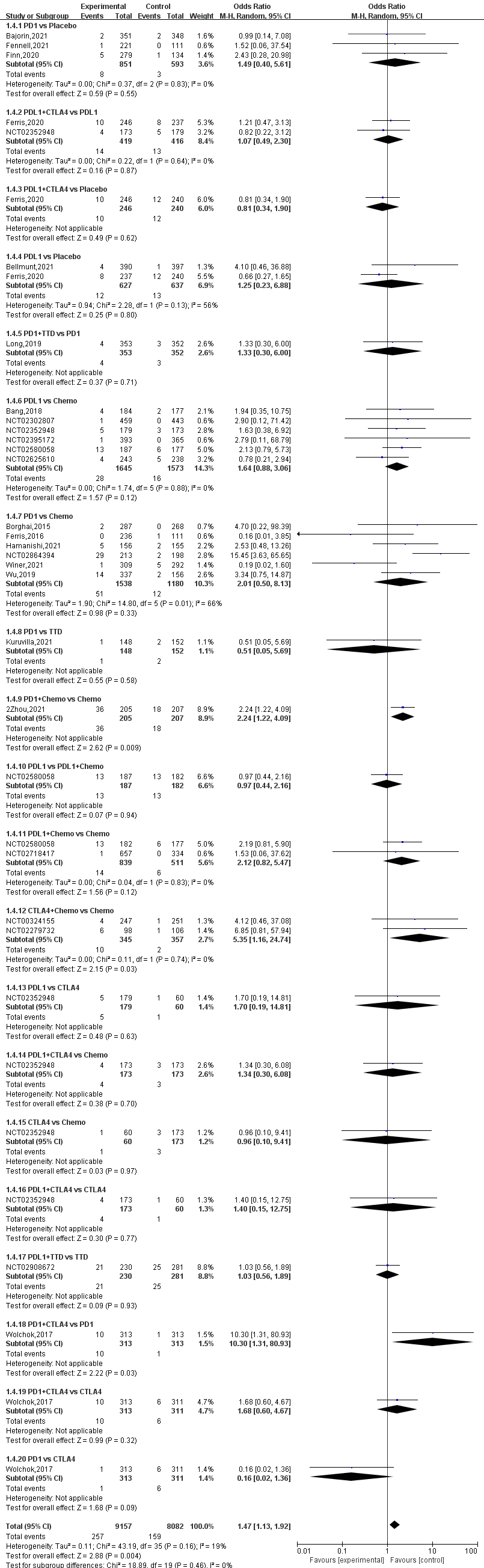
**

**H
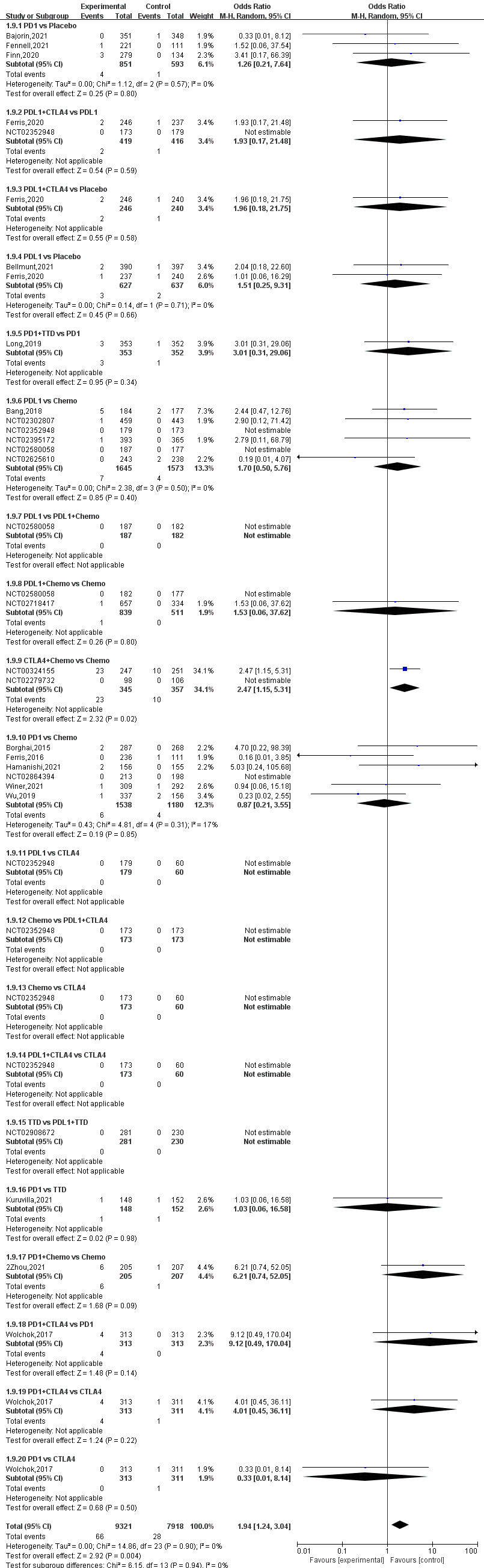
**

**I
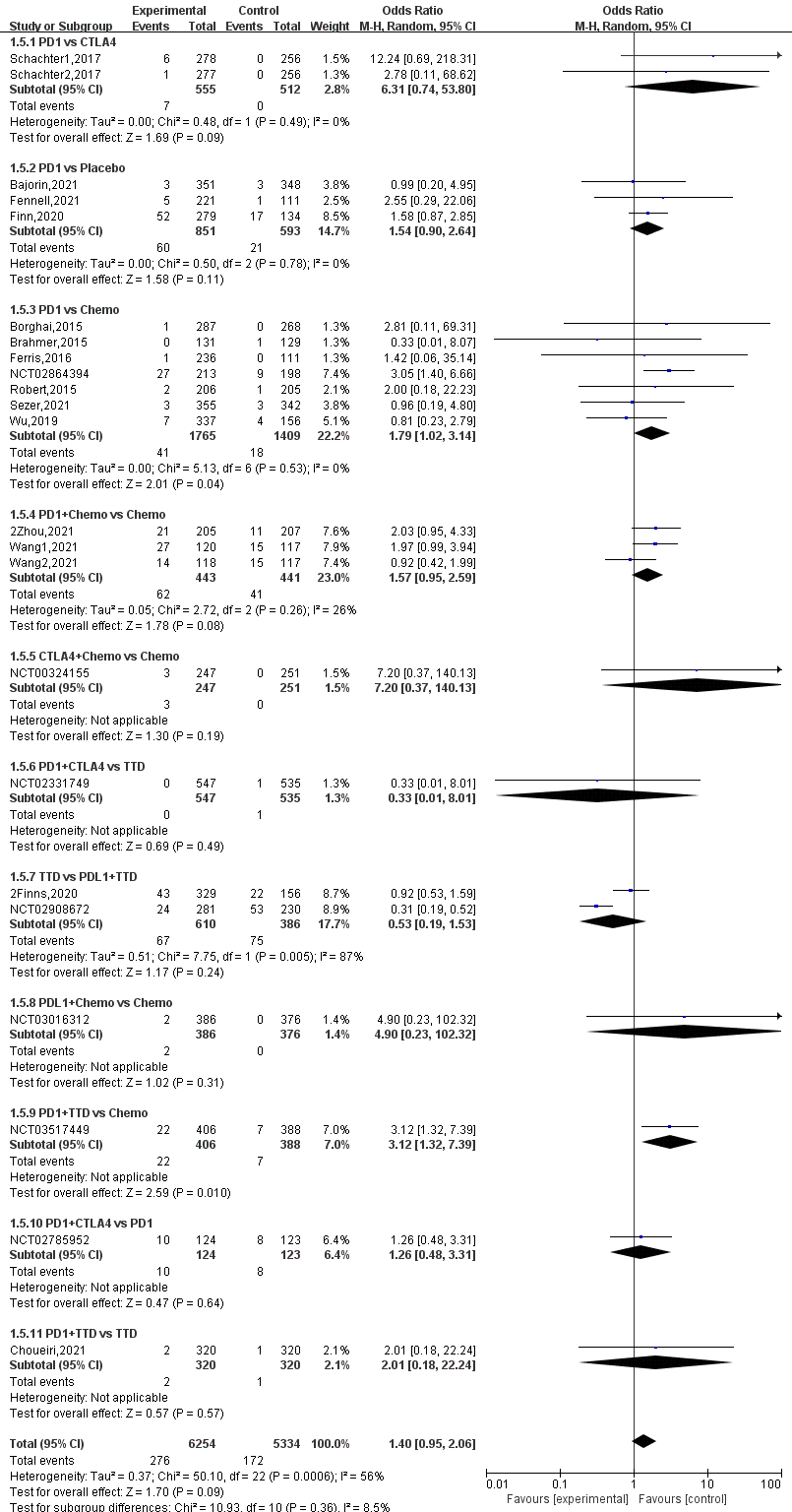
**

**J
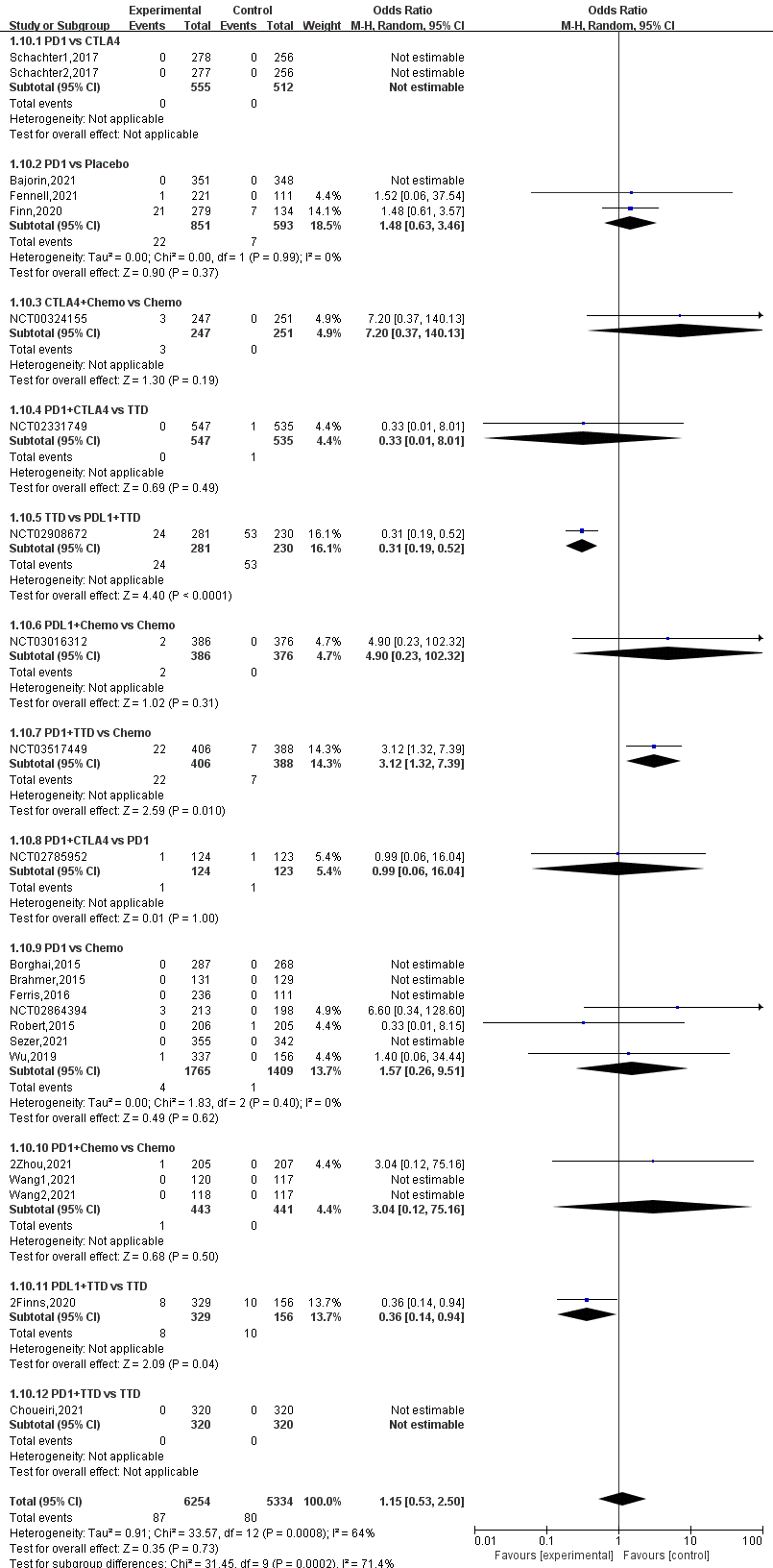
**

## **eFigure 4.2 Forest plot of the Immune-mediated hepatotoxicity by ICIs.**

（A：All-grade ALT；B：Grade 3-5 ALT；C：All-grade AST；D：Grade 3-5AST；E：All-grade ALP；F：Grade 3-5ALP；G：All-grade GGT；H：Grade 3-5GGT；I：All-grade Bilirubin；J：Grade 3-5 Bilirubin）

**A
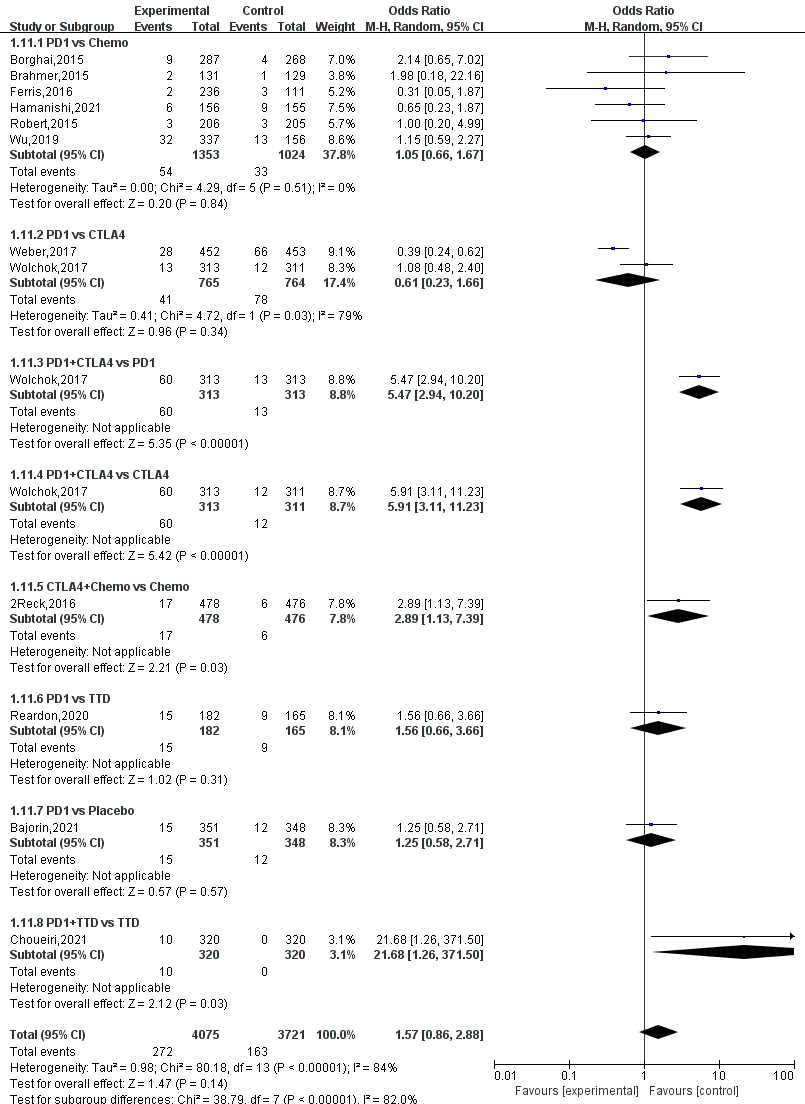
B
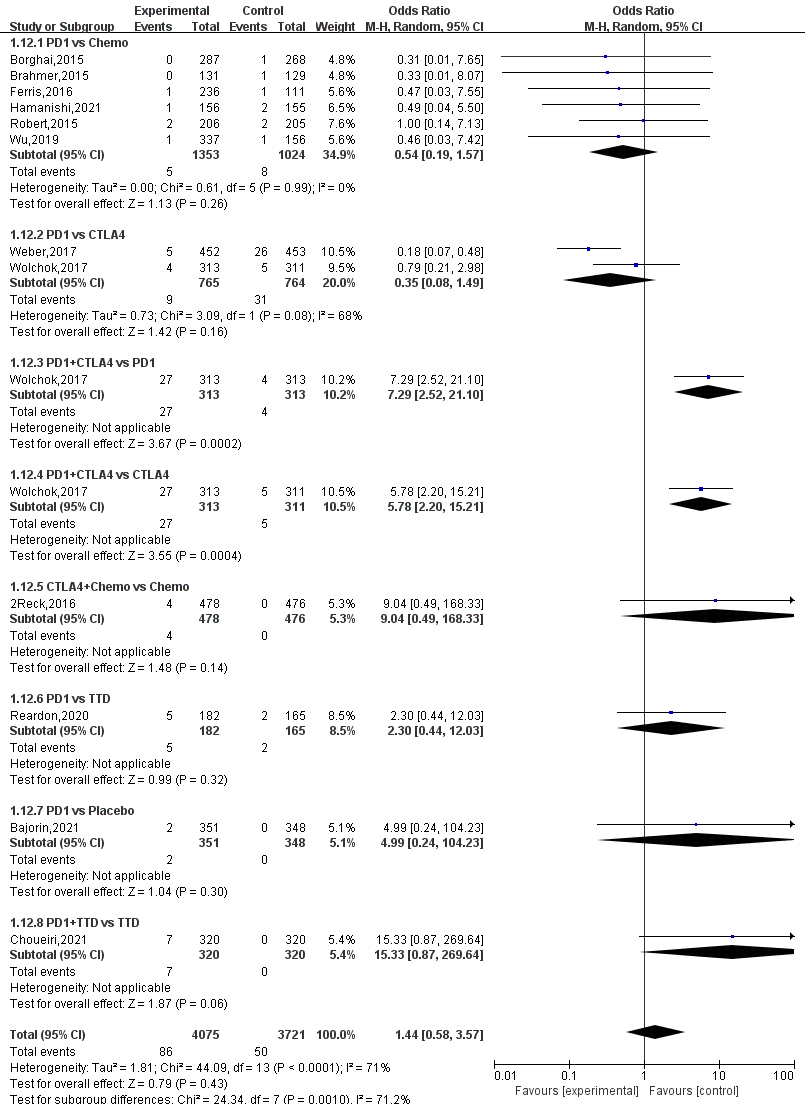
**

**C
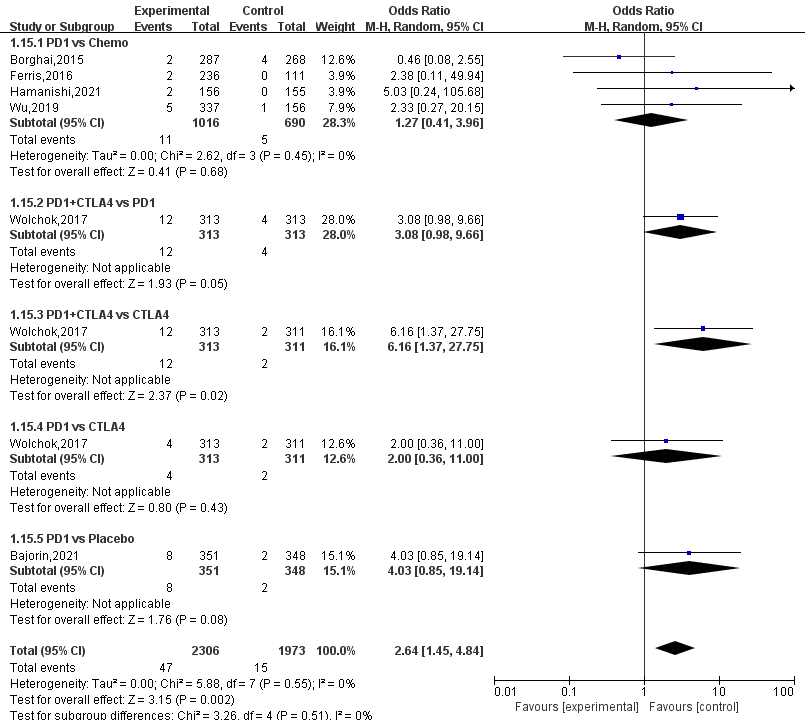
D
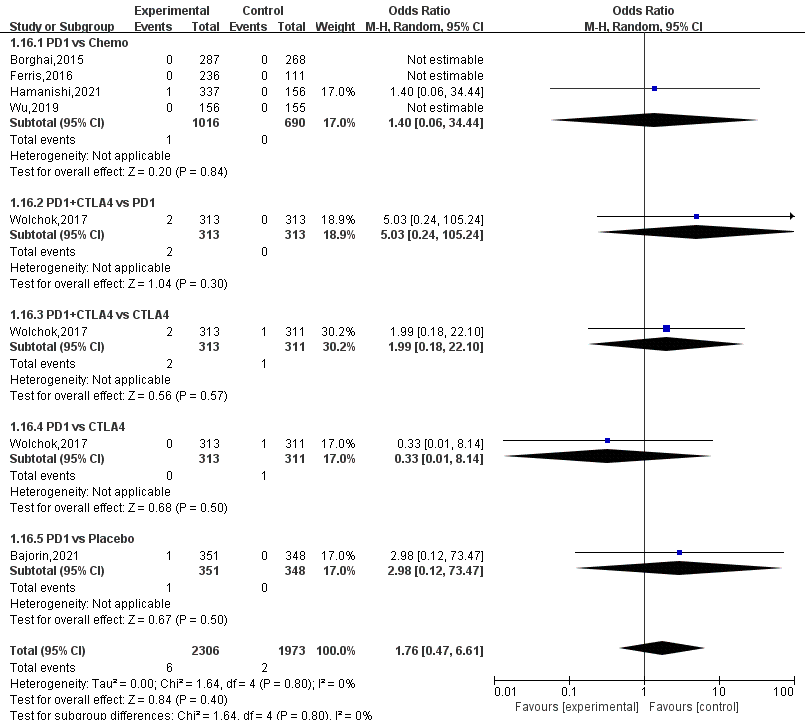
**

**E
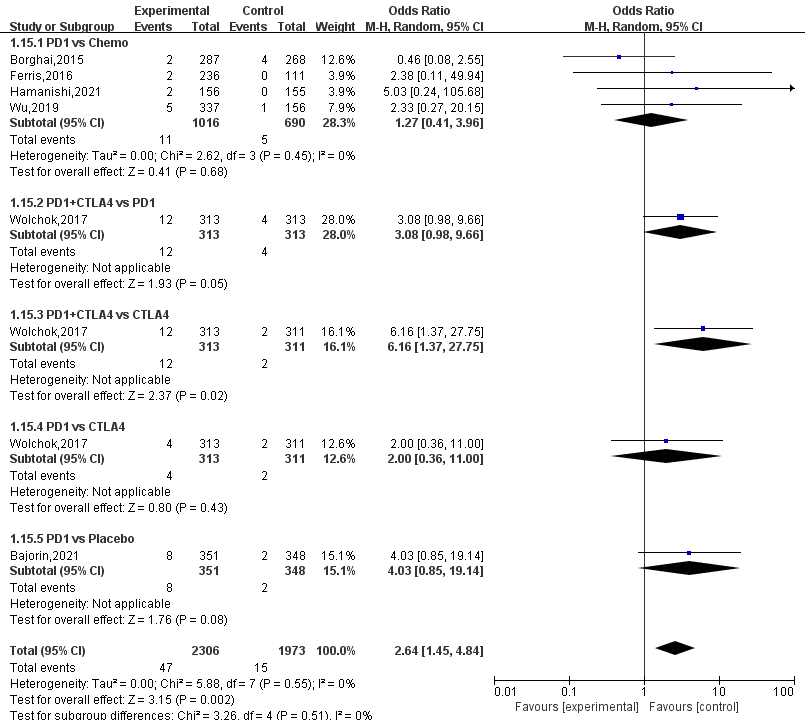
**

**F**
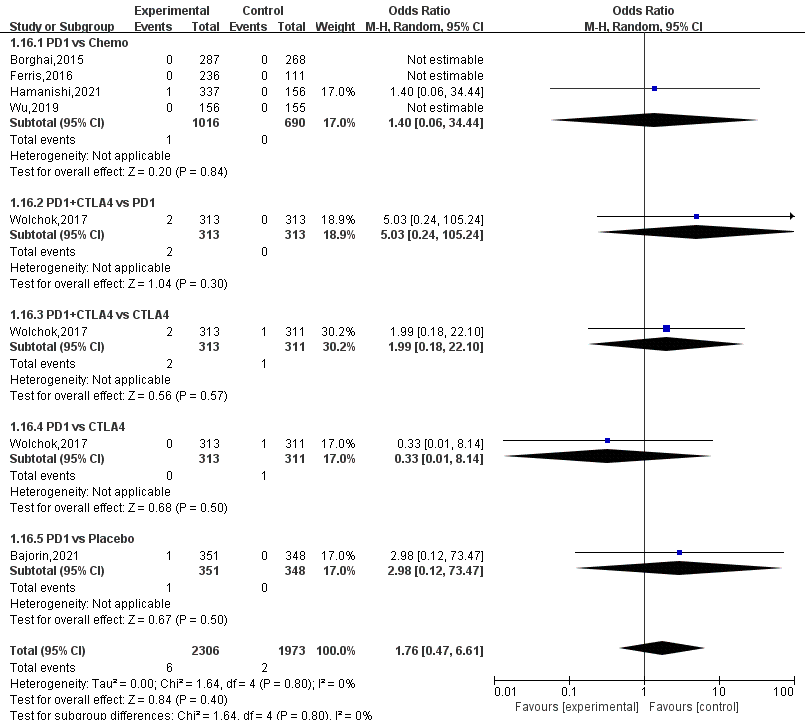


**G
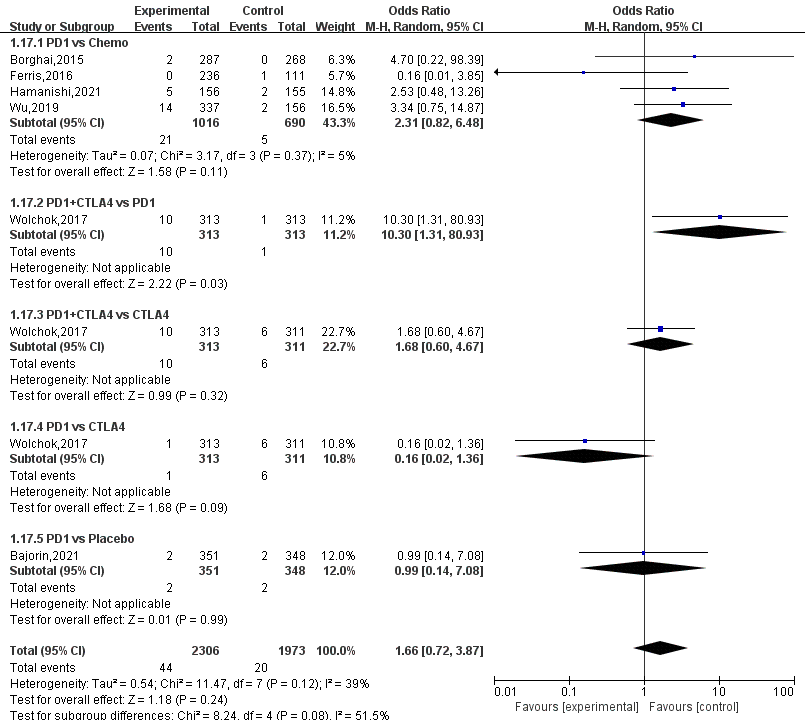
**

**H
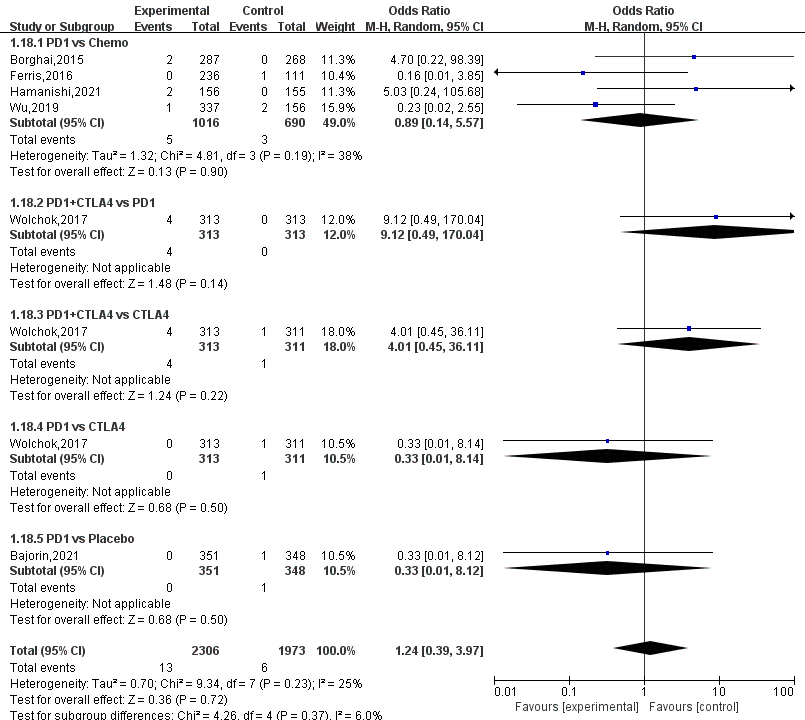
**

**I**
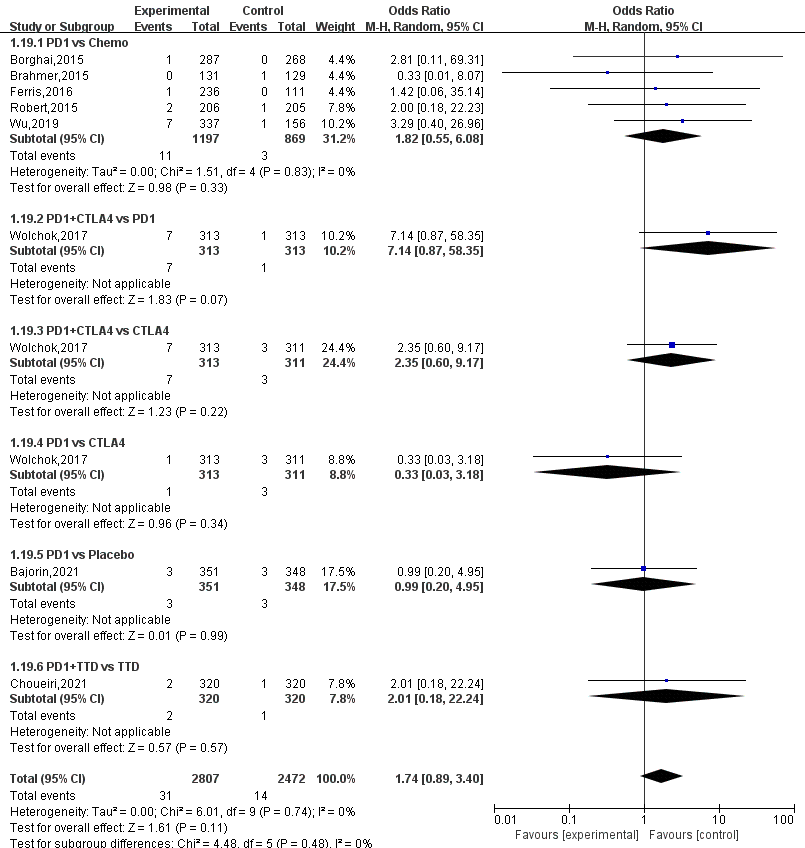


**J**
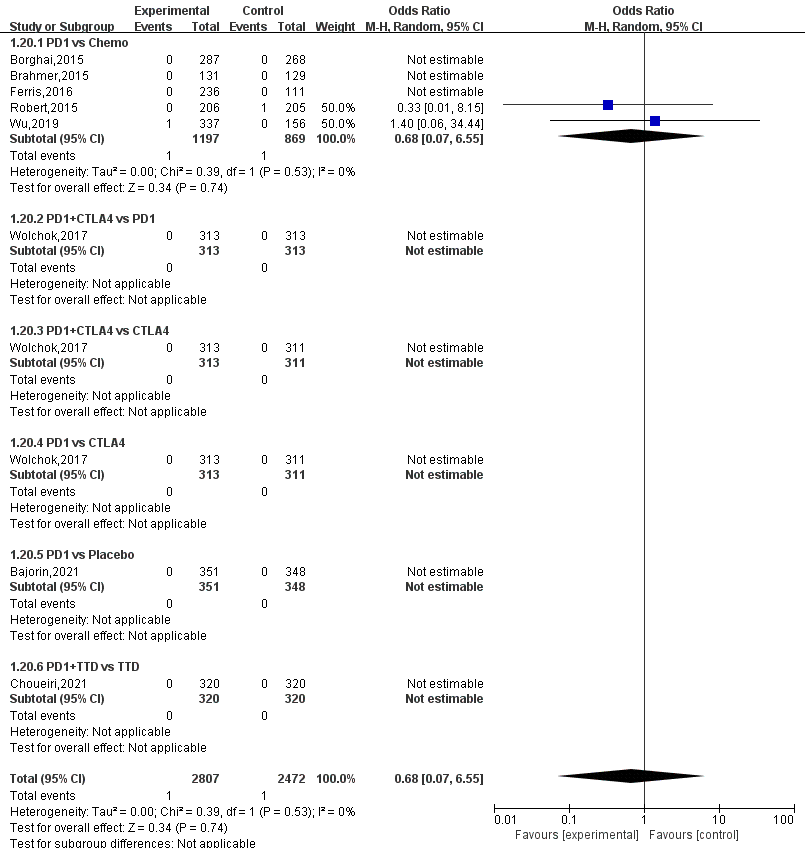


# 05 eFigure 5. Network plot

**(**A: Treatment-related ALT; B: Treatment-related AST; C：Treatment-related ALP; D：Treatment-related GGT; E：Treatment-related Bilirubin; F:Immune-mediated ALT G: Immune-mediated AST; H：Immune-mediated ALP; I：Immune-mediated; GGT ; J: Immune-mediated Bilirubin**)**


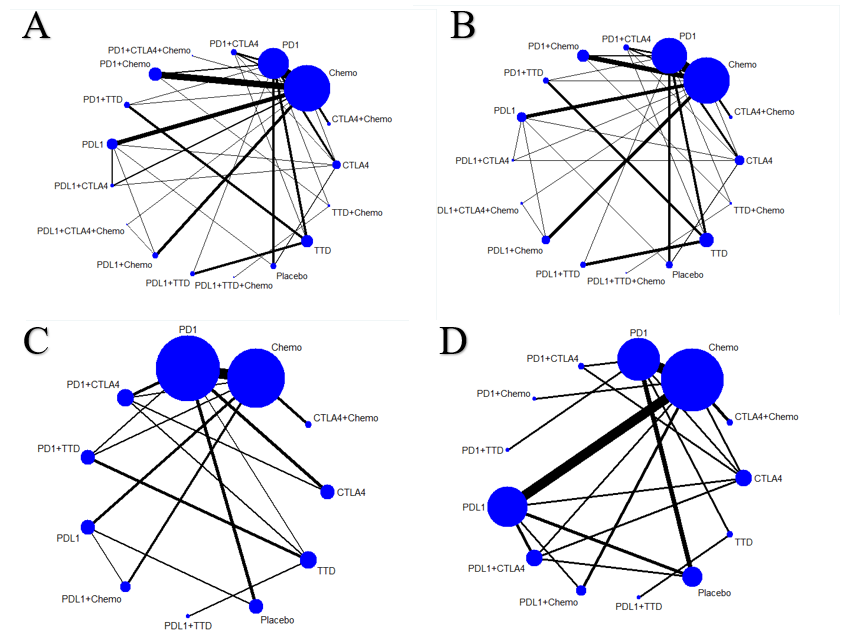


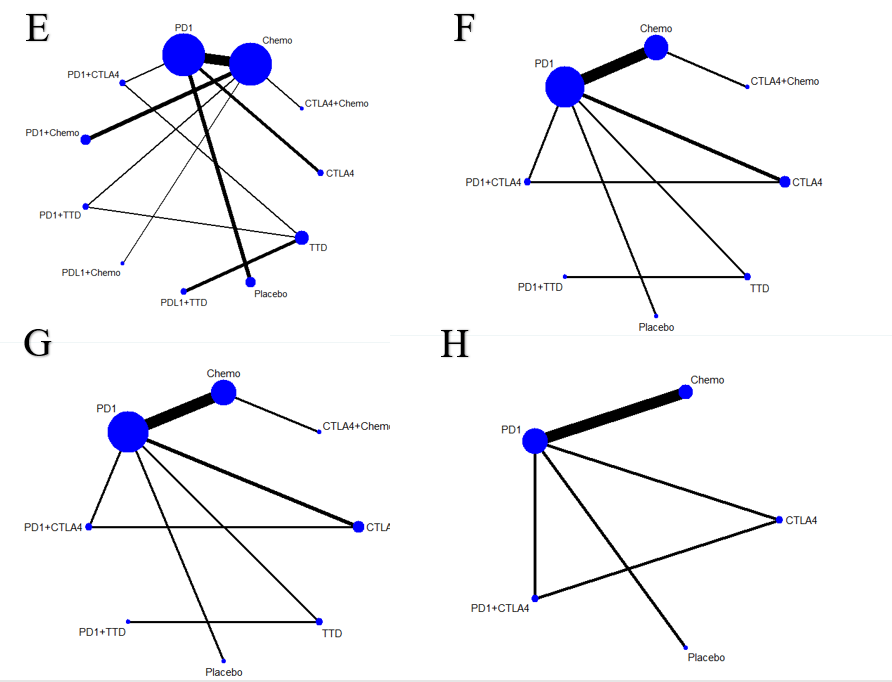


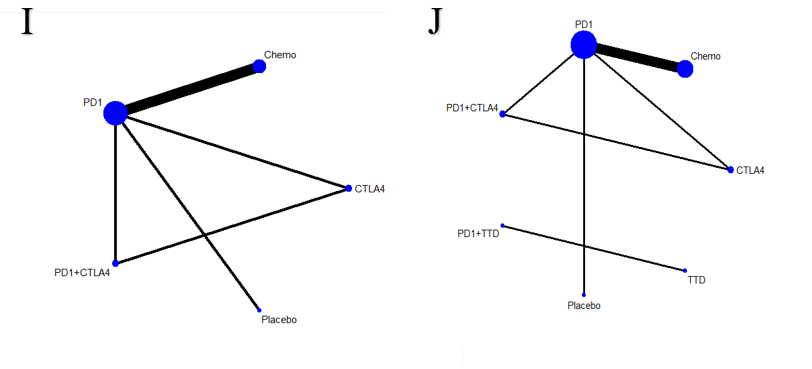


# 06 eFigure 6. Rankings of SUCRA for the risk of hepatotoxicity

eFigure 6.1 The distribution of Treatment-related Hepatotoxicity SUCRA values

(A：All-grade ALT/Grade 3-5 ALT；B: All-grade AST/Grade 3-5 AST; C: All-grade ALP/Grade 3-5 ALP; D: All-grade GGT/Grade 3-5 GGT; E: All-grade Bilirubin/ Grade 3-5 Bilirubin）


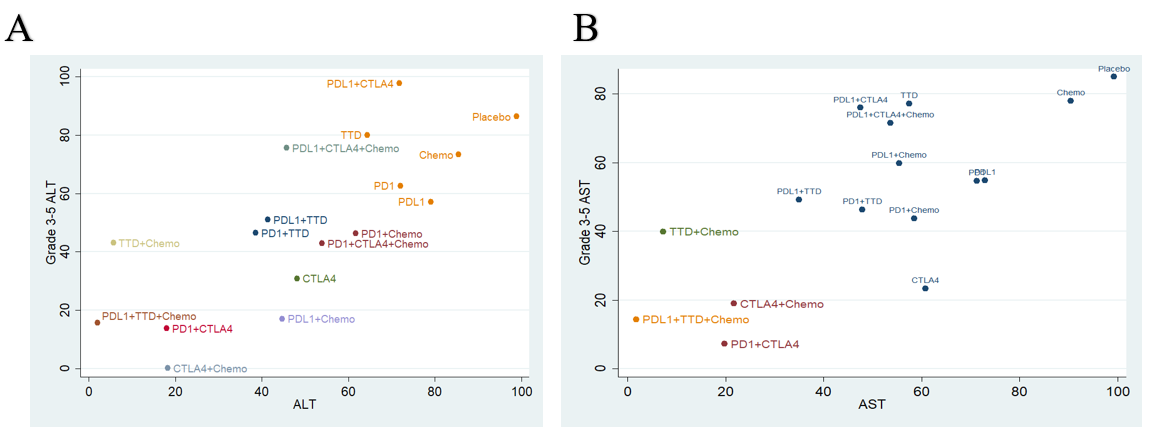


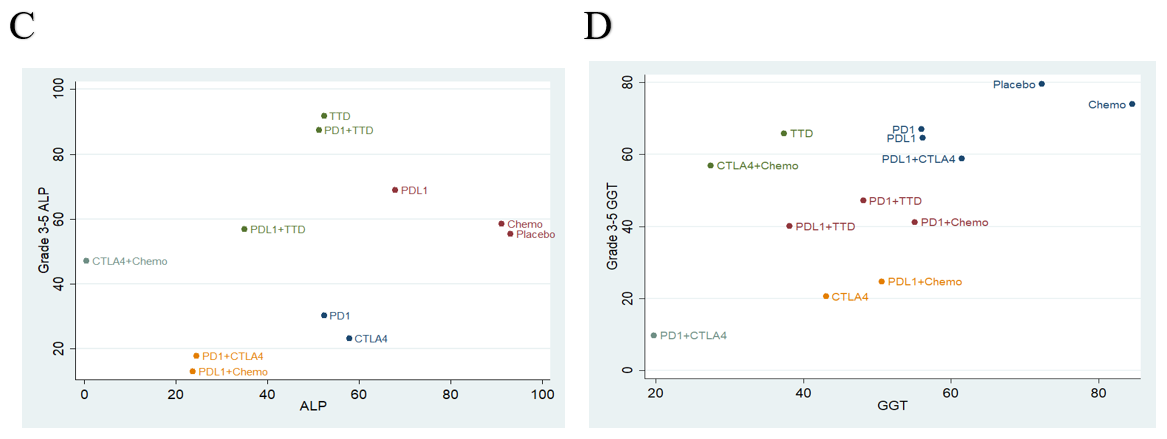


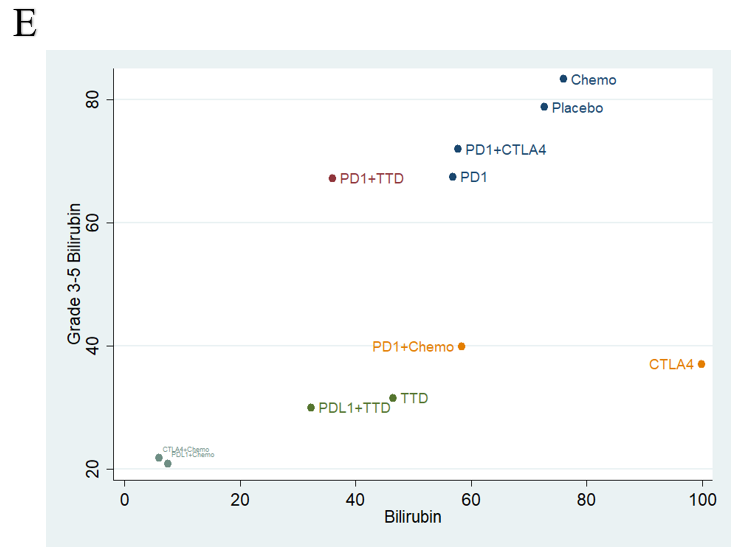


## **eFigure 6.2 The distribution of Immune-mediated Hepatotoxicity SUCRA values.**


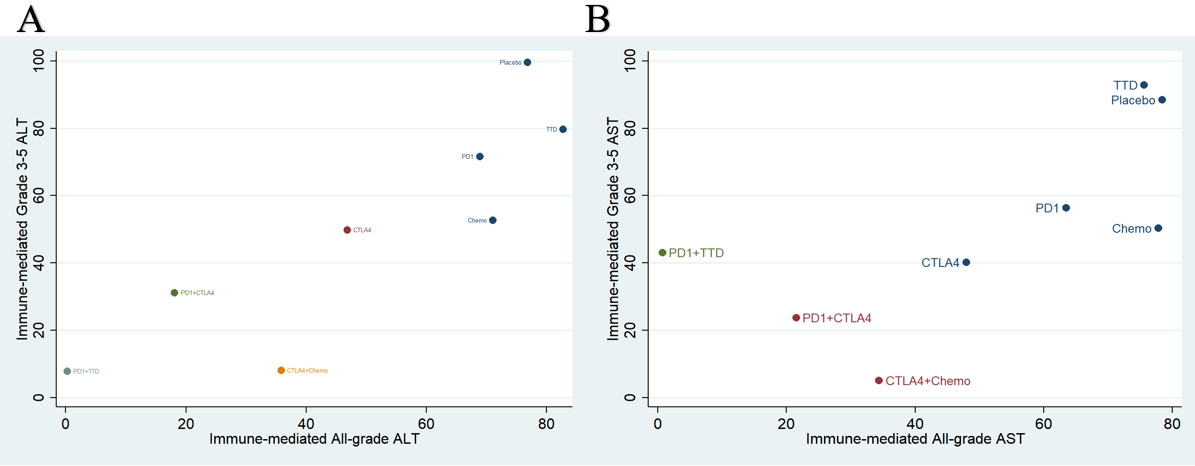


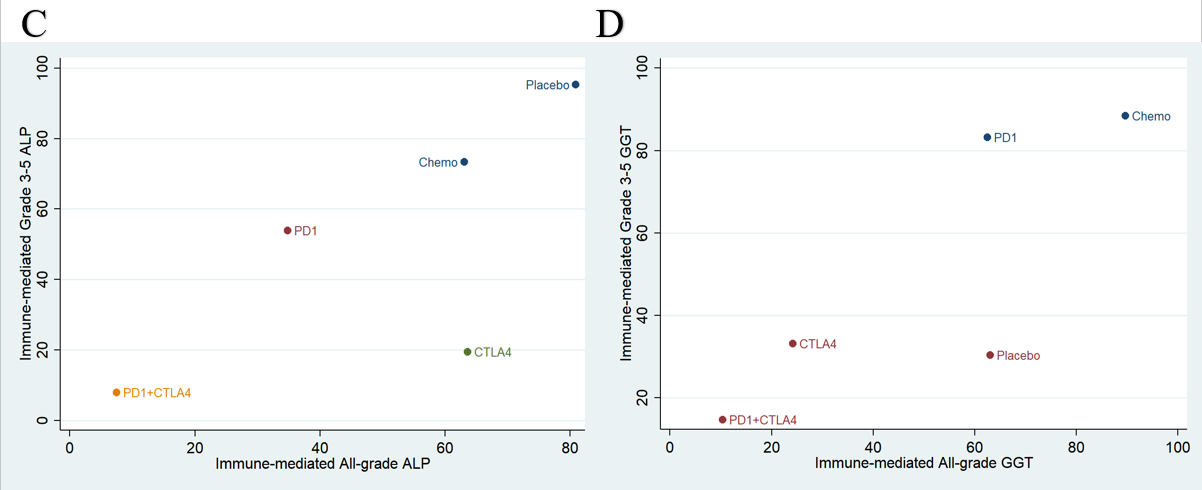


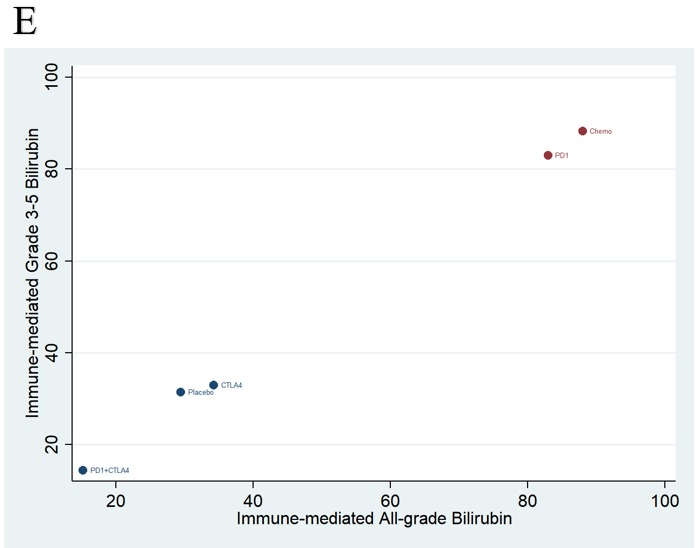


# 07 eFigure 7. Regimen ranking of fatal liver AEs

# 08 eFigure 8. Subgroup analysis by cancer type

## eFigure 8.1 Network plots of Treatment-related ALT by cancer type

(A: respiratory system cancer; B: urogenital system cancer; C: skin cancer; D: head and neck cancer; E: digest system cancer)


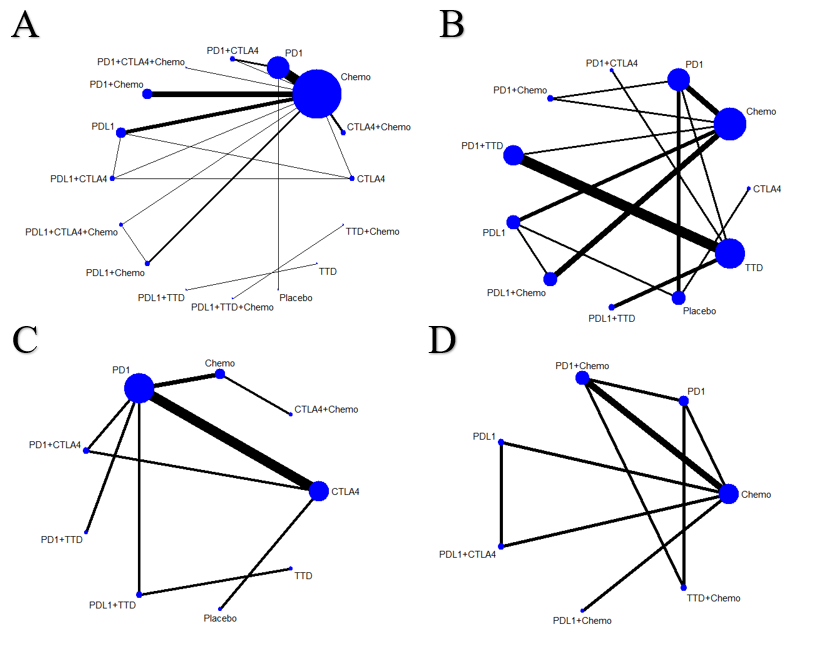


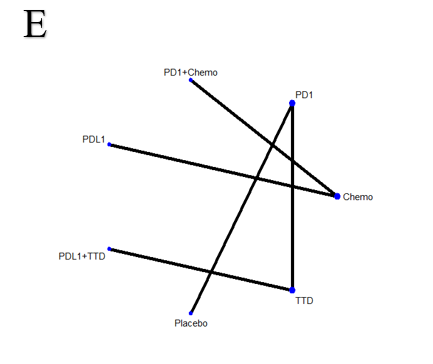


# 09 eFigure 9. Subgroup analysis of the risk of hepatotoxicity by dose

## eFigure 9.1 Subgroup analysis of the risk of hepatotoxicity by Nivolumab dose


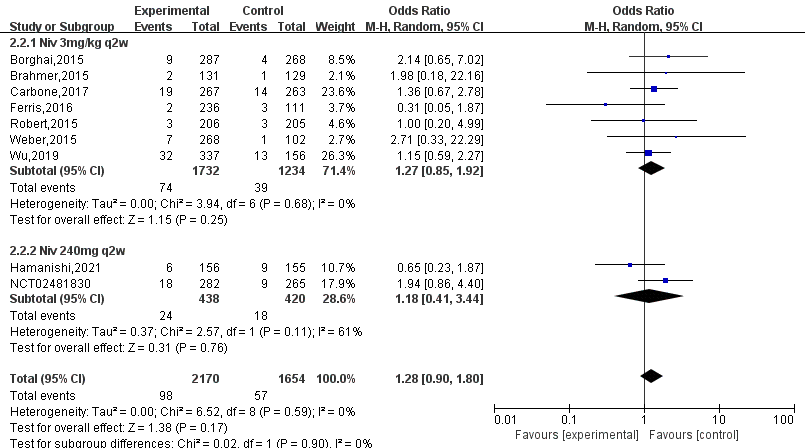


## eFigure 9.2 Subgroup analysis of the risk of hepatotoxicity by Atezolizumab dose


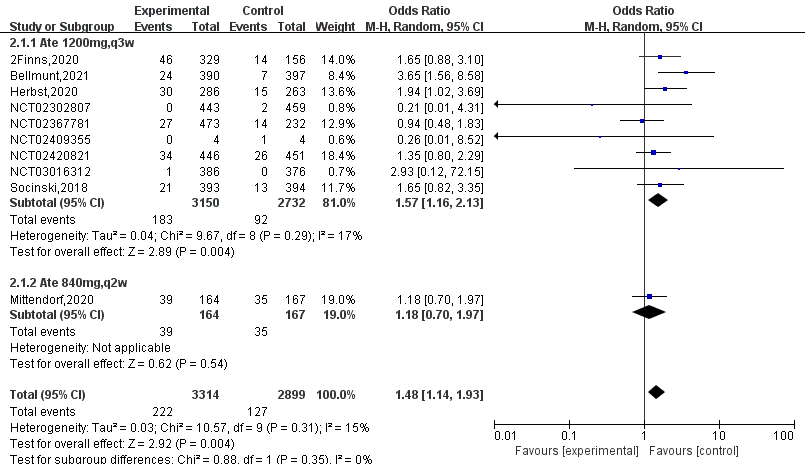


## eFigure 9.3 Subgroup analysis of the risk of hepatotoxicity by Atezolizumab dose

**
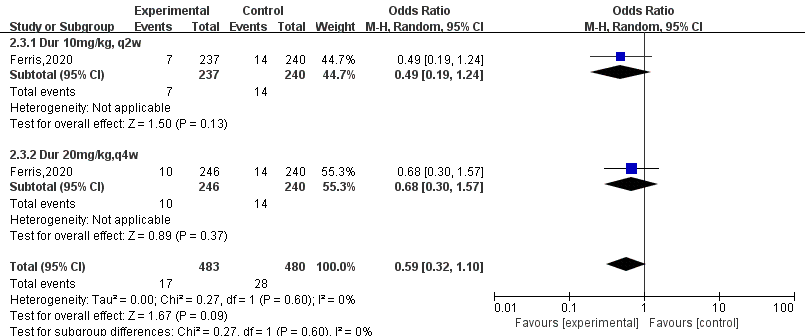
**

## eFigure 9.4 Subgroup analysis of the risk of hepatotoxicity by Ipilimumab dose

**
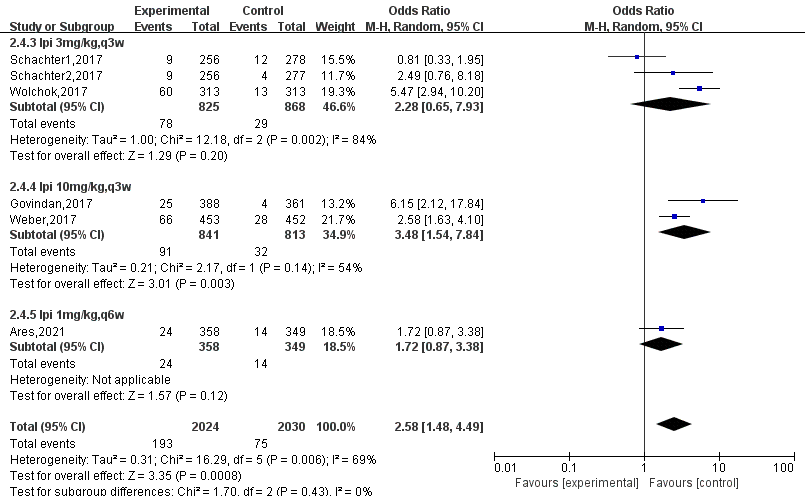
**

## eFigure 9.5 Subgroup analysis of the risk of hepatotoxicity by Pembrolizumab dose

**
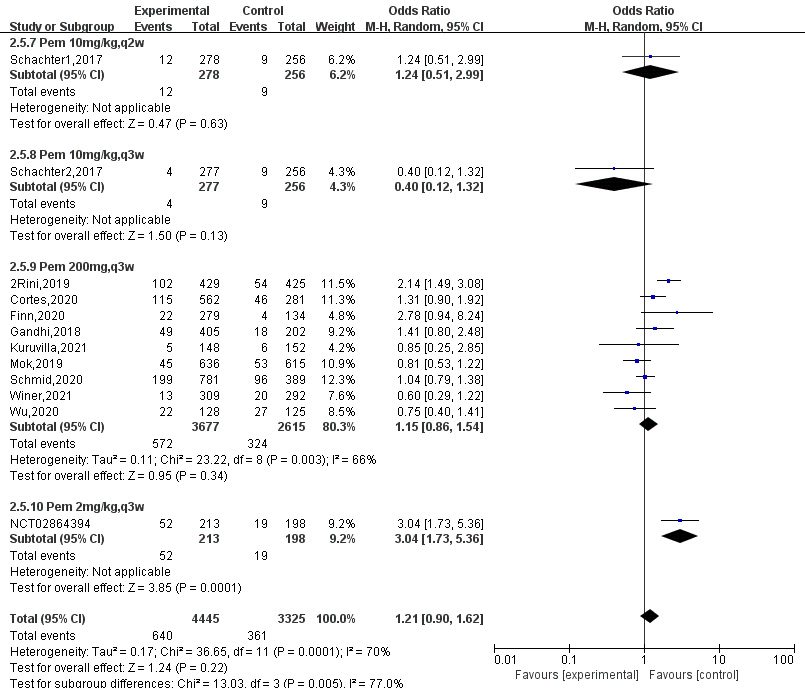
**

# 10 eTable 1. Literature search strategy

|  | Search strategy in PubMed |
| --- | --- |
| P | #1  “Neoplasms”[mh] OR Neoplasms[tiab] OR Neoplasm[tiab] OR Cancer[tiab] OR Cancers[tiab] OR Tumors [tiab] OR Tumor[tiab] OR  Malignancy[tiab] OR Malignancies[tiab] |
| I | #2  Programmed death ligand 1[tiab] OR PD-L1[tiab] OR Programmed death 1[tiab] OR PD-1[tiab] OR Cytotoxic T-lymphocyte antigen 4[tiab] OR CTLA-4[tiab] OR Immune Checkpoint Inhibitor[tiab] OR immune therapy[tiab] OR immunotherapy[tiab]  OR  Nivolumab[tiab] OR MDX-1106[tiab] OR ONO-4538[tiab] OR BMS-936558[tiab] OR Opdivo [tiab] OR  Pembrolizumab[tiab] OR lambrolizumab[tiab] OR Keytruda[tiab] OR MK-3475[tiab]  OR  Cemiplimab[tiab] OR Toripalimab[tiab] OR JS001[tiab]OR Sintilimab[tiab]  OR  Durvalumab [tiab]  OR  Atezolizumab[tiab] OR MPDL3280A[tiab] OR tecentriq[tiab] OR RG7446[tiab] OR RG-7446[tiab] OR  Avelumab[tiab] OR MSB0010718C[tiab]  OR  Ipilimumab[tiab] OR Anti CTLA 4 MAb Ipilimumab[tiab] OR Anti-CTLA-4 MAb Ipilimumab[tiab] OR Ipilimumab, Anti-CTLA-4 MAb[tiab] OR Yervoy[tiab] OR MDX010[tiab] OR MDX 010[tiab] OR MDX-010 M[tiab] OR MDX CTLA 4[tiab]  OR  Tremelimumab[tiab] OR ticilimumab[tiab] OR CP 675[Title/Abstract] OR CP675 cpd[tiab] OR CP-675[tiab] OR CP-675,206[tiab] OR CP-675206[tiab] OR CP675206[tiab] OR CP 675206[tiab]  OR  Camrelizumab[tiab]OR SHR-1210[tiab]OR SHR 1210[tiab] |
| S | #3  (randomized controlled trial[pt] OR controlled clinical trial[pt] OR randomized[tiab] OR placebo[tiab] OR clinical trials as topic[mesh:noexp] OR randomly[tiab] OR trial[ti]) NOT (animals[mh] NOT (humans[mh] AND animals[mh])) |
|  | #4  "2015/01/01"[Date - Publication] : "2021/11/30"[Date - Publication] |
|  | #5  #1AND#2AND#3 |
|  | Search strategy in Embase |
| P | #1  'neoplasms':ti,ab,kw OR 'neoplasm':ti,ab,kw OR 'cancer':ti,ab,kw OR 'cancers':ti,ab,kw OR 'tumors':ti,ab,kw OR 'tumor':ti,ab,kw OR 'malignancy':ti,ab,kw OR 'malignancies':ti,ab,kw OR 'neoplasms'/exp |
| I | #2  'programmed death ligand 1':ti,ab,kw OR 'pd-l1':ti,ab,kw OR 'programmed death 1':ti,ab,kw OR 'pd-1':ti,ab,kw OR 'cytotoxic t-lymphocyte antigen 4':ti,ab,kw OR 'ctla 4':ti,ab,kw OR 'immune checkpoint inhibitor':ti,ab,kw OR 'immune therapy':ti,ab,kw OR 'immunotherapy':ti,ab,kw OR 'nivolumab':ti,ab,kw OR 'mdx-1106':ti,ab,kw OR 'ono-4538':ti,ab,kw OR 'bms-936558':ti,ab,kw OR 'opdivo':ti,ab,kw OR 'pembrolizumab':ti,ab,kw OR 'lambrolizumab':ti,ab,kw OR 'keytruda':ti,ab,kw OR 'mk-3475':ti,ab,kw OR 'cemiplimab':ti,ab,kw OR 'toripalimab':ti,ab,kw OR 'JS001':ti,ab,kw OR 'sintilimab':ti,ab,kw OR 'durvalumab':ti,ab,kw OR 'atezolizumab':ti,ab,kw OR 'mpdl3280a':ti,ab,kw OR 'tecentriq':ti,ab,kw OR 'rg7446':ti,ab,kw OR 'rg-7446':ti,ab,kw OR 'avelumab':ti,ab,kw OR 'msb0010718c':ti,ab,kw OR 'ipilimumab':ti,ab,kw OR 'anti ctla 4 mab ipilimumab':ti,ab,kw OR 'anti-ctla-4 mab ipilimumab':ti,ab,kw OR 'ipilimumab, anti-ctla-4 mab':ti,ab,kw OR 'yervoy':ti,ab,kw OR 'mdx010':ti,ab,kw OR 'mdx 010':ti,ab,kw OR 'mdx-010 m':ti,ab,kw OR 'mdx ctla 4':ti,ab,kw OR 'tremelimumab':ti,ab,kw OR 'ticilimumab':ti,ab,kw OR 'cp 675':ti,ab,kw OR 'cp675 cpd':ti,ab,kw OR 'cp-675':ti,ab,kw OR 'cp-675,206':ti,ab,kw OR 'cp-675206':ti,ab,kw OR 'cp675206':ti,ab,kw OR 'cp 675206':ti,ab,kw OR 'Camrelizumab':ti,ab,kw OR 'SHR-1210':ti,ab,kw OR 'SHR 1210':ti,ab,kw |
| S | #3  'crossover procedure':de OR 'double-blind procedure':de OR 'randomized controlled trial':de OR 'single-blind procedure':de OR (random* OR factorial* OR crossover* OR cross NEXT/1 over* OR placebo* OR doubl* NEAR/1 blind* OR singl* NEAR/1 blind* OR assign* OR allocat* OR volunteer*):de,ab,ti |
|  | #4  #1 AND #2 AND #3 AND [2015-2021]/py |

|  | Search strategy in Web of science |
| --- | --- |
| P | #1  TS=((‘Neoplasms’ OR ‘Neoplasm’ OR ‘Cancer’ OR ‘Cancers’ OR ‘Tumors’ OR ‘Tumor’ OR ‘Malignancy’ OR ‘Malignancies’)) |
| I | #2  TS=('programmed death ligand 1' OR 'pd-l1' OR 'programmed death 1' OR 'pd-1' OR 'cytotoxic t-lymphocyte antigen 4' OR 'ctla 4' OR 'immune checkpoint inhibitor' OR 'immune therapy' OR 'immunotherapy' OR 'nivolumab' OR 'mdx-1106' OR 'ono-4538' OR 'bms-936558' OR 'optico' OR 'pembrolizumab' OR 'lambrolizumab' OR 'keytruda' OR 'mk-3475' OR 'cemiplimab' OR 'toripalimab' OR 'JS001' OR 'sintilimab' OR 'durvalumab' OR 'atezolizumab' OR 'mpdl3280a' OR 'tecentriq' OR 'rg7440' OR 'rg-7446' OR 'avelumab' OR 'msb0010718c' OR 'ipilimumab' OR 'anti ctla 4 mab ipilimumab' OR 'anti-ctla-4 mab ipilimumab' OR 'ipilimumab, anti-ctla-4 mab' OR 'yerkoy' OR 'mdx210' OR 'mdx 010' OR 'mdx-010 m' OR 'mdx ctla 4' OR 'tremelimumab' OR 'tocilizumab' OR 'cp 675' OR 'cp655 cpd' OR 'cp-675' OR 'cp-675,206' OR 'cp-672206' OR 'cp65207' OR 'cp 672206' OR 'Camrelizumab' OR 'SHR-1210' OR 'SHR 1210' ) |
| S | #3  TS=("randomized controlled trial" OR "controlled clinical trial" OR "clinical trial" OR "random*" OR "rct*" OR  "crossover" OR "masked” OR “blind*" OR "placebo*") |
|  | #4  DOP=(2015-01-01/2021-11-30) |
|  | #5  #1AND#2AND#3AND#4 |

|  | Search strategy in Cochrane Library |
| --- | --- |
| P | #1  MeSH descriptor: [Neoplasms] explode all trees  #2  (Neoplasms OR Neoplasm OR Cancer OR Cancers OR Tumors OR Tumor OR Malignancy OR Malignancies):ti,ab,kw  #3  #1OR#2 |
| I | #4  (“Programmed death ligand 1” OR PD-L1 OR “Programmed death 1” OR PD-1 OR “Cytotoxic T-lymphocyte antigen 4” OR CTLA-4 OR “Immune Checkpoint Inhibitor” OR “immune therapy” OR immunotherapy OR Nivolumab OR MDX-1106 OR ONO-4538 OR BMS-936558 OR Opdivo OR Pembrolizumab OR lambrolizumab OR Keytruda OR MK-3475 OR Cemiplimab OR Toripalimab OR JS001 OR Sintilimab OR Durvalumab OR Atezolizumab OR MPDL3280A OR tecentriq OR RG7446 OR RG-7446 OR Avelumab OR MSB0010718C OR Ipilimumab OR “Anti CTLA 4 MAb Ipilimumab” OR “Anti-CTLA-4 MAb Ipilimumab” OR “Ipilimumab, Anti-CTLA-4 MAb” OR Yervoy OR MDX010 OR “MDX 010” OR “MDX-010 M” OR “MDX CTLA 4” OR Tremelimumab OR ticilimumab OR “CP 675” OR “CP675 cpd” OR CP-675 OR “CP-675,206” OR CP-675206 OR CP675206 OR “CP 675206” OR Camrelizumab OR SHR-1210 OR “SHR 1210”):ti,ab,kw |
|  | #5  #3 #3 AND #4 with Publication Year from 2015 to 2021, in Trials |

| Search strategy in Scopus |
| --- |
| ( TITLE-ABS KEY ( neoplasms  OR  neoplasm  OR  cancer  OR  cancers  OR  tumors  OR  tumor  OR  malignancy  OR  malignancies )  AND  TITLE-ABS-KEY ( "Programmed death ligand 1"  OR  pd-l1  OR  "Programmed death 1"  OR  pd-1  OR  "Cytotoxic T-lymphocyte antigen 4"  OR  ctla-4  OR  "Immune Checkpoint Inhibitor"  OR  "immune therapy"  OR  immunotherapy  OR  nivolumab  OR  mdx-1106  OR  ono-4538  OR  bms-936558  OR  opdivo  OR  pembrolizumab  OR  lambrolizumab  OR  keytruda  OR  mk-3475  OR  cemiplimab  OR  toripalimab  OR JS001 OR  sintilimab  OR  durvalumab  OR  atezolizumab  OR  mpdl3280a  OR  tecentriq  OR  rg7446  OR  rg-7446  OR  avelumab  OR  msb0010718c  OR  ipilimumab  OR  "Anti CTLA 4 MAb Ipilimumab"  OR  "Anti-CTLA-4 MAb Ipilimumab"  OR  "Ipilimumab, Anti-CTLA-4 MAb"  OR  yervoy  OR  mdx010  OR  "MDX 010"  OR  "MDX-010 M"  OR  "MDX CTLA 4"  OR  tremelimumab  OR  ticilimumab  OR  "CP 675"  OR  "CP675 cpd"  OR  cp-675  OR  "CP-675,206"  OR  cp-675206  OR  cp675206  OR  "CP 675206" OR Camrelizumab OR SHR-1210 OR "SHR 1210" )  AND  INDEXTERMS ( "clinical trials"  OR  "clinical trials as a topic"  OR  "randomized controlled trial"  OR  "Randomized Controlled Trials as Topic"  OR  "controlled clinical trial"  OR  "Controlled Clinical Trials"  OR  "random allocation"  OR  "Double-Blind Method"  OR  "Single-Blind Method"  OR  "Cross-Over Studies"  OR  "Placebos"  OR  "multicenter study"  OR  "double blind procedure"  OR  "single blind procedure"  OR  "crossover procedure"  OR  "clinical trial" ) )  AND  PUBYEAR  >  2014  AND  PUBYEAR  <  2022 |

| Search strategy in CINAHL | |
| --- | --- |
| p | S1  (MH "Neoplasms")  S2  TX Neoplasms OR Neoplasm OR Cancer OR Cancers OR Tumors OR Tumor OR Malignancy OR Malignancies  S3  S1 OR S2 |
| I | S4 TX “Programmed death ligand 1” OR PD-L1 OR “Programmed death 1” OR PD-1 OR “Cytotoxic T-lymphocyte antigen 4” OR CTLA-4 OR “Immune Checkpoint Inhibitor” OR “immune therapy” OR immunotherapy OR Nivolumab OR MDX-1106 OR ONO-4538 OR BMS-936558 OR Opdivo OR Pembrolizumab OR lambrolizumab OR Keytruda OR MK-3475 OR Cemiplimab OR Toripalimab OR JS001 OR Sintilimab OR Durvalumab OR Atezolizumab OR MPDL3280A OR tecentriq OR RG7446 OR RG-7446 OR Avelumab OR MSB0010718C OR Ipilimumab OR “Anti CTLA 4 MAb Ipilimumab” OR “Anti-CTLA-4 MAb Ipilimumab” OR “Ipilimumab, Anti-CTLA-4 MAb” OR Yervoy OR MDX010 OR “MDX 010” OR MDX-010 M OR “MDX CTLA 4” OR Tremelimumab OR ticilimumab OR “CP 675” OR “CP675 cpd” OR CP-675 OR “CP-675,206” OR CP-675206 OR CP675206 OR “CP 675206” OR Camrelizumab OR SHR-1210 OR "SHR 1210" |
| S | S5  TX allocat* random* OR (MH "Quantitative Studies") OR (MH "Placebos") OR TX placebo* OR TX random* allocat* OR (MH "Random Assignment") OR TX randomi* control* trial* OR TX ((singl* n1 blind*) OR (singl* n1 mask*)) OR TX ((doubl* n1 blind*) OR (doubl* n1 mask*)) OR TX ( (tripl* n1 blind*) OR (tripl* n1 mask*)) OR TX ((trebl* n1 blind*) OR (trebl* n1 mask*) ) OR TX clinic* n1 trial* OR PT Clinical trial OR (MH "Clinical Trials+") |
|  | S3 AND S4 AND S5  Limiters - Linked Full Text; Abstract Available; Published Date: 20150101-20211231 |

| Search strategy in PsycINFO | |
| --- | --- |
| p | 1   (Neoplasms or Neoplasm or Cancer or Cancers or Tumors or Tumor or Malignancy or Malignancies).mp. [mp=title, abstract, heading word, table of contents, key concepts, original title, tests & measures, mesh word] |
| I | 2  (Programmed death ligand 1 or PD-L1 or Programmed death 1 or PD-1 or Cytotoxic T-lymphocyte antigen 4 or CTLA-4 or Immune Checkpoint Inhibitor or immune therapy or immunotherapy or Nivolumab or MDX-1106 or ONO-4538 or BMS-936558 or Opdivo or Pembrolizumab or lambrolizumab or Keytruda or MK-3475 or Cemiplimab or Toripalimab or JS001 or Sintilimab or Durvalumab or Atezolizumab or MPDL3280A or tecentriq or RG7446 or RG-7446 or Avelumab or MSB0010718C or Ipilimumab or Anti CTLA 4 MAb Ipilimumab or Anti-CTLA-4 MAb Ipilimumab or Ipilimumab, Anti-CTLA-4 MAb or Yervoy or MDX010 or "MDX 010" or MDX-010 M or MDX CTLA 4 or Tremelimumab or ticilimumab or CP 675 or CP675 cpd or CP-675 or CP-675,206 or CP-675206 or CP675206 or CP 675206 or Camrelizumab or SHR-1210 or SHR 1210).mp. [mp=title, abstract, heading word, table of contents, key concepts, original title, tests & measures, mesh word] |
| S |  |
|  | 3  1 AND 2  limit 3 to yr="2015 - 2021" |

# 11 eTable 2. Main characteristics of the trials included in this meta-analysis

| **Study** | **Trial name** | **NCT number** | **Patients** | **Cancer Type** | **Country** | **line of  treatment** | **Disease Stage** | **Treatment** | **Liver metastatic** | **SampleSize** | **Age(range)** | **male(n,%)** | **Median Follow-up (months)** |
| --- | --- | --- | --- | --- | --- | --- | --- | --- | --- | --- | --- | --- | --- |
| **Ares,2018** | KEYNOTE-407 | NCT2775435 | Squamous NSCLC | respiratory | 17 countries | 1 | stage IV | Pem+Chemo | NA | 278 | 65 (29–87) | 220 (79.1) | 7.8 |
|  |  |  |  |  |  |  |  | Chemo | NA | 281 | 65 (36–88) | 235 (83.6) | 7.8 |
| **Bellmunt,2017** | KEYNOTE-045 | NCT02256436 | Urothelial Carcinoma | urogenital | 29 countries | 2 | Advanced | Pem | 91(33.7) | 270 | 67(29-88) | 200 (74.1) | 14.1 |
|  |  |  |  |  |  |  |  | Chemo | 95(35.1) | 272 | 65(26-84) | 202 (74.3) | 14.1 |
| **Borghaei,2015** | CheckMate 057 | NCT1673867 | Nonsquamous NSCLC | respiratory | unknown | ≥2 | stage IIIB or IV | Niv | NA | 292 | 61(37-84) | 151(52) | 13.2 |
|  |  |  |  |  |  |  |  | Chemo | NA | 290 | 64(21-85) | 168(58) | 13.2 |
| **Brahmer,2015** | CheckMate 017 | NCT1642004 | Squamous-Cell NSCLC | respiratory | global | 2 | IIIB or IV | Niv | NA | 135 | 62(39-85) | 111(82) | minimum11 |
|  |  |  |  |  |  |  |  | Chemo | NA | 137 | 64(42-84) | 97(71) | minimum11 |
| **Carbone,2017** | CheckMate 026 | NCT2041533 | NSCLC | respiratory | unknown | 1 | Stage IV or Recurrent | Niv | 54(20) | 271 | 63(32-89) | 184(68) | 13.5 |
|  |  |  |  |  |  |  |  | Chemo | 36(13) | 270 | 65(29-87) | 148(55) | 13.5 |
| **Ferris,2016** | CheckMate 141 | NCT02105636 | Squamous-Cell Carcinoma of the Head and Neck | Head and Neck | global | ≥2 | Recurrent | Niv | NA | 240 | 59(29-83) | 197(82.1) | 5.1 |
|  |  |  |  |  |  |  |  | Chemo | NA | 121 | 61(28-78) | 103(85.1) | 5.1 |
| **Robert,2015** | CheckMate 066 | NCT01721772 | Previously Untreated Melanoma without BRAF Mutation | skin | Canada; Australia or South America | 1 | stage III or IV | Niv | NA | 210 | 64(18-86) | 121(57.6) | 8.9 |
|  |  |  |  |  |  |  |  | Chemo(dacarbazine) | NA | 208 | 66(26-87) | 125(60.1) | 6.8 |
| **Schachter,2017** | KEYNOTE-006 | NCT01866319 | advanced melanoma | skin | 16 countries | all | stage III or IV | Pem every 2 | NA | 279 | 61(18-89) | 161(58) | 22.9 |
|  |  |  |  |  |  |  |  | Pem every 3 | NA | 277 | 63(22-89) | 174(63) | 22.9 |
|  |  |  |  |  |  |  |  | Ipi | NA | 278 | 62(18-88) | 162(58) | 22.9 |
| **Weber,2015** | CheckMate 037 | NCT01721746 | advanced  melanoma | skin | unknown | 2 | stage IIIC or IV | Niv | NA | 272 | 59(23-88) | 176(65) | 8.4 |
|  |  |  |  |  |  |  |  | Chemo | NA | 133 | 62(29-85) | 85(64) | 8.4 |
| **Govindan,2017** | Study-104 | NCT 01285609 | Squamous NSCLC | respiratory | America | 1 | stage IV or  recurrent Chemotherapy-naive | Ipi+Chemo | NA | 388 | 64 (28-84) | 326(84) | 12.5 |
|  |  |  |  |  |  |  |  | Chemo | NA | 361 | 64 (28-85) | 309(52) | 11.8 |
| **Gandhi,2018** | KEYNOTE-189 | NCT02578680 | nonsquamous NSCLC | respiratory | global | 1 | Metastatic | Pem+Chemo | NA | 410 | 65(34.0–84.0) | 254 (62.0) | 10.5 |
|  |  |  |  |  |  |  |  | Chemo | NA | 206 | 63.5 (34.0–84.0) | 109 (52.9) | 10.5 |
| **Mok,2019** | KEYNOTE-042 | NCT2220894 | NSCLC | respiratory | 213 sites | 1 | Advanced | Pem | NA | 637 | 63·0 (57·0–69·0) | 450 (71%) | 12.8 |
|  |  |  |  |  |  |  |  | Chemo | NA | 637 | 63·0 (57·0–69·0) | 452 (71%) | 12.8 |
| **Rittmeyer,2017** | OAK | NCT02008227 | NSCLC | respiratory | 31 countries | ≥2 | stage IIIB or IV | Ate | NA | 425 | 63(33-82) | 261(61) | 21 |
|  |  |  |  |  |  |  |  | Dox | NA | 425 | 64(34-85) | 259(61) | 21 |
| **Eggermont,2015** | EORTC 18071 | NCT00636168 | melanoma | skin | 19 countries | ≥2 | stage III | Ipi | NA | 475 | 51（20-84） | 296(62) | 63.6 |
|  |  |  |  |  |  |  |  | placebo | NA | 476 | 52（18-78） | 293(62) | 63.6 |
| **Eng1,2019** | IMblaze370 | NCT02788279 | colorectal  cancer | digestive | 11 countries | ≥2 | advanced or  metastatic | Ate+Regorafenib | 121 (66%) | 183 | 58 (51–67) | 107(58) | 7.3 |
|  |  |  |  |  |  |  |  | Ate | 57 (63%) | 90 | 56 (51–64) | 59(66) | 7.3 |
|  |  |  |  |  |  |  |  | Regorafenib | 59 (66%) | 90 | 59 (52–66) | 51 (57) | 7.3 |
| **Shitara,2018** | KEYNOTE-061 | NCT02370498 | gastric or gastro-oesophageal junction cancer | digestive | 30 countries | ≥2 | advanced | Pem | NA | 296 | 62.5 (54–70) | 202 (68) | 7.9 |
|  |  |  |  |  |  |  |  | paclitaxel | NA | 296 | 60.0 (53–68) | 208 (70) | 7.9 |
| **Kang,2017** | ONO-4538-12, ATTRACTION-2 | NCT02267343 | advanced gastric or  gastro-oesophageal junction cancer | digestive | Japan,  South Korea, and Taiwan | ≥2 | unresectable advanced or recurrent | Niv | 78 (24%) | 330 | 62 (54–69) | 229 (69) | 8.87 |
|  |  |  |  |  |  |  |  | Placebo | 28(17%) | 163 | 62 (54–69) | 119 (73) | 8.59 |
| **Finn2020** | KEYNOTE-240 | NCT02702401 | Advanced Hepatocellular Carcinoma | digestive | 27 countries | 2 | stage C disease or stage B disease | Pem | NA | 278 | 67(18-91) | 226(81.3) | 13.8 |
|  |  |  |  |  |  |  |  | Placebo | NA | 135 | 65(23-89) | 112(83) | 10.6 |
| **Cohen,2019** | KEYNOTE-040 | NCT02252042 | head-and-neck  squamous cell carcinoma | Head and Neck | 20 countries | ≥2 | recurrent or  metastatic | Pem | NA | 247 | 60·0 (55–66) | 207 (84%) | 7.5 |
|  |  |  |  |  |  |  |  | Placebo | NA | 248 | 60·0 (54–66) | 205 (83%) | 7.1 |
| **Ferris,2020** | EAGLE | NCT02369874 | metastatic head and neck squamous cell carcinoma | Head and Neck | unknown | ≥2 | progression or recurrence | Dur | NA | 240 | 59.0 (24 -84) | 202 (84.2) | 7.6 |
|  |  |  |  |  |  |  |  | Dur+TRE | NA | 247 | 61.0 (23 -81) | 209 (84.6) | 6.3 |
|  |  |  |  |  |  |  |  | Chemo | NA | 249 | 61.0 (22 -82) | 207(83.1) | 7.8 |
| **Rizvi,2020** | MYSTIC | NCT02453282 | NSCLC | respiratory | 17 countries | 1 | stage IV | Dur | NA | 163 | 64.0 (32-84) | 113 (69.3) | 30.2 |
|  |  |  |  |  |  |  |  | Dur+Treme | NA | 163 | 65.0 (34-87) | 118 (72.4) | 30.2 |
|  |  |  |  |  |  |  |  | Chemo | NA | 163 | 64.5 (35-85) | 106 (65.4) | 30.2 |
| **Weber,2017** | CheckMate 238 | NCT02388906 | Melanoma | skin | unknown | unknown | IIIB, IIIC, or IV | Niv | NA | 453 | 56 (19–83) | 258 (57.0) | 19.5 |
|  |  |  |  |  |  |  |  | Ipi | NA | 453 | 54 (18–86) | 269 (59.4) | 19.5 |
| **Wolchok,2017** | CheckMate 067 | NCT01844505 | Melanoma | skin | global | 1 | III or stage IV | Niv+Ipi | NA | 314 | 61 (18‒88) | 206 (66) | 38 |
|  |  |  |  |  |  |  |  | Niv | NA | 316 | 60 (25‒90) | 202 (64) | 35.7 |
|  |  |  |  |  |  |  |  | Ipi | NA | 315 | 62 (18‒89) | 202 (64) | 18.6 |
| **Eggermont,2018** | (EORTC) 1325 (KEYNOTE-054) | NCT02362594 | Melanoma | skin | global | 1 | Stage III | Pem | NA | 514 | 54 (19–88) | 324 (63.0) | 15 |
|  |  |  |  |  |  |  |  | Placebo | NA | 505 | 54 (19–83) | 304 (60.2) | 15 |
| **Long,2019** | ECHO-301/KEYNOTE-252 | NCT02752074 | unresectable or metastatic Melanoma | skin | 23 countries | all | III or IV | Epa+Pem | NA | 354 | 64 (52−72) | 217(61%) | 12.4 |
|  |  |  |  |  |  |  |  | placebo+Pem | NA | 352 | 63 (53·5−72) | 206(59%) | 12.4 |
| **Antonia,2017** | PACIFIC | NCT02125461 | NSCLC | respiratory | global | ≥2 | III | Dur | NA | 476 | 64（31-84） | 64(23-90) | 14.5 |
|  |  |  |  |  |  |  |  | Placebo | NA | 237 | 334(70.2) | 166(70) | 14.5 |
| **Bang,2018** | JAVELIN Gastric 300 | NCT02625623 | gastric or gastro-oesophageal junction cancer | digestive | 147 sites | 3 | advanced | Avelumab | NA | 185 | 59 (29–86) | 140 (75.7) | 10.6 |
|  |  |  |  |  |  |  |  | Chemo | NA | 186 | 61 (18–82) | 127 (68.3) | 10.6 |
| **Hellenmen,2018** | CheckMate 227 | 2477826 | NSCLC | respiratory | global | 1 | stage IV or recurrent | Niv +Ipi | NA | 583 | 64(26-87) | 391(67) | 11.2 |
|  |  |  |  |  |  |  |  | Niv | NA | 396 | 64(27-85) | 273(68) | 11.2 |
|  |  |  |  |  |  |  |  | Chemo | NA | 583 | 64 | 385(66) | 11.2 |
| **Wu,2019** | CheckMate 078 | 2613507 | NSCLC | respiratory | China, Russia, and Singapore | ≥2 | stage IIIB or IV or recurrent | Niv | NA | 338 | 60(27-78) | 263(78) | 10.4 |
|  |  |  |  |  |  |  |  | Doc | NA | 166 | 60(38-78) | 134(81) | 8.8 |
| **2Reck,2016** | CA184-156 | NCT01450761 | Extensive-Stage SCLC | respiratory | global | all | extensive-stage | Ipi+Chemo | NA | 478 | 62(39-85) | 317(66) | 10.5 |
|  |  |  |  |  |  |  |  | Chemo | NA | 476 | 63(36-81) | 326(68) | 10.2 |
| **Schmid,2020** | KEYNOTE-522 | NCT03036488 | Early Triple-Negative Breast Cancer |  | unkown | 1 | II or III | Pem+Chemo | NA | 784 | 49 (22–80) | NA | 15.5 |
|  |  |  |  | other |  |  |  | Chemo | NA | 390 | 48 (24–79) | NA | 15.5 |
| **2Rini,2019** | KEYNOTE-426 | NCT02853331 | Renal-Cell Carcinoma | urogenital | global | 1 | stage IV | Pem+Axitinib | 66 (15.3) | 432 | 62 (30–89) | 308(71.3) | 12.8 |
|  |  |  |  |  |  |  |  | Sunitinib | 71 (16.6) | 429 | 61 (26–90) | 320 (74.6) | 12.8 |
| **2Schmid,2020** | IMpassion130 | NCT02425891 | unresectable, locally advanced or metastatic triple-negative breast cancer | other | 41 countries | all | unresectable, locally advanced or metastatic | Ate+Chemo | 126 (28%) | 451 | 55 (46–64) | 3 (1%) | 18.5 |
|  |  |  |  |  |  |  |  | Chemo | 118 (26%) | 451 | 56 (47–65) | 1 (<1%) | 17.5 |
| **Socinski,2018** | IMpower150 | NCT2366143 | Metastatic Nonsquamous NSCLC | respiratory | unkown | 1 | stage IV or recurrent | Ate+TTD+Chemo | 347 (86.8) | 400 | 63 (31−89) | 240 (60.0) | 20 |
|  |  |  |  |  |  |  |  | TTD+Chemo | 343 (85.8) | 400 | 63 (31−90) | 239 (59.8) | 20 |
| **Kojima,2019** | KEYNOTE-181 | NCT02564263 | Advanced Esophageal Cancer | digestive | global | all | etastatic or locally advanced, unresectable | Pem | NA | 314 | 63.0 (23-84) | 273 (86.9) | 7.1 |
|  |  |  |  |  |  |  |  | Chemo | NA | 314 | 62.0 (24-84) | 271 (86.3) | 6.9 |
| **2Finns,2020** | Imbrave-150, | NCT03434379 | Unresectable Hepatocellular Carcinoma | digestive | global | 1 | Unresectable | Ate+bevacizumab | NA | 336 | 64 (56–71) | 277 (82) | 8.9 |
|  |  |  |  |  |  |  |  | Sorafenib | NA | 165 | 66 (59–71) | 137 (83) | 8.1 |
| **Yang,2019** | CAURAL | NCT02454933 | EGFR T790M–Positive NSCLC following Previous EGFR TKI Therapy | respiratory | South Korea, Canada, and China | all | stage IIIB–IV | Osimertinib | NA | 17 | 65(41-80） | 4(24) | 23.9 |
|  |  |  |  |  |  |  |  | Osi+Dur | NA | 12 | 56(41-78) | 6(50) | 17.1 |
| **Reardon,2020** | CheckMate 143 | NCT02017717 | Recurrent Glioblastoma |  | 12 countries | ≥2 | recurrent | Niv | NA | 184 | 55.5 (22-77) | 116 (63.0) | 9.8 |
|  |  |  |  |  |  |  |  | bevacizumab | NA | 185 | 55.0 (22-76) | 119 (64.3) | 9.4 |
| **Reck,2019** | KEYNOTE-024 | NCT2142738 | NSCLC | respiratory | 16 countries | 1 | untreated stage IV | Pem | NA | 154 | 64.5 (33-90) | 92 (59.7) | 25.2 |
|  |  |  |  |  |  |  |  | Chemo | NA | 151 | 66.0 (38-85) | 95 (62.9) | 25.2 |
| **Horn,2018** | IMpower133 | NCT2763579 | Extensive-Stage SCLC | respiratory | 21countries | 1 | extensive-stage | Ate+Chemo | NA | 201 | 64 (28–90) | 129 (64.2) | 13.9 |
|  |  |  |  |  |  |  |  | Chemo | NA | 202 | 64 (26–87) | 132 (65.3) | 13.9 |
| **Motzer,2019** | JAVELIN Renal 101 | unkown | Advanced Renal-Cell Carcinoma | urogenital | 21 countries | 1 | Advanced | Avelumba +Axitinib | NA | 442 | 62(29–83） | 316 (71.5) | 11.6 |
|  |  |  |  |  |  |  |  | sunitinib | NA | 444 | 61.0(27–88） | 344 (77.5) | 10.7 |
| **Jotte,2020** | Impower-131 | NCT2367794 | Squamous NSCLC | respiratory | 26 countries | all | stage IV | Ate+carboplatin+paclitaxel | NA | 338 | 64(43-85) | 278(82.2) | NA |
|  |  |  |  |  |  |  |  | Ate+carboplatin+nab-paclitaxel | NA | 343 | 65(23-83) | 280(81.6) | 18.1 |
|  |  |  |  |  |  |  |  | caboplatin+nab-paclitaxel | NA | 340 | 65(38-86) | 277(81.5) | 16.1 |
| **Owonikoko2021** | **checkmate451** | NCT02538666 | Extensive-Disease SCLC | respiratory | 32 countries | ≥2 | Extensive-Disease | Niv +Ipi | 110 (39.4) | 279 | 64.0 (39-85) | 180(64.5) | 8.4 |
|  |  |  |  |  |  |  |  | Niv | 106 (37.9) | 280 | 65.0 (32-84) | 177(63) | 9.9 |
|  |  |  |  |  |  |  |  | Placebo | 109 (39.6) | 275 | 64.0 (44-84) | 175(63.6) | 9.1 |
| **NCT02481830** | **checkmate331** |  | SCLC | respiratory | unknow | ≥2 | Relapsed | Niv | NA | 282 | 61.5 | 173(61.3 | NA |
|  |  |  |  |  |  |  |  | Chemo | NA | 265 | 61.6 | 165(62.1) | NA |
| **NCT03361865** | **KEYNOTE-672/ECHO-307** |  | UC | urogenital |  |  |  | Pem+TTD | NA | 43 | 73.3 | 32(75) |  |
|  |  |  |  |  |  |  |  | Pem | NA | 49 | 72.4 | 38(77.6) |  |
| **NCT03358472** | **KEYNOTE-669/ECHO-304** |  | HNSCC | Head and Neck | unknow | unknow | unknow | Pem+TTD | NA | 34 | 62.1 | 29(85.7) |  |
|  |  |  |  |  |  |  |  | Pem | NA | 19 | 63 | 16(84.2) |  |
|  |  |  |  |  |  |  |  | Chemo | NA | 34 | 62.7 | 28(82.9) |  |
| **Shitara,2020** | **KEYNOTE-062** | NCT02494583 | Gastric Cancer | digestive | global | 1 | advanced | Pem | NA | 256 | 61(20-83) | 180(70.3) | 29.4 |
|  |  |  |  |  |  |  |  | Pem+Chemo | NA | 257 | 62(22-83) | 195(75.9) | 29.4 |
|  |  |  |  |  |  |  |  | Chemo | NA | 250 | 62.5(23-87) | 179(71.6) | 29.4 |
| **Wu,2020** | **KEYNOTE-042** | NCT03850444 | non–small-cell lung cancer | respiratory | China | 1 | PD-L1-positive locally advanced or metastatic | Pem | NA | 128 | 62(22-78) | 105(82) | 33 |
|  |  |  |  |  |  |  |  | Chemo | NA | 134 | 62(32-82) | 119(88.8) | 33 |
| **NCT02580058** | **JAVELIN OVARIAN 200** | Pujade-Lauraine2021 | ovarian cancer | others | 24 countries | ≥2 | platinum-resistant or  platinum-refractory | Ave | NA | 188 | 61(53-69.5) | NA | 18.2 |
|  |  |  |  |  |  |  |  | Ave+pegylated liposomal doxorubicin | NA | 188 | 60(53-67) | NA | 18.4 |
|  |  |  |  |  |  |  |  | PLD | NA | 190 | 60(53-69) | NA | 17.4 |
| **NCT02409355** | **IMpower111** |  | NSCLC | respiratory | unknow | unknow | unknow | Ate | NA | 4 | NA | 2(50) | NA |
|  |  |  |  |  |  |  |  | Chemo | NA | 4 | NA | 4(100) | NA |
| **NCT03260894** | **KEYNOTE-679/ECHO-302** |  | RCC | urogenital | unknow | unknow | unknow | Pem+TTD | NA | 64 | 62.9 | 44(68.8) | NA |
|  |  |  |  |  |  |  |  | TTD | NA | 63 | 62.1 | 48(76.9) | NA |
| **NCT02420821** | **IMmotion151** | rini,2019 | RCC | urogenital | 21 countries | 1 | advanced | Ate+bevacizumab | 78(82) | 454 | 62(56-69) | 317(70) | 15 |
|  |  |  |  |  |  |  |  | sunitinib | 82(18) | 461 | 60(54-66) | 352(76) | 15 |
| **NCT02454933** | **CAURAL** |  | NSCLC | respiratory | unknow | unknow | unknow | TTD | NA | 17 | 62.3 | 4(23.5) | NA |
|  |  |  |  |  |  |  |  | Dur+TTD | NA | 12 | 57.6 | 6(50) | NA |
| **NCT02279732** |  |  | NSCLC | respiratory | unknow | unknow | unknow | Ipi+Chemo | NA | 98 | 60.9 | 87(88.8) | NA |
|  |  |  |  |  |  |  |  | Chemo | NA | 106 | 59.8 | 93(87.7) | NA |
| **NCT02718417** | **JAVELIN OVARIAN 100** |  | OC | other | unknow | unknow | unknow | Ave+Chemo | NA | 328 | 58.3 | NA | NA |
|  |  |  |  |  |  |  |  | Chemo | NA | 334 | 57.1 | NA | NA |
| **NCT03273153** | IMspire170 | Gogas,2020 | BRAFV600 wild-type melanoma | skin | global | 1 | locally advanced and unresectable or metastati | Pem | 68(30.4) | 224 | 66(55-73) | 141(62.9) | 7.2 |
|  |  |  |  |  |  |  |  | Ate+TTD | 57(25.7) | 222 | 66(54-73) | 129(58.1) | 7.1 |
| Winer2021 | KEYNOTE-119 | NCT02555657 | triple-negative breast cancer | other | 31 countries | ≥2 | metastatic | Pem | NA | 312 | 50(43-59) | 0 | 31.4 |
|  |  |  |  |  |  |  |  | Chemo | NA | 310 | 53(44-61) | 2(1%) | 31.5 |
| **NCT02302807** | IMvigor211 | powlers2018 | UC | urogenital | Europe, North America, and the Asia-Pacific region | ≥2 | platinum-treated locally advanced or metastatic | Ate | 138(30) | 467 | 67(33-88) | 357(76) | 17.3 |
|  |  |  |  |  |  |  |  | Chemo | 130(28) | 464 | 67(31-84) | 361(78) | 17.3 |
| **NCT02395172** | **JAVELIN Lung 200** | Barlesi 2018 | NSCLC | respiratory | 31 countries | ≥2 | platinum-treated  advanced | Avelumab | NA | 396 | 64(58-69) | 269(68) | 18.9 |
|  |  |  |  |  |  |  |  | docetaxel | NA | 396 | 63(57-69) | 273(69) | 17.8 |
| **NCT01057810** |  | Beer2016 | Chemotherapy-Naive Castration-Resistant Prostate Cancer | urogenital | global | 1 | Minimally Symptomatic Patients With Metastatic | Ipi | NA | 400 | 70(44-91) | NA | 24 |
|  |  |  |  |  |  |  |  | Placebo | NA | 202 | 69(42-92) | NA | 24 |
| **NCT02358031** | **MK-3475-048/KEYNOTE-048** | Burtness 2019 | squamous cell carcinoma of the head and neck | head and neck | 37 countries | 1 | recurrent or metastatic | Pem | NA | 301 | 62(56-68) | 250(83) | 11.5 |
|  |  |  |  |  |  |  |  | Pem+Chemo | NA | 281 | 61(55-68) | 224(80) | 13 |
|  |  |  |  |  |  |  |  | Chemo+TTD | NA | 300 | 61(54.5-68.0) | 261(87) | 10.7 |
| **NCT00324155** |  | Mario2015,robert2011 | Melanoma | skin | global | 1 | stage IIIc, N3 (unresectable), or stage IV | Ipi + Dacarbazine | NA | 250 | 57.5 | 152(60.8) | 54 |
|  |  |  |  |  |  |  |  | Placebo + Dacarbazine | NA | 252 | 56.4 | 149(59.1) | 54 |
| **NCT01668784** | **CheckMate 025** | Motzer2015 | Renal Cell Carcinoma | urogenital | global | ≥2 | advanced | Niv | 100(24) | 410 | 62（23-88） | 315(77) | minimum15 |
|  |  |  |  |  |  |  |  | everolimus | 87(21) | 411 | 62（18-86） | 304(74) | minimum15 |
| **NCT02231749** | checkmate214 | Motzer2018 | Renal-Cell Carcinoma | urogenital | 28 countries | 1 | advanced | Niv+IPI | 99(18) | 550 | 62(26-85) | 413(75) | 25.2 |
|  |  |  |  |  |  |  |  | sunitinib | 107(20) | 546 | 62(21-85) | 395(72) | 25.2 |
| **NCT03043872** | CASPIAN | Paz-Ares2019 | SCLC | respiratory | 23 countries | 1 | stage IV | Dur + Tre + Chemo | NA |  |  |  |  |
|  |  |  |  |  |  |  |  | Dur + Chemo | NA | 268 | 62(58-68) | 190(71) | 14.2 |
|  |  |  |  |  |  |  |  | Chemo | NA | 269 | 63(57-68) | 184(68) | 14.2 |
| **NCT02367781** | **IMpower130** | West2019 | non-squamous non-small-cell lung cancer | respiratory | eight countries | 1 | stage IV | Ate+carboplatin plus nab-paclitaxel | NA | 483 | 64(18-86) | 277 (57%) | 18.5 |
|  |  |  |  |  |  |  |  | Chemo | NA | 240 | 65(38-85) | 138 (58%) | 18.8 |
| **NCT02352948** | ARCTIC | Planchard2020 | NSCLC | respiratory | 26 countries | ≥2 | IIIB/IV locally advanced or metastic | A:Dur | NA | 62 | 63.5 (35–79) | 42 (67.7) | 9.1 |
|  |  |  |  |  |  |  |  | Soc | NA | 64 | 62.0 (41–81) | 48 (75.0) | 9.1 |
|  |  |  |  |  |  |  |  | B:Dur+Tre | NA | 174 | 62.5 (26–81) | 115 (66.1) | 9.1 |
|  |  |  |  |  |  |  |  | B:Soc | NA | 118 | 65.0 (42–83) | 81 (68.6) | 9.1 |
|  |  |  |  |  |  |  |  | B:Dur | NA | 117 | 63.0 (19–83) | 73 (62.4) | 9.1 |
|  |  |  |  |  |  |  |  | B:Tre | NA | 60 | 63.5 (45–81) | 39 (65.0) | 9.1 |
| **Bellmunt,2021** | IMvigor010 | NCT02450331 | muscle-invasive urothelial carcinoma | urogenital | 24 countries | all | M0 and  either ypT2–4a or ypN+ | Ate | NA | 406 | 67 (60–72) | 322 (79%) | 21.9 |
|  |  |  |  |  |  |  |  | placebo | NA | 403 | 66 (60–73) | 316 (78%) | 21.9 |
| **Bajorin,2021** | checkmate274 | NCT02632409 | Muscle-Invasive Urothelial Carcinoma | urogenital | 29countries | all | a high risk of recurrence | Niv | NA | 353 | 65.3 (30–92) | 265 (75.1) | 20.9 |
|  |  |  |  |  |  |  |  | Placebo | NA | 356 | 65.9 (42–88) | 275 (77.2) | 19.5 |
| **Baas,2021** | CheckMate 743 | [NCT02899299](https://clinicaltrials.gov/show/NCT02899299) | malignant pleural mesothelioma | other | 21 countries | 1 | unresectable | Niv+Ipi | NA | 303 | 69 (65–75) | 234 (77%) | 29.7 |
|  |  |  |  |  |  |  |  | Chemo | NA | 302 | 69 (62–75) | 233 (77%) | 29.7 |
| **Fennell,2021** | CONFIRM | NCT03063450 | relapsed malignant mesothelioma | other | UK | ≥2 | relapsed | Niv | NA | 221 | 70 (65–74) | 167 (76%) | 11.6 |
|  |  |  |  |  |  |  |  | Placebo | NA | 111 | 71 (65–76) | 86 (78%) | 11.6 |
| **NCT03142334** | keynote-564 | Choueiri2021 | renal cell carcinoma | urogenital | unknow | unknow | unknow | Pem | NA | 488 | NA | NA | NA |
|  |  |  |  |  |  |  |  | Placebo | NA | 496 | NA | NA | NA |
| [**NCT02611960**](https://clinicaltrials.gov/show/NCT02611960) | KEYNOTE-122 | Chan2021 | recurrent or metastatic (R/M) nasopharyngeal carcinoma | head and neck | unknow | unknow | unknow | Pem | NA | 116 | NA | NA | NA |
|  |  |  |  |  |  |  |  | Chemo | NA | 112 | NA | NA | NA |
| **Boyer,2021** | KEYNOTE-598 | NCT03302234 | Non–Small-Cell Lung Cancer With PD- L1 Tumor Proportion Score ‡ 50% | respiratory | 24 countries | 1 | stage IV | Pem + Ipi | NA | 284 | 64 (35-85) | 202 (71.1) | 20.6 |
|  |  |  |  |  |  |  |  | Pem+Placebo | NA | 284 | 65 (35-85) | 191 (67.3) | 20.6 |
| **Usmani,2019** | KEYNOTE-185 | NCT02579863 | treatment-naive multiple myeloma | hematologic | 15 countries | 1 | newly diagnosed,  treatment-naive, active multiple | Pem plus lenalidomide and dexamethasone dexamethasone | NA | 151 | 74 (70–79) | 70 (46%) | 6.6 |
|  |  |  |  |  |  |  |  | lenalidomide and dexamethasone | NA | 150 | 74 (70–78) | 71 (47%) | 6.6 |
| **Powels,2021** | keynote-361 | NCT02853305 | advanced urothelial carcinoma | urogenital | 21 | 1 | locally advanced,  unresectable or metastatic | Pem+Chemo | 78 (22%) | 351 | 69 (62–75) | 272 (78%) | 31.7 |
|  |  |  |  |  |  |  |  | Pem | 65 (21%) | 307 | 68 (61–74) | 228 (74%) | 31.7 |
|  |  |  |  |  |  |  |  | Chemo | 74 (21%) | 352 | 69 (61–75) | 262 (74%) | 31.7 |
| **NCT03066778** | Keynote-604 | Charles2020 | Extensive Stage Small Cell Lung Cancer | respiratory | 18 | 1 | stage IV | Pem + Etoposide | 95 (41.7) | 228 | 64 (24-81) | 152 (66.7) | 4.2 |
|  |  |  |  |  |  |  |  | plecbo+etoposide | 92 (40.9) | 225 | 65 (37-83) | 142 (63.1) | 3.7 |
| [**NCT02576977**](https://clinicaltrials.gov/show/NCT02576977) | keynote-183 | Mateos2019 | Refractory or Relapsed and Refractory Multiple Myeloma | hematologic | 11 countries | ≥2 | active multiple | Pem+Pomalidomide+Dexamethasone | NA | 125 | 65 (60–72) | 77 (62%) | 8.1 |
|  |  |  |  |  |  |  |  | Pomalidomide+Dexamethasone | NA | 124 | 67 (60–74) | 78 (63%) | 8.1 |
| **NCT02908672** | IMspire150 |  | Metastatic or Unresectable Locally Advanced Melanoma | skin | unknow | unknow | unknow | Cobimetinib + Vemurafenib | NA | 281 | NA | NA | NA |
|  |  |  |  |  |  |  |  | Ate+ Cobimetinib + Vemurafenib | NA | 230 |  |  |  |
| **NCT02409342** | Impower110 | herbst2020(附录有数据） | Non-Squamous or Squamous Non-Small Cell Lung Cancer | respiratory | 19 countries | 1 | Stage IV | Ate | NA | 277 | 64 (30–81) | 196 (70.8) | 13.4 |
|  |  |  |  |  |  |  |  | Chemo | NA | 277 | 65 (30–87) | 193 (69.7) | 13.4 |
| **Lee,2021** | JAVELIN head and neck 100 | NCT02952586 | squamous cell carcinoma of the head and neck | head and neck | 22 countries | 1 | locally advanced | Avelumab +Chemo | NA | 350 | 60 (54–65) | 290 (83%) | 14.6 |
|  |  |  |  |  |  |  |  | Chemo | NA | 347 | 59 (54–65) | 285 (82%) | 14.8 |
| **NCT02576509** | checkmate459 |  | Advanced Hepatocellular Carcinoma | digestive | unknow | unknow | unknow | Niv | NA | 367 | NA | NA | NA |
|  |  |  |  |  |  |  |  | SORAFENIB | NA | 363 | NA | NA | NA |
| **NCT03016312** | IMbassador250 |  | Metastatic Castration-Resistant Prostrate Cancer (mCRPC) | urogenital | unknow | unknow | unknow | Atezolizumab + Enzalutamide | NA | 386 | NA | NA | NA |
|  |  |  |  |  |  |  |  | Enzalutamide | NA | 376 | NA | NA | NA |
| Sun2021 | KEYNOTE‐590 | NCT03189719 | Esophageal Carcinoma | digestive | 26 countries | 1 | locally advanced, unresectable or  metastatic | Pem+Chemo | NA | 373 | 64 (28–94) | 306 (82%) | 22.6 |
|  |  |  |  |  |  |  |  | Chemo | NA | 376 | 62 (27–89) | 319 (85%) | 22.6 |
| **miles2021** | IMpassion131 | NCT03125902 | triple-negative breast cancer | other | global | 1 | unresectable locally advanced/metastatic | Chemo | 61(28) | 220 | 53(25-81) | 0 | 14.5 |
|  |  |  |  |  |  |  |  | Ate | 118(27) | 431 | 54(22-85) | 1(<1) | 14.2 |
| **NCT03517449** | KEYNOTE-775 |  | Advanced Endometrial Cancer | other | unknow | unknow | unknow | Lenvatinib 20 mg + Pem 200 mg | NA | 406 | NA | NA | NA |
|  |  |  |  |  |  |  |  | Chemo | NA | 388 | NA | NA | NA |
| **NCT02785952** | Lung-MAP S1400I | gettinger2021（PMC） | Recurrent Stage IV Squamous Cell Lung Cancer | respiratory |  |  |  | Nivolumab + Ipi | NA | 124 | NA | NA | NA |
|  |  |  |  |  |  |  |  | Niv | NA | 123 | NA | NA | NA |
| **Choueiri,2021** | checkmate9ER | NCT03141177 | Advanced  Renal-Cell Carcinoma | urogenital | 18 countries | 1 | Advanced | Nivolumab plus Cabozantinib | 73(22.6) | 323 | 62 (29–90) | 249 (77.1) | 18.1 |
|  |  |  |  |  |  |  |  | Sunitinib | 53(16.2) | 328 | 61 (28–86) | 232 (70.7) | 18.1 |
| **Cortes,2020** | keynote355 | NCT02819518 | previously untreated locally recurrent  inoperable or metastatic triple-negative breast cancer | othrer | 29 countries | 1 | locally recurrent  inoperable or metastatic | Pem plus Chemo Chemotherapy | 171(30) | 566 | 53 (44–63) | NA | 25.9 |
|  |  |  |  |  |  |  |  | Chemotherapy | 78(28) | 281 | 53 (43–63) | NA | 26.3 |
| **Hamanishi,2021** | NINJA |  | Platinum-Resistant Ovarian Cancer | other | global | ≥2 | advanced or recurrent | Niv | NA | 157 | 58(29-84) | NA | 1 |
|  |  |  |  |  |  |  |  | GEM or PLD | NA | 159 | 60(34-80) | NA | 1 |
| **Janjigian,2021** | checkmate649 | NCT02872116 | gastric, gastro-oesophageal junction, and oesophageal adenocarcinoma | digestive | 29 | 1 | advanced | Niv plus Chemo Chemo | 301(38) | 789 | 62 (54–69) | 540 (68%) | minimum12.1 |
|  |  |  |  |  |  |  |  | Chemo | 314(40) | 792 | 61(53-68) | 560 (71%) | minimum12.1 |
| **Kuruvilla,2021** | keynote204 | NCT02684292 | classical Hodgkin lymphoma | hematologic | 20 | all | relapsed or refractory | Pem | NA | 151 | 36 (28–53) | 84 (56%) | 25.7 |
|  |  |  |  |  |  |  |  | brentuximab vedotin | NA | 153 | 35 (28–50) | 90 (59%) | 25.7 |
| **Mai,2021** |  | NCT03581786 | advanced nasopharyngeal  carcinoma | other | mainland China, Taiwan and Singapore | 1 | advanced | Toripalimab+GP | 61(42) | 146 | 46(19-72) | 124(85) | 17.9 |
|  |  |  |  |  |  |  |  | placebo +GP | 57(40) | 143 | 51(21-72) | 116(81) | 17.4 |
| **Mittendorf,2020** | Impassion031 | NCT03197935 | triple-negative breast cancer | other | 13 | 1 | early-stage | Ate+Chemo | NA | 165 | 51 (22–76) | NA | 20.6 |
|  |  |  |  |  |  |  |  | Chemo | NA | 168 | 51 (26–78) | NA | 19.8 |
| NCT02625610 | JAVELIN Gastric 100, | Moehler,2021 | Maintenance Gastric Cancer | digestive | 17 | 1 | advanced or metastatic | Ave | NA | 249 | 62 | 164(65.9) | 24.1 |
|  |  |  |  |  |  |  |  | Chemo | NA | 250 | 61 | 167(66.8) | 24 |
| **Paz-Ares,2021** | checkmare 9LA | NCT03215706 | non-small-cell lung cancer | respiratory | 19 | 1 | stage IV or recurrent | Niv+Ipi+Chemo | 68(19) | 361 | 65(59-70) | 109(30) | 9.7 |
|  |  |  |  |  |  |  |  | Chemo | 86(24) | 358 | 65(58-70) | 106(30) | 9.7 |
| **Powels,2020** | DANUBE | NCT02516241 | unresectable, locally advanced or metastatic urothelial carcinoma | urogenital | 23 | 1 | unresectable, locally advanced or metastatic | Dur | NA | 346 | 67 (60–73) | 249 (72%) | 41.2 |
|  |  |  |  |  |  |  |  | Dur+Treme | NA | 342 | 68 (60–73) | 256 (75%) | 41.2 |
|  |  |  |  |  |  |  |  | Chemo | NA | 344 | 68 (60–73) | 274 (80%) | 41.2 |
| **Sezer,2021** | Empwer-lung 1 | NCT03088540 | advanced non-small-cell lung cancer | respiratory | 24 | 1 | advanced | Cemiplimab | NA | 356 | 63 (58–69) | 312(88) | 13.1 |
|  |  |  |  |  |  |  |  | Chemo | NA | 354 | 64(57-69) | 294(83) | 13.1 |
| **Sugawara,2021** | ono-4538-52/tasuki-52 | NCT03117049 | advanced nonsquamous non-small-cell lung cancer | respiratory | unknow | 1 | stage IIIB/ IV or recurrent | Niv | NA | 275 | 66(27-85) | 205(74.5) | 13.7 |
|  |  |  |  |  |  |  |  | Placebo | NA | 275 | 66(33-83) | 206(74.9) | 13.7 |
| **Wang,2021** |  | NCT03594747 | Advanced Squamous Non–Small-Cell Lung Cancer | respiratory | China | 1 | advanced | Tislelizumab Plus Chemo | 15(12.5) | 120 | 60 (41-74) | 107 (89.2) | 8.6 |
|  |  |  |  |  |  |  |  | Tislelizumab Plus nab-PC | 15(12.6) | 119 | 63 (38-74) | 112 (94.1) | 8.6 |
|  |  |  |  |  |  |  |  | PC | 14(11.6) | 121 | 62 (34-74) | 111 (91.7) | 8.6 |
| **Yang,2020** |  | NCT03607539 | Locally Advanced or Metastatic Nonsquamous NSCLC | respiratory | China | 1 | stage IIIB to IV | Sintilimab | NA | 266 | 61(30-75) | 204 (76.7) | 8.9 |
|  |  |  |  |  |  |  |  | Placebo | NA | 131 | 61(35-75) | 99 (75.6) | 8.9 |
| **Yang,2021** | captain-1st | NCT03707509 | recurrent or metastatic nasopharyngeal carcinoma | head and neck | China | 1 | recurrent  or metastatic | Camrelizumab+gemcitabine and cisplatin | 70(52) | 134 | 52 (40–58) | 113 (84%) | 15.6 |
|  |  |  |  |  |  |  |  | gemcitabine and cisplatin | 66(51) | 129 | 49 (40–56) | 105 (81%) | 15.6 |
| **Zhou,2021** | ORIENT-12 | NCT03629925 | Advanced or Metastatic Squamous NSCLC | respiratory | China | 1 | stage IIIB/IIIC or stage IV | Sintilimab+GP | NA | 179 | 64 (39–75) | 163 (91.1) | 12.9 |
|  |  |  |  |  |  |  |  | GP | NA | 178 | 62 (33–75) | 164 (92.1) | 12.9 |
| **2Zhou,2021** | CameL | NCT03134872 | advanced non-squamous non-small-cell lung cancer | respiratory | China | 1 | advanced | Camrelizumab plus carboplatin and pemetrexed | NA | 205 | 59 (54–64) | 146 (71%) | 11.9 |
|  |  |  |  |  |  |  |  | Chemo | NA | 207 | 61 (53–65) | 149 (72%) | 11.9 |
| NCT02811861 | keynote581 | Motzer,2021 | Advanced Renal Cell Carcinoma | urogenital | 20 | 1 | Advanced | Lenvatinib+pem | 60(16.9) | 355 | 64 (34–88) | 255 (71.8) | 26.6 |
|  |  |  |  |  |  |  |  | Lenvatinib+everolimus | 62(17.4) | 357 | 62 (32–86) | 266 (74.5) | 26.6 |
|  |  |  |  |  |  |  |  | Sunitinib | 61(17.1) | 357 | 61 (29–82) | 275 (77.0) | 26.6 |
| NCT02864394 | keynote033 | Zhou,2020 | NSCLC | respiratory | unknow | unknow | unknow | Pem | NA | 213 | NA | NA | NA |
|  |  |  |  |  |  |  |  | Chemo | NA | 198 | NA | NA | NA |

# 12 eTable 3. Network estimates of treatment comparisons for Treatment-related all-grade and grade 3-5 hepatotoxicity

## eTable 3.1 Network estimates of treatment comparisons for Treatment-related all-grade and grade 3-5 ALT

## eTable 3.2 Network estimates of treatment comparisons for Treatment-related all-grade and grade 3-5 AST

## eTable 3.3 Network estimates of treatment comparisons for Treatment-related all-grade and grade 3-5 ALP

## eTable 3.4 Network estimates of treatment comparisons for Treatment-related all-grade and grade 3-5 GGT

## eTable 3.5 Network estimates of treatment comparisons for Treatment-related all-grade and grade 3-5 Bilirubin

# 13 eTable 4. Network estimates of treatment comparisons for Immune-mediated all-grade and grade 3-5 hepatotoxicity

## eTable 4.1 Network estimates of treatment comparisons for Immune-mediated all-grade and grade 3-5 ALT

## eTable 4.2 Network estimates of treatment comparisons for Immune-mediated all-grade and grade 3-5 AST

## eTable 4.3 Network estimates of treatment comparisons for Immune-mediated all-grade and grade 3-5 GGT

## eTable 4.4 Network estimates of treatment comparisons for Immune-mediated all-grade and grade 3-5 ALP

## eTable 4.5 Network estimates of treatment comparisons for Immune-mediated all-grade and grade 3-5 Bilirubin

# 14 eTable 5 Network estimates of treatment comparisons for Fatal liver adverse events.

# 15 eTable 6. The ranking of treatment regimen on Treatment-related hepatotoxicity by cancer type

eTable 6.1 Network estimates of treatment comparisons for Treatment-related all-grade ALT and grade 3-5 ALT on respiratory system cancer.

## eTable 6.2 Network estimates of treatment comparisons for Treatment-related all-grade ALT and grade 3-5 ALT on head and neck system cancer.

## eTable 6.3 Network estimates of treatment comparisons for Treatment-related all-grade ALT and grade 3-5 ALT on skin cancer.

## eTable 6.4 Network estimates of treatment comparisons for Treatment-related all-grade ALT and grade 3-5 ALT on urogenital system cancer.

## eTable 6.5 Network estimates of treatment comparisons for Treatment-related all-grade ALT and grade 3-5 ALT on digest system cancer.

# 16 eTable 7. The results of random consistency evaluation

| **Treatment-related All-grade Results** | **Dbar** | **Pd** | **#of data points** | **Deviance information Criterion** | **ratio** | **I^2^** |
| --- | --- | --- | --- | --- | --- | --- |
| **ALT** | **178.38** | **145.68** | **175** | **324.06** | **1.019** | **2** |
| **AST** | **167.74** | **138.22** | **166** | **305.96** | **1.01** | **2** |
| **ALP** | **64.70** | **46.97** | **62** | **111.67** | **1.044** | **6** |
| **GGT** | **56.64** | **46.34** | **55** | **102.98** | **1.03** | **5** |
| **Bilirubin** | **42.58** | **36.25** | **46** | **78.83** | **0.9257** | **0** |
| **Treatment-related Grade 3-5 Results** | **Dbar** | **Pd** | **#of data points** | **Deviance information Criterion** | **ratio** | **I^2^** |
| **ALT** | **160.01** | **98.72** | **173** | **258.73** | **0.9249** | **0** |
| **AST** | **150.53** | **95.46** | **166** | **245.99** | **0.9068** | **0** |
| **ALP** | **28.73** | **22.28** | **62** | **51.01** | **0.4633** | **0** |
| **GGT** | **46.08** | **34.32** | **55** | **8.40** | **0.84** | **0** |
| **Bilirubin** | **25.39** | **20.53** | **46** | **45.93** | **0.5521** | **0** |
| **Immune-mediated All-grade Results** | **Dbar** | **Pd** | **#of data points** | **Deviance information Criterion** | **ratio** | **I^2^** |
| **ALT** | **23.83** | **21.33** | **25** | **45.16** | **0.9533** | **0** |
| **AST** | **24.42** | **21.99** | **25** | **46.42** | **0.977** | **2** |
| **ALP** | **14** | **11.02** | **13** | **25.02** | **1.077** | **14** |
| **GGT** | **15.23** | **11.65** | **13** | **26.88** | **1.172** | **21** |
| **Bilirubin** | **14.23** | **9.54** | **13** | **23.77** | **1.095** | **16** |
| **Immune-mediated Grade 3-5 Results** | **Dbar** | **Pd** | **#of data points** | **Deviance information Criterion** | **ratio** | **I^2^** |
| **ALT** | **21.18** | **18.88** | **25** | **40.06** | **0.85** | **0** |
| **AST** | **23.42** | **18.47** | **25** | **41.89** | **0.937** | **0** |
| **ALP** | **5.45** | **4.73** | **13** | **10.18** | **0.42** | **0** |
| **GGT** | **14.15** | **9.54** | **13** | **23.69** | **1.089** | **15** |
| **Bilirubin** | **14.23** | **9.56** | **13** | **23.79** | **1.094** | **16** |

# 17 eTable 8. The list of the targeted therapy drug in main cancer.

| Cancer Type | Drugs that need to be tested for targets | Drugs that do not require detection of the target |
| --- | --- | --- |
| Lung Cancer | Gefitinib  Erlotinib  Icotinib  Afatinib  Dacomitinib  Osimertinib  Crizotinib  Alectinib  Ceritinib  Savolitinib | Bevacizumab  Recombinant human endostatin  Anlotinib  Everolimus |
| Liver cancer |  | Sorafenib  Regorafenib  Lenvatinib  Donafenib  Bevacizumab |
| Gastric cancer | Trastuzumab  Vidisetuzumab | Apatinib |
| Gastrointestinal | Imatinib  Afatinib | Stivarga  Sunitinib  Ripretinib |
| Colorectal cancer | Cetuximab | Bevacizumab  Stivarga  Fruquintinib |
| Leukemia | Imatinib  Dasatinib  Erlotinib  Gilteritinib | Imbruvica  Blinatumomab  Venetoclax |
| Lymphoma | Rituximab  Brentuximab | Chidamide  Ibrutinib  Bortezomib  Zanubrutinib |
| Multiple myeloma |  | Bortezomib  Lenalidomide  Pomalidomide  Thalidomide  Ixazomib  Daratumumab |
| Prostate cancer | Olaparib |  |
| Melanoma | Imatinib  Vemurafenib  Dabrafenib  Trametinib |  |
| Nasopharyngeal carcinoma | Nimotuzumab |  |
| Squamous cell carcinoma of the head and neck |  | Cetuximab |
