# Supplementary figures and images for: Dexamethasone mitigates remdesivir-induced liver toxicity in human primary hepatocytes and COVID-19 patients
Source: Hepatol Commun. 2023 Feb 20;7(3):e0034. doi: 10.1097/HC9.0000000000000034 (PMC9949788; doi:10.1097/HC9.0000000000000034)

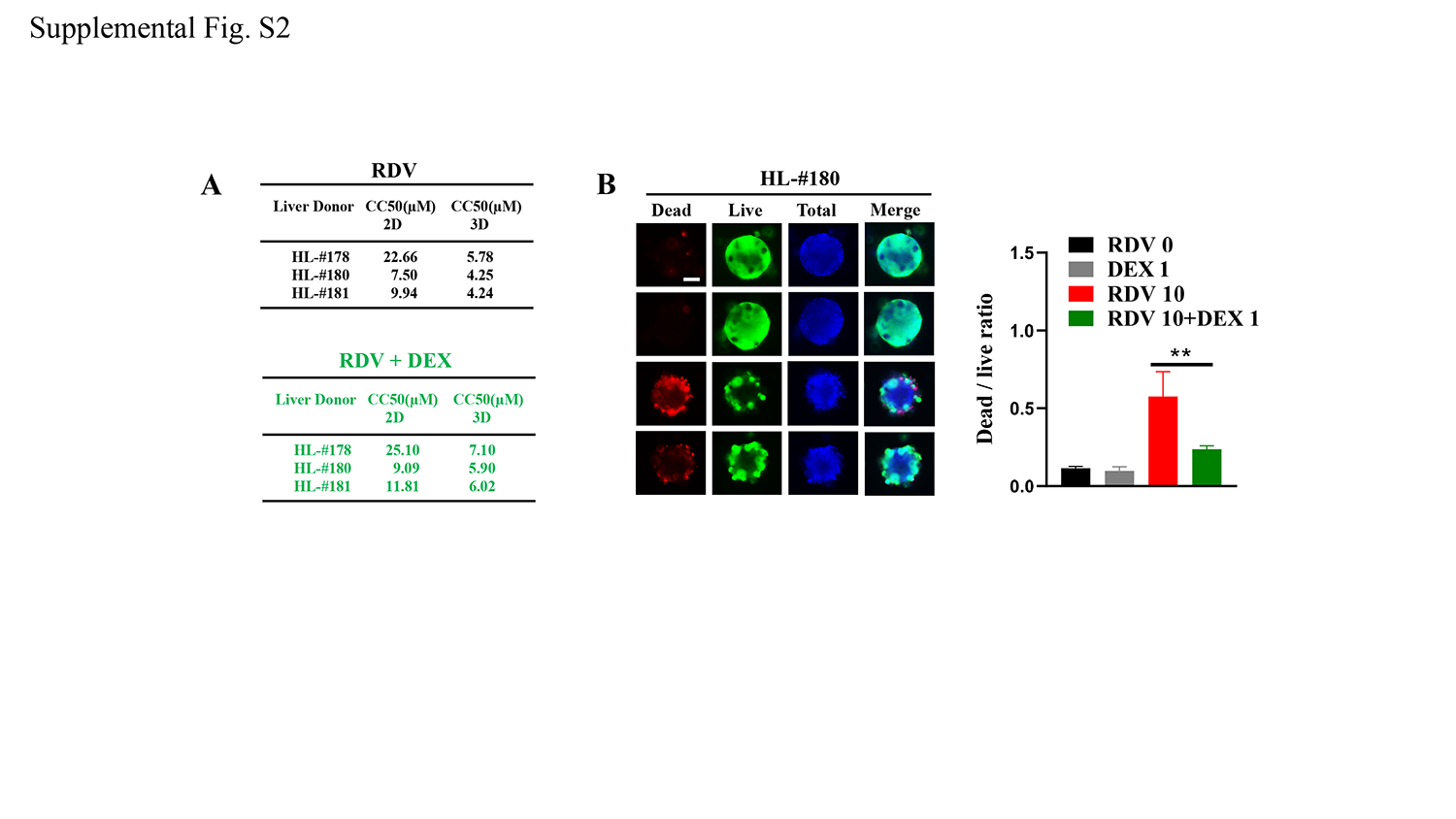

Supplement: Supplementary file 2 [file hc9-7-e0034-s002.tiff]

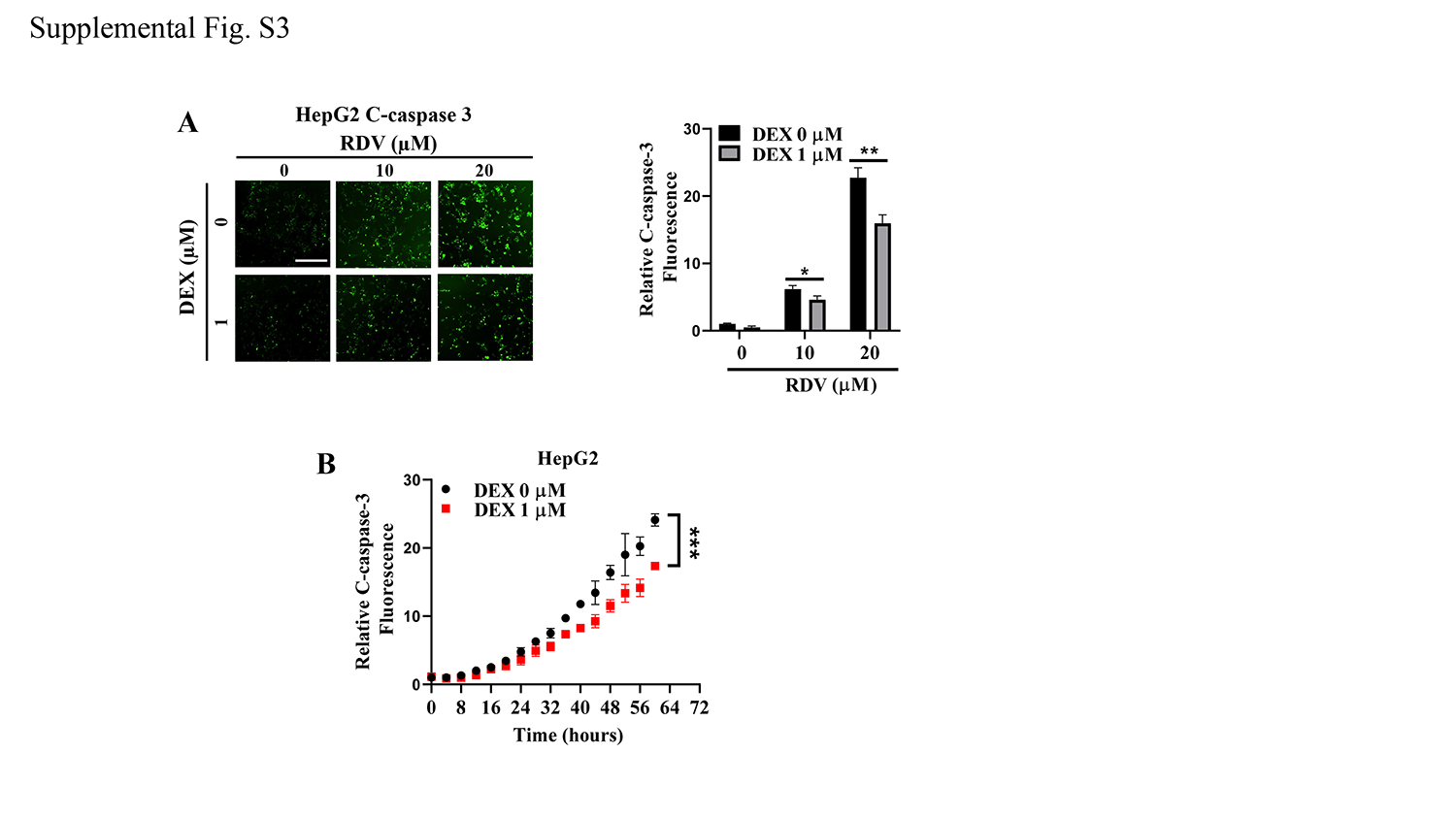

Supplement: Supplementary file 3 [file hc9-7-e0034-s003.tiff]
