# Supplementary material for: Dexamethasone mitigates remdesivir-induced liver toxicity in human primary hepatocytes and COVID-19 patients
Source: Hepatol Commun. 2023 Feb 20;7(3):e0034. doi: 10.1097/HC9.0000000000000034 (PMC9949788; doi:10.1097/HC9.0000000000000034)
Supplement: Supplementary file 4 [file hc9-7-e0034-s004.docx]

**Table S1.** Liver Donor Demographic Information

| Donor | Age (years) | Race | Sex | BMI |
| --- | --- | --- | --- | --- |
|  |  |  |  |  |
| HL#163 | 58 | Caucasian | Female | 28.1 |
| Hl#171 | 52 | Caucasian | Male | 19.7 |
| HL#175  HL#177 | 27  39 | Caucasian  Caucasian | Male  Male | 29.1  26.7 |
| HL#178 | 49 | Caucasian | Female | 22.1 |
| HL#179 | 31 | Caucasian | Male | 23.1 |
| HL#180 | 58 | Caucasian | Male | 23.9 |
| HL#181 | 42 | Caucasian | Male | 33.3 |
| HL#182 | 57 | Caucasian | Female | 17.0 |
| HL#183 | 45 | Caucasian | Male | 22.6 |
| HL#184 | 60 | Caucasian | Male | 29.0 |
| HL#186 | 65 | Caucasian | Female | 24.2 |
| HL#197 | 56 | Caucasian | Male | 29.5 |

**Table S2.** Baseline Comorbidities of Patients with COVID-19

| **Comorbidity Categories** | **Remdesivir  (n=192)** | **Remdesivir+ Dexamethasone (n=1810)** | **Total  n=2002 (n, %)** |
| --- | --- | --- | --- |
| Acquired immune deficiency syndrome | 3 (1.6%) | 9 (0.5%) | 12 (0.6%) |
| Alcohol abuse | 6 (3.1%) | 36 (1.8%) | 39 (2.0%) |
| Chronic blood loss anemia | 1 (0.5%) | 18 (1.0%) | 19 (1.0%) |
| Chronic pulmonary disease | 47 (24.5%) | 439 (24.5%) | 486 (24.5%) |
| Coagulopathy | 21 (10.9%) | 156 (8.7%) | 177 (8.9%) |
| Congestive heart failure | 27 (14.1%) | 239 (13.3%) | 266 (13.4%) |
| Deficiency Anemias* | 65 (33.9%) | 433 (24.2%) | 498 (25.1%) |
| Depression | 28 (14.6%) | 205 (11.4%) | 233 (11.7%) |
| Diabetes w/ chronic complications | 56 (29.2%) | 464 (25.9%) | 520 (26.2%) |
| Diabetes w/o chronic complications | 24 (12.5%) | 174 (9.7%) | 198 (10.0%) |
| Drug abuse | 10 (5.2%) | 55 (3.1%) | 65 (3.3%) |
| Fluid and electrolyte disorders* | 119 (62.0%) | 936 (52.2%) | 1055(53.2%) |
| Hypertension (combine uncomplicated and complicated) | 119 (62.0%) | 1197 (66.8%) | 1316(66.3%) |
| Hypothyroidism | 21 (10.9%) | 199 (11.1%) | 220 (11.1%) |
| Liver disease | 8 (4.2%) | 77 (4.3%) | 85 (4.3%) |
| Lymphoma | 1 (0.5%) | 15 (0.8%) | 16 (0.8%) |
| Metastatic cancer | 2 (1.0%) | 24 (1.3%) | 26 (1.3%) |
| Obesity | 81 (42.2%) | 722 (40.3%) | 803 (40.5%) |
| Other neurological disorders | 14 (7.3%) | 137 (7.7%) | 151 (7.6%) |
| Paralysis | 7 (3.7%) | 44 (2.5%) | 51 (2.6%) |
| Peptic ulcer Disease x bleeding | 0 (0.0%) | 8 (0.5%) | 8 (0.4%) |
| Peripheral vascular disease | 4 (2.1%) | 69 (3.9%) | 73 (3.7%) |
| Psychoses | 10 (5.2%) | 71 (4.0%) | 81 (4.1%) |
| Pulmonary circulation disease | 8 (4.2%) | 58 (3.2%) | 68 (3.3%) |
| Renal failure* | 40 (20.8%) | 185 (10.3%) | 225 (11.3%) |
| Rheumatoid arthritis/collagen vas | 2 (1.0%) | 44 (2.5%) | 46 (2.3%) |
| Solid tumor w/out metastasis | 3 (1.6%) | 30 (1.7%) | 33 (1.7%) |
| Valvular disease | 10 (5.2%) | 102 (5.7%) | 112 (5.7%) |
| Weight loss* | 17 (8.9%) | 49 (2.7%) | 66 (3.3%) |

*****= associated with the exposure, determined by X^2^

**Table S3.** Crude Association Between Covariates and Exposure

|  | **Remdesivir (n=192) (n, %)** | **Remdesivir + Dexamethasone (n=1810)** | ***p-*value** |
| --- | --- | --- | --- |
| **Race**  White  Black  Other  **Age**  **Ethnicity**  Hispanic or Latino  Not Hispanic or Latino  **Concomitant potential**  **Hepatotoxic medications**  Given  Not Given  **Liver Disease**  Yes  No  **HCV**  Positive  Negative  **HBV^T^**  Positive  Negative  **APR DRG Score**  < 3  3  4  **Renal Failure**  Yes  No  **Electrolytes and Fluid Disorders**  Yes  No  **Weight Loss**  Yes  No  **Deficiency Anemias**  Yes  No | 60 (31.3%)  82 (42.7%)  50 (25.0%)  57.3 ± 17.0  45 (23.4%)  146 (76.0%)  174 (90.6%)  18 (9.4%)  11 (5.7%)  181 (94.3%)  6 (3.1%)  186 (96.9%)  2 (1.0%)  190 (99.0%)  18 (9.4%)  54 (28.1%)  120 (62.5%)  41 (21.0%)  154 (79.0%)  119 (62.0%)  73 (38.0%)  17 (8.9%)  175 (91.2%)  65 (33.9%)  127 (66.1%) | 854 (47.2%)  740 (40.9%)  216 (11.9%)  61.1 ± 16.1  132 (7.3%)  1670 (92.3%)  1400 (77.4%)  410 (22.6%)  80 (4.4%)  1730 (95.6%)  35 (1.9%)  1775 (98.1%)  1 (0.1%)  1809 (99.9%)  96 (5.3%)  756 (41.8%)  958 (52.9%)  193 (10.4%)  1658 (89.6)  936 (52.2%)  856 (47.8%)  49 (2.7%)  1743 (97.3%)  433 (24.2%)  1359 (75.8%) | **< 0.0001**  **0.001**  **< 0.0001**  **< 0.0001**  0.41  0.27  **0.003**  **< 0.0001**  **0.01**  **< 0.0001**    **0.003** |

^T^ denotes too few cases to run X^2^

**Table S4.** Confounding Assessment

| **Cofactors** | **Association with RDV or RDV and DEX** | **Associated with AST/ALT 3 X ULN** | **% CE** |
| --- | --- | --- | --- |
| Race  Age  Ethnicity  APR DRG Severity Score  Liver toxic drugs  Liver disease  Renal Failure  Weight Loss  Electrolytes and fluid disorders  Deficiency Anemia | ✓  ✓  ✓  ✓  ✓  X  ✓  ✓  ✓ ✓ | ✓  X  ✓  ✓  X  ✓  X  X  X  X | **14%**  6%  **11%**  2%  4%  2%  2%  2%  5%  5% |

**Table S5.** Medications considered to be potentially hepatotoxic included in the assessment of number of concomitant potentially hepatotoxic medications received

| Acetaminophen | Allopurinol | Alprazolam |
| --- | --- | --- |
| Amiodarone | Amitriptyline | Amlodipine |
| Amoxicillin-clavulanate | Aripiprazole | Atenolol |
| Atomoxetine | Atorvastatin | Aztreonam |
| bupropion | carbamazepine | Carbidopa-levodopa |
| carvedilol | ceftriaxone | ciprofloxacin |
| clonidine | clonazepam | cyclosporine |
| Cyclobenzaprine | dapsone | diazepam |
| Diltiazem | duloxetine | escitalopram |
| Enoxaparin sodium | fenofibrate | fluconazole |
| Furosemide | gabapentin | hydralazine |
| Hydrocortisone | ibuprofen | isoniazid |
| Labetalol | lamotrigine | levetiracetam |
| Levofloxacin | levothyroxine | linezolid |
| Lisinopril | lithium | lorazepam |
| Losartan | lurasidone | loxapine |
| Melatonin | metformin | methadone |
| Methotrexate | methylphenidate | methylprednisolone |
| Metoprolol succinate | Metoprolol tartrate | midodrine |
| Minocycline | mirtazapine | moxifloxacin |
| Naproxen | nifedipine | nortriptyline |
| OLANZapine | oxcarbazepine | oxycodone |
| PARoxetine | Potassium chloride SA | pramipexole |
| predniSONE | pregabalin | propranolol |
| Protamine | quetiapine | rifampin |
| risperiDONE | ropinirole | rosuvastatin |
| Sertraline | simvastatin | Sulfamethoxazole-trimethoprim |
| Tacrolimus | terazosin | tobramycin |
| traZODone | valproate | Valproic acid |
| Venlafaxine | Verapamil | voriconazole |
| Vitamin C | Vitamin E | warfarin |
|  |  |  |

**Table S6.** Crude Association between the Exposure (RDV vs. RDV + DEX) and the Outcome (AST/ALT 3 X ULN) in COVID-19 patients.

| **AST and ALT** | ≥ **3 X ULN (n=70)** | **< 3 X ULN (n=1932)** | **Unadjusted OR (95% CI)** | ***p-value*** |
| --- | --- | --- | --- | --- |
| Remdesivir alone  (patient # and %)   Remdesivir + Dexamethasone (patient # and %) | 14 (7.3%)  56 (3.1%) | 178 (92.7%)  1754 (96.9%) | REF  0.41 (0.22, 0.74) | 0.004 |
|  |  |  |  |  |

**Table S7.** Adjusted Logistic Regression for the Association between the Exposure (Dexamethasone addition) and the Outcome (AST/ALT 3 X ULN) in COVID-19 patients receiving RDV treatment.

| **AST and ALT (n=2002)** | ≥ **3X ULN (n=70)** | **< 3 X ULN (n=1932)** | **Adjusted OR (95% CI)** | ***Adjusted p-value*** |
| --- | --- | --- | --- | --- |
| Remdesivir alone  (patient # and %)   Remdesivir + Dexamethasone (patient # and %) | 14 (7.3%)  56 (3.1%) | 178 (92.7%)  1754 (96.9%) | REF  0.46 (0.25, 0.85) | 0.02 |
|  |  |  |  |  |

*Determined using adjusted logistic regression for age, race, ethnicity, APR DRG score, liver disease, and presence of medications with potential hepatotoxic side effects.
